# Supplementary material for: Risk factors for fluoroquinolone- and macrolide-resistance among swine Campylobacter coli using multi-layered chain graphs
Source: PLoS Comput Biol. 2025 Aug 13;21(8):e1012797. doi: 10.1371/journal.pcbi.1012797 (PMC12373276; doi:10.1371/journal.pcbi.1012797)
Supplement: S3 Table — A document with a summary of MIC distributions and isolate counts by cohort. (DOCX) [file pcbi.1012797.s005.docx]

**SUPPLEMENTAL MATERIAL S6**

The following tables summarize the isolate counts, MIC distributions, and count and proportion of isolates with MICs equal to or greater than resistance breakpoints for *Campylobacter coli* isolates collected from 14 swine cohorts. Breakpoints were taken from the [CDC NARMS website](https://www.cdc.gov/narms/about/antibiotics-tested.html), which were based on EUCAST ecoffs except telithromycin. Blue shaded regions of the tables represent tested concentrations and red shaded regions represent isolates with MICs that exceeded all tested concentrations, but these interpreted values were not used in the study. Vertical lines represent the breakpoints for the respective antibiotic (ERY Resistance ≥ 16μg/ml, TEL Resistance ≥ 8μg/ml, AZI Resistance ≥ 1μg/ml, CLI Resistance ≥ 2μg/ml, NAL Resistance ≥ 64μg/ml, CIP Resistance ≥ 1μg/ml, GEN Resistance ≥ 4μg/ml, TET Resistance ≥ 4μg/ml, FFN Resistance ≥ 8μg/ml)

**S6-T1 – Cohort A.** Conventional production. 77 isolates (59 fecal, 7 feed or water, 11 environmental)

| **Drug** | **Concentration (μg/ml)** | | | | | | | | | | | | | | **Median MIC** | **n_resistant_ (%)** |
| --- | --- | --- | --- | --- | --- | --- | --- | --- | --- | --- | --- | --- | --- | --- | --- | --- |
|  | 0.015 | 0.03 | 0.06 | 0.12 | 0.25 | 0.5 | 1 | 2 | 4 | 8 | 16 | 32 | 64 | >64 |  |  |
| ERY |  | 1 | 0 | 0 | 1 | 15 | 30 | 6 | 0 | 0 | 0 | 0 | 0 | 24 | 1 | 24 (31%) |
| TEL | 1 | 0 | 0 | 0 | 0 | 7 | 17 | 26 | 3 | 6 | 17 |  |  |  | 2 | 23 (30%) |
| AZI | 1 | 1 | 18 | 31 | 1 | 0 | 1 | 0 | 0 | 0 | 0 | 0 | 0 | 24 | 0.12 | 25 (32%) |
| CLI |  | 1 | 0 | 12 | 33 | 5 | 2 | 10 | 14 | 0 | 0 | 0 |  |  | 0.25 | 24 (31%) |
| NAL |  |  |  |  |  |  |  |  | 25 | 27 | 0 | 1 | 10 | 14 | 8 | 24 (31%) |
| CIP | 2 | 0 | 9 | 39 | 2 | 0 | 0 | 1 | 1 | 8 | 12 | 3 | 0 | 0 | 0.12 | 25 (32%) |
| GEN |  |  |  | 2 | 0 | 37 | 37 | 1 | 0 | 0 | 0 | 0 | 0 |  | 0.5 | 0 (0%) |
| TET |  |  | 1 | 0 | 1 | 0 | 0 | 0 | 1 | 2 | 6 | 7 | 29 | 30 | 64 | 75 (97%) |
| FFN |  | 1 | 0 | 0 | 0 | 18 | 53 | 5 | 0 | 0 | 0 | 0 | 0 | 0 | 1 | 0 (0%) |

**S6-T2 – Cohort B.** Conventional production. 50 isolates (33 fecal, 8 feed or water, 9 environmental)

| **Drug** | **Concentration (μg/ml)** | | | | | | | | | | | | | | **Median MIC** | **n_resistant_ (%)** |
| --- | --- | --- | --- | --- | --- | --- | --- | --- | --- | --- | --- | --- | --- | --- | --- | --- |
|  | 0.015 | 0.03 | 0.06 | 0.12 | 0.25 | 0.5 | 1 | 2 | 4 | 8 | 16 | 32 | 64 | >64 |  |  |
| ERY |  | 0 | 0 | 0 | 0 | 9 | 30 | 3 | 0 | 1 | 0 | 1 | 0 | 6 | 1 | 7 (14%) |
| TEL | 0 | 0 | 0 | 0 | 0 | 2 | 13 | 25 | 4 | 2 | 4 |  |  |  | 2 | 6 (12%) |
| AZI | 0 | 1 | 10 | 28 | 3 | 0 | 1 | 0 | 0 | 0 | 0 | 0 | 0 | 7 | 0.12 | 8 (16%) |
| CLI |  | 0 | 0 | 9 | 28 | 7 | 0 | 0 | 4 | 1 | 1 | 0 |  |  | 0.25 | 6 (12%) |
| NAL |  |  |  |  |  |  |  |  | 17 | 32 | 1 | 0 | 0 | 0 | 8 | 0 (0%) |
| CIP | 0 | 0 | 7 | 42 | 1 | 0 | 0 | 0 | 0 | 0 | 0 | 0 | 0 | 0 | 0.12 | 0 (0%) |
| GEN |  |  |  | 0 | 1 | 21 | 28 | 0 | 0 | 0 | 0 | 0 | 0 |  | 1 | 0 (0%) |
| TET |  |  | 0 | 0 | 0 | 0 | 0 | 0 | 0 | 3 | 2 | 2 | 23 | 20 | 64 | 50 (100%) |
| FFN |  | 0 | 0 | 0 | 0 | 3 | 45 | 2 | 0 | 0 | 0 | 0 | 0 | 0 | 1 | 0 (0%) |

**S6-T3 – Cohort C.** Conventional production. 62 isolates (all fecal)

| **Drug** | **Concentration (μg/ml)** | | | | | | | | | | | | | | **Median MIC** | **n_resistant_ (%)** |
| --- | --- | --- | --- | --- | --- | --- | --- | --- | --- | --- | --- | --- | --- | --- | --- | --- |
|  | 0.015 | 0.03 | 0.06 | 0.12 | 0.25 | 0.5 | 1 | 2 | 4 | 8 | 16 | 32 | 64 | >64 |  |  |
| ERY |  | 1 | 0 | 0 | 1 | 19 | 9 | 5 | 0 | 0 | 0 | 0 | 0 | 27 | 2 | 27 (44%) |
| TEL | 1 | 0 | 0 | 0 | 0 | 2 | 16 | 14 | 3 | 2 | 24 |  |  |  | 2 | 26 (42%) |
| AZI | 1 | 1 | 18 | 10 | 5 | 0 | 0 | 0 | 0 | 0 | 0 | 0 | 0 | 27 | 0.25 | 27 (44%) |
| CLI |  | 1 | 0 | 10 | 16 | 5 | 2 | 6 | 17 | 5 | 0 | 0 |  |  | 0.5 | 28 (45%) |
| NAL |  |  |  |  |  |  |  |  | 17 | 45 | 0 | 0 | 0 | 0 | 8 | 0 (0%) |
| CIP | 1 | 0 | 18 | 37 | 6 | 0 | 0 | 0 | 0 | 0 | 0 | 0 | 0 | 0 | 0.12 | 0 (0%) |
| GEN |  |  |  | 1 | 2 | 17 | 37 | 5 | 0 | 0 | 0 | 0 | 0 |  | 1 | 0 (0%) |
| TET |  |  | 1 | 0 | 0 | 0 | 3 | 0 | 0 | 4 | 8 | 5 | 17 | 24 | 64 | 58 (94%) |
| FFN |  | 1 | 0 | 0 | 1 | 14 | 41 | 5 | 0 | 0 | 0 | 0 | 0 | 0 | 1 | 0 (0%) |

**S6-T4 – Cohort D.** Conventional production. 86 isolates (40 fecal, 17 feed or water, 29 environmental)

| **Drug** | **Concentration (μg/ml)** | | | | | | | | | | | | | | **Median MIC** | **n_resistant_ (%)** |
| --- | --- | --- | --- | --- | --- | --- | --- | --- | --- | --- | --- | --- | --- | --- | --- | --- |
|  | 0.015 | 0.03 | 0.06 | 0.12 | 0.25 | 0.5 | 1 | 2 | 4 | 8 | 16 | 32 | 64 | >64 |  |  |
| ERY |  | 5 | 0 | 0 | 3 | 39 | 30 | 2 | 0 | 0 | 0 | 0 | 0 | 7 | 0.5 | 7 (8%) |
| TEL | 5 | 0 | 0 | 0 | 1 | 5 | 41 | 26 | 1 | 2 | 5 |  |  |  | 1 | 7 (8%) |
| AZI | 5 | 1 | 38 | 33 | 2 | 0 | 0 | 0 | 0 | 0 | 0 | 0 | 0 | 7 | 0.06 | 7 (8%) |
| CLI |  | 5 | 0 | 24 | 46 | 4 | 0 | 2 | 3 | 2 | 0 | 0 |  |  | 0.25 | 7 (8%) |
| NAL |  |  |  |  |  |  |  |  | 53 | 33 | 0 | 0 | 0 | 0 | 4 | 0 (0%) |
| CIP | 5 | 0 | 14 | 57 | 10 | 0 | 0 | 0 | 0 | 0 | 0 | 0 | 0 | 0 | 0.12 | 0 (0%) |
| GEN |  |  |  | 5 | 4 | 37 | 40 | 0 | 0 | 0 | 0 | 0 | 0 |  | 0.5 | 0 (0%) |
| TET |  |  | 5 | 0 | 0 | 1 | 0 | 2 | 3 | 5 | 11 | 8 | 30 | 21 | 64 | 78 (91%) |
| FFN |  | 5 | 0 | 0 | 0 | 11 | 67 | 3 | 0 | 0 | 0 | 0 | 0 | 0 | 1 | 0 (0%) |

**S6-T5 – Cohort E.** Conventional production. 98 isolates (all fecal)

| **Drug** | **Concentration (μg/ml)** | | | | | | | | | | | | | | **Median MIC** | **n_resistant_ (%)** |
| --- | --- | --- | --- | --- | --- | --- | --- | --- | --- | --- | --- | --- | --- | --- | --- | --- |
|  | 0.015 | 0.03 | 0.06 | 0.12 | 0.25 | 0.5 | 1 | 2 | 4 | 8 | 16 | 32 | 64 | >64 |  |  |
| ERY |  | 3 | 0 | 0 | 1 | 36 | 32 | 1 | 0 | 0 | 0 | 2 | 0 | 23 | 1 | 25 (26%) |
| TEL | 3 | 0 | 0 | 0 | 1 | 7 | 43 | 22 | 3 | 1 | 18 |  |  |  | 1 | 19 (19%) |
| AZI | 3 | 4 | 36 | 28 | 2 | 0 | 0 | 0 | 0 | 0 | 0 | 0 | 0 | 25 | 0.12 | 25 (26%) |
| CLI |  | 3 | 1 | 22 | 41 | 8 | 0 | 8 | 11 | 3 | 0 | 1 |  |  | 0.25 | 23 (23%) |
| NAL |  |  |  |  |  |  |  |  | 45 | 32 | 0 | 0 | 5 | 16 | 8 | 21 (21%) |
| CIP | 3 | 1 | 22 | 43 | 8 | 0 | 0 | 0 | 0 | 7 | 14 | 0 | 0 | 0 | 0.12 | 21 (21%) |
| GEN |  |  |  | 3 | 1 | 40 | 54 | 0 | 0 | 0 | 0 | 0 | 0 |  | 1 | 0 (0%) |
| TET |  |  | 3 | 0 | 0 | 3 | 1 | 0 | 3 | 13 | 19 | 1 | 23 | 32 | 64 | 91 (93%) |
| FFN |  | 3 | 0 | 0 | 0 | 18 | 76 | 1 | 0 | 0 | 0 | 0 | 0 | 0 | 1 | 0 (0%) |

**S6-T6 – Cohort F.** Conventional production. 98 isolates (all fecal)

| **Drug** | **Concentration (μg/ml)** | | | | | | | | | | | | | | **Median MIC** | **n_resistant_ (%)** |
| --- | --- | --- | --- | --- | --- | --- | --- | --- | --- | --- | --- | --- | --- | --- | --- | --- |
|  | 0.015 | 0.03 | 0.06 | 0.12 | 0.25 | 0.5 | 1 | 2 | 4 | 8 | 16 | 32 | 64 | >64 |  |  |
| ERY |  | 2 | 0 | 0 | 4 | 51 | 24 | 8 | 0 | 0 | 0 | 0 | 0 | 9 | 0.5 | 9 (9%) |
| TEL | 2 | 0 | 0 | 0 | 0 | 13 | 52 | 17 | 5 | 2 | 7 |  |  |  | 1 | 9 (9%) |
| AZI | 2 | 10 | 48 | 22 | 6 | 1 | 0 | 0 | 0 | 0 | 0 | 0 | 0 | 9 | 0.06 | 9 (9%) |
| CLI |  | 2 | 2 | 32 | 39 | 14 | 0 | 2 | 6 | 1 | 0 | 0 |  |  | 0.25 | 9 (9%) |
| NAL |  |  |  |  |  |  |  |  | 43 | 27 | 4 | 0 | 9 | 15 | 8 | 24 (24%) |
| CIP | 2 | 0 | 15 | 43 | 12 | 0 | 1 | 0 | 2 | 10 | 11 | 2 | 0 | 0 | 0.12 | 26 (27%) |
| GEN |  |  |  | 2 | 1 | 46 | 47 | 2 | 0 | 0 | 0 | 0 | 0 |  | 1 | 0 (0%) |
| TET |  |  | 2 | 0 | 0 | 0 | 0 | 0 | 1 | 7 | 14 | 20 | 24 | 30 | 64 | 96 (98%) |
| FFN |  | 2 | 0 | 1 | 0 | 17 | 71 | 7 | 0 | 0 | 0 | 0 | 0 | 0 | 1 | 0 (0%) |

**S6-T7 – Cohort G.** Conventional production. 81 isolates (all fecal)

| **Drug** | **Concentration (μg/ml)** | | | | | | | | | | | | | | **Median MIC** | **n_resistant_ (%)** |
| --- | --- | --- | --- | --- | --- | --- | --- | --- | --- | --- | --- | --- | --- | --- | --- | --- |
|  | 0.015 | 0.03 | 0.06 | 0.12 | 0.25 | 0.5 | 1 | 2 | 4 | 8 | 16 | 32 | 64 | >64 |  |  |
| ERY |  | 2 | 0 | 0 | 5 | 33 | 32 | 6 | 0 | 0 | 0 | 0 | 0 | 3 | 1 | 3 (4%) |
| TEL | 2 | 0 | 0 | 0 | 0 | 21 | 27 | 28 | 1 | 1 | 1 |  |  |  | 1 | 2 (2%) |
| AZI | 2 | 4 | 45 | 25 | 1 | 1 | 0 | 0 | 0 | 0 | 0 | 0 | 0 | 3 | 0.06 | 3 (4%) |
| CLI |  | 2 | 2 | 33 | 36 | 6 | 0 | 1 | 0 | 1 | 0 | 0 |  |  | 0.25 | 2 (2%) |
| NAL |  |  |  |  |  |  |  |  | 10 | 6 | 2 | 1 | 38 | 24 | 64 | 62 (77%) |
| CIP | 3 | 0 | 4 | 8 | 1 | 0 | 0 | 0 | 6 | 22 | 37 | 0 | 0 | 0 | 8 | 65 (80%) |
| GEN |  |  |  | 2 | 3 | 37 | 38 | 1 | 0 | 0 | 0 | 0 | 0 |  | 0.5 | 0 (0%) |
| TET |  |  | 2 | 0 | 0 | 0 | 0 | 1 | 4 | 7 | 8 | 17 | 21 | 21 | 64 | 78 (96%) |
| FFN |  | 2 | 0 | 0 | 0 | 19 | 60 | 0 | 0 | 0 | 0 | 0 | 0 | 0 | 1 | 0 (0%) |

**S6-T8 – Cohort I.** Conventional production. 81 isolates (60 fecal, 5 food or water, 16 environmental)

| **Drug** | **Concentration (μg/ml)** | | | | | | | | | | | | | | **Median MIC** | **n_resistant_ (%)** |
| --- | --- | --- | --- | --- | --- | --- | --- | --- | --- | --- | --- | --- | --- | --- | --- | --- |
|  | 0.015 | 0.03 | 0.06 | 0.12 | 0.25 | 0.5 | 1 | 2 | 4 | 8 | 16 | 32 | 64 | >64 |  |  |
| ERY |  | 1 | 0 | 0 | 0 | 5 | 30 | 12 | 0 | 0 | 0 | 0 | 0 | 33 | 2 | 33 (41%) |
| TEL | 1 | 0 | 0 | 0 | 0 | 1 | 18 | 26 | 6 | 15 | 14 |  |  |  | 2 | 29 (36%) |
| AZI | 2 | 0 | 20 | 21 | 4 | 0 | 0 | 0 | 0 | 0 | 0 | 0 | 1 | 33 | 0.12 | 34 (42%) |
| CLI |  | 2 | 0 | 3 | 28 | 15 | 0 | 16 | 9 | 6 | 1 | 1 |  |  | 0.5 | 33 (41%) |
| NAL |  |  |  |  |  |  |  |  | 43 | 36 | 1 | 0 | 0 | 1 | 4 | 1 (1%) |
| CIP | 1 | 0 | 16 | 53 | 11 | 0 | 0 | 0 | 0 | 0 | 0 | 0 | 0 | 0 | 0.12 | 0 (0%) |
| GEN |  |  |  | 1 | 1 | 41 | 38 | 0 | 0 | 0 | 0 | 0 | 0 |  | 0.5 | 0 (0%) |
| TET |  |  | 1 | 0 | 0 | 1 | 0 | 0 | 0 | 0 | 0 | 1 | 10 | 68 | > 64 | 79 (98%) |
| FFN |  | 1 | 0 | 0 | 1 | 19 | 58 | 2 | 0 | 0 | 0 | 0 | 0 | 0 | 1 | 0 (0%) |

**S6-T9 – Cohort J.** Conventional production. 50 isolates (45 fecal, 5 environmental)

| **Drug** | **Concentration (μg/ml)** | | | | | | | | | | | | | | **Median MIC** | **n_resistant_ (%)** |
| --- | --- | --- | --- | --- | --- | --- | --- | --- | --- | --- | --- | --- | --- | --- | --- | --- |
|  | 0.015 | 0.03 | 0.06 | 0.12 | 0.25 | 0.5 | 1 | 2 | 4 | 8 | 16 | 32 | 64 | >64 |  |  |
| ERY |  | 0 | 0 | 0 | 0 | 0 | 12 | 23 | 0 | 0 | 0 | 1 | 0 | 14 | 2 | 15 (30%) |
| TEL | 0 | 0 | 0 | 0 | 0 | 0 | 4 | 31 | 4 | 4 | 7 |  |  |  | 2 | 11 (22%) |
| AZI | 0 | 0 | 6 | 25 | 4 | 0 | 0 | 0 | 0 | 0 | 0 | 0 | 0 | 15 | 0.12 | 15 (30%) |
| CLI |  | 0 | 0 | 2 | 20 | 11 | 4 | 6 | 7 | 0 | 0 | 0 |  |  | 0.5 | 13 (26%) |
| NAL |  |  |  |  |  |  |  |  | 22 | 27 | 0 | 0 | 0 | 1 | 8 | 1 (2%) |
| CIP | 0 | 0 | 7 | 28 | 14 | 0 | 1 | 0 | 0 | 0 | 0 | 0 | 0 | 0 | 0.12 | 1 (2%) |
| GEN |  |  |  | 0 | 2 | 23 | 23 | 2 | 0 | 0 | 0 | 0 | 0 |  | 1 | 0 (0%) |
| TET |  |  | 0 | 0 | 0 | 0 | 0 | 0 | 0 | 0 | 0 | 0 | 5 | 45 | > 64 | 50 (100%) |
| FFN |  | 0 | 0 | 0 | 0 | 14 | 36 | 0 | 0 | 0 | 0 | 0 | 0 | 0 | 1 | 0 (0%) |

**S6-T10 – Cohort S.** Antibiotic-free production. 69 isolates (41 fecal, 10 food or water, 18 environmental)

| **Drug** | **Concentration (μg/ml)** | | | | | | | | | | | | | | **Median MIC** | **n_resistant_ (%)** |
| --- | --- | --- | --- | --- | --- | --- | --- | --- | --- | --- | --- | --- | --- | --- | --- | --- |
|  | 0.015 | 0.03 | 0.06 | 0.12 | 0.25 | 0.5 | 1 | 2 | 4 | 8 | 16 | 32 | 64 | >64 |  |  |
| ERY |  | 1 | 0 | 0 | 3 | 7 | 40 | 7 | 0 | 0 | 0 | 0 | 0 | 11 | 1 | 11 (16%) |
| TEL | 1 | 0 | 0 | 2 | 1 | 1 | 21 | 24 | 9 | 8 | 2 |  |  |  | 2 | 10 (14%) |
| AZI | 1 | 0 | 8 | 47 | 2 | 0 | 0 | 0 | 0 | 0 | 0 | 0 | 0 | 11 | 0.12 | 11 (16%) |
| CLI |  | 1 | 0 | 4 | 22 | 26 | 5 | 2 | 4 | 5 | 0 | 0 |  |  | 0.5 | 11 (16%) |
| NAL |  |  |  |  |  |  |  |  | 29 | 40 | 0 | 0 | 0 | 0 | 8 | 0 (0%) |
| CIP | 1 | 1 | 21 | 27 | 17 | 2 | 0 | 0 | 0 | 0 | 0 | 0 | 0 | 0 | 0.12 | 0 (0%) |
| GEN |  |  |  | 1 | 1 | 21 | 46 | 0 | 0 | 0 | 0 | 0 | 0 |  | 1 | 0 (0%) |
| TET |  |  | 1 | 1 | 17 | 0 | 0 | 0 | 1 | 14 | 20 | 1 | 0 | 14 | 16 | 50 (72%) |
| FFN |  | 1 | 0 | 0 | 0 | 10 | 56 | 2 | 0 | 0 | 0 | 0 | 0 | 0 | 1 | 0 (0%) |

**S6-T11 – Cohort T.** Antibiotic-free production. 83 isolates (67 fecal, 11 food or water, 5 environmental)

| **Drug** | **Concentration (μg/ml)** | | | | | | | | | | | | | | **Median MIC** | **n_resistant_ (%)** |
| --- | --- | --- | --- | --- | --- | --- | --- | --- | --- | --- | --- | --- | --- | --- | --- | --- |
|  | 0.015 | 0.03 | 0.06 | 0.12 | 0.25 | 0.5 | 1 | 2 | 4 | 8 | 16 | 32 | 64 | >64 |  |  |
| ERY |  | 1 | 0 | 2 | 1 | 10 | 18 | 0 | 0 | 0 | 0 | 0 | 1 | 50 | > 64 | 51 (61%) |
| TEL | 1 | 0 | 0 | 3 | 0 | 2 | 22 | 4 | 0 | 10 | 41 |  |  |  | 8 | 51 (61%) |
| AZI | 1 | 2 | 21 | 8 | 0 | 0 | 0 | 0 | 0 | 0 | 0 | 0 | 0 | 51 | > 64 | 51 (61%) |
| CLI |  | 1 | 2 | 18 | 7 | 4 | 1 | 25 | 9 | 14 | 2 | 0 |  |  | 2 | 50 (60%) |
| NAL |  |  |  |  |  |  |  |  | 59 | 23 | 0 | 0 | 1 | 0 | 4 | 1 (1%) |
| CIP | 1 | 6 | 48 | 23 | 4 | 0 | 0 | 0 | 0 | 1 | 0 | 0 | 0 | 0 | 0.06 | 1 (1%) |
| GEN |  |  |  | 1 | 13 | 53 | 16 | 0 | 0 | 0 | 0 | 0 | 0 |  | 0.5 | 0 (0%) |
| TET |  |  | 1 | 3 | 1 | 0 | 21 | 6 | 0 | 0 | 9 | 13 | 13 | 16 | 32 | 51 (61%) |
| FFN |  | 1 | 0 | 0 | 21 | 15 | 38 | 8 | 0 | 0 | 0 | 0 | 0 | 0 | 1 | 0 (0%) |

**S6-T12 – Cohort U.** Antibiotic-free production. 94 isolates (all fecal)

| **Drug** | **Concentration (μg/ml)** | | | | | | | | | | | | | | **Median MIC** | **n_resistant_ (%)** |
| --- | --- | --- | --- | --- | --- | --- | --- | --- | --- | --- | --- | --- | --- | --- | --- | --- |
|  | 0.015 | 0.03 | 0.06 | 0.12 | 0.25 | 0.5 | 1 | 2 | 4 | 8 | 16 | 32 | 64 | >64 |  |  |
| ERY |  | 0 | 0 | 0 | 0 | 12 | 19 | 0 | 0 | 0 | 0 | 0 | 3 | 60 | > 64 | 63 (67%) |
| TEL | 0 | 0 | 0 | 0 | 0 | 3 | 20 | 8 | 1 | 14 | 48 |  |  |  | 16 | 62 (66%) |
| AZI | 0 | 1 | 17 | 13 | 0 | 0 | 0 | 0 | 0 | 0 | 0 | 0 | 0 | 63 | > 64 | 63 (67%) |
| CLI |  | 0 | 0 | 13 | 12 | 6 | 4 | 31 | 8 | 14 | 5 | 1 |  |  | 2 | 59 (63%) |
| NAL |  |  |  |  |  |  |  |  | 59 | 31 | 0 | 0 | 4 | 0 | 4 | 4 (4%) |
| CIP | 0 | 6 | 48 | 27 | 9 | 0 | 0 | 0 | 0 | 1 | 3 | 0 | 0 | 0 | 0.06 | 4 (4%) |
| GEN |  |  |  | 2 | 19 | 47 | 26 | 0 | 0 | 0 | 0 | 0 | 0 |  | 0.5 | 0 (0%) |
| TET |  |  | 0 | 0 | 3 | 1 | 18 | 8 | 1 | 0 | 6 | 27 | 8 | 22 | 32 | 64 (68%) |
| FFN |  | 0 | 0 | 0 | 27 | 16 | 48 | 3 | 0 | 0 | 0 | 0 | 0 | 0 | 1 | 0 (0%) |

**S6-T13 – Cohort Y.** Antibiotic-free production. 73 isolates (59 fecal, 5 food or water, 9 environmental)

| **Drug** | **Concentration (μg/ml)** | | | | | | | | | | | | | | **Median MIC** | **n_resistant_ (%)** |
| --- | --- | --- | --- | --- | --- | --- | --- | --- | --- | --- | --- | --- | --- | --- | --- | --- |
|  | 0.015 | 0.03 | 0.06 | 0.12 | 0.25 | 0.5 | 1 | 2 | 4 | 8 | 16 | 32 | 64 | >64 |  |  |
| ERY |  | 1 | 0 | 0 | 2 | 4 | 32 | 27 | 1 | 0 | 0 | 0 | 0 | 6 | 1 | 6 (8%) |
| TEL | 1 | 0 | 0 | 0 | 2 | 4 | 16 | 37 | 8 | 5 | 0 |  |  |  | 2 | 5 (7%) |
| AZI | 1 | 0 | 13 | 44 | 8 | 1 | 0 | 0 | 0 | 0 | 0 | 0 | 0 | 6 | 0.12 | 6 (8%) |
| CLI |  | 1 | 0 | 17 | 21 | 28 | 0 | 0 | 2 | 4 | 0 | 0 |  |  | 0.25 | 6 (8%) |
| NAL |  |  |  |  |  |  |  |  | 34 | 39 | 0 | 0 | 0 | 0 | 8 | 0 (0%) |
| CIP | 1 | 0 | 12 | 51 | 9 | 0 | 0 | 0 | 0 | 0 | 0 | 0 | 0 | 0 | 0.12 | 0 (0%) |
| GEN |  |  |  | 1 | 2 | 22 | 47 | 1 | 0 | 0 | 0 | 0 | 0 |  | 1 | 0 (0%) |
| TET |  |  | 1 | 0 | 3 | 3 | 3 | 1 | 10 | 13 | 4 | 0 | 13 | 22 | 16 | 62 (85%) |
| FFN |  | 1 | 0 | 0 | 0 | 7 | 65 | 0 | 0 | 0 | 0 | 0 | 0 | 0 | 1 | 0 (0%) |

**S6-T14 – Cohort Z.** Antibiotic-free production. 41 isolates (38 fecal, 3 environmental)

| **Drug** | **Concentration (μg/ml)** | | | | | | | | | | | | | | **Median MIC** | **n_resistant_ (%)** |
| --- | --- | --- | --- | --- | --- | --- | --- | --- | --- | --- | --- | --- | --- | --- | --- | --- |
|  | 0.015 | 0.03 | 0.06 | 0.12 | 0.25 | 0.5 | 1 | 2 | 4 | 8 | 16 | 32 | 64 | >64 |  |  |
| ERY |  | 0 | 0 | 23 | 2 | 9 | 7 | 0 | 0 | 0 | 0 | 0 | 0 | 0 | 0.12 | 0 (0%) |
| TEL | 0 | 0 | 0 | 24 | 1 | 1 | 15 | 0 | 0 | 0 | 0 |  |  |  | 0.12 | 0 (0%) |
| AZI | 0 | 24 | 12 | 3 | 1 | 1 | 0 | 0 | 0 | 0 | 0 | 0 | 0 | 0 | 0.03 | 0 (0%) |
| CLI |  | 0 | 26 | 15 | 0 | 0 | 0 | 0 | 0 | 0 | 0 | 0 |  |  | 0.06 | 0 (0%) |
| NAL |  |  |  |  |  |  |  |  | 30 | 11 | 0 | 0 | 0 | 0 | 4 | 0 (0%) |
| CIP | 0 | 15 | 14 | 12 | 0 | 0 | 0 | 0 | 0 | 0 | 0 | 0 | 0 | 0 | 0.06 | 0 (0%) |
| GEN |  |  |  | 0 | 7 | 33 | 1 | 0 | 0 | 0 | 0 | 0 | 0 |  | 0.5 | 0 (0%) |
| TET |  |  | 0 | 3 | 22 | 0 | 0 | 0 | 0 | 0 | 0 | 4 | 11 | 1 | 0.25 | 16 (39%) |
| FFN |  | 0 | 0 | 0 | 0 | 33 | 8 | 0 | 0 | 0 | 0 | 0 | 0 | 0 | 0.5 | 0 (0%) |
