# Supplementary material for: Risk factors for fluoroquinolone- and macrolide-resistance among swine Campylobacter coli using multi-layered chain graphs
Source: PLoS Comput Biol. 2025 Aug 13;21(8):e1012797. doi: 10.1371/journal.pcbi.1012797 (PMC12373276; doi:10.1371/journal.pcbi.1012797)
Supplement: S1 Code — Zip file with the R code necessary to run the chain graphs. (ZIP) [file pcbi.1012797.s007.zip › AMR-Chain-Graph-Examples/Chaingraph-Fitting-v1_1.html]

AMR Chain Graphs


# AMR Chain Graphs

#### 2025-01-10

## Introduction

This markdown document provides the core code used to fit the models
for estimating phenotypic resistances for 9 antibiotics in 683 \(\_Campylobacter jejuni\) isolates collected
from swine in conventional production systems and 399 collected from
antibiotic free (ABF) systems. This code relies on the chain graph
learning algorithm available on JH Lin’s GitHub here.

### Clear Env & Load Packages

### Load Packages, Code

```
library(parallel) 
library(doParallel) #Parallel packages used to increase speed of bootstrapping process
```

```
## Loading required package: foreach
```

```
## Loading required package: iterators
```

```
{
  ORIG_DIR <- getwd()
  CG_SRC_DIR <- 'R/cg source'
  CG_SRC_INIT_FILE <- '_Funcs_l1ML.R'
  setwd(CG_SRC_DIR)
  source(CG_SRC_INIT_FILE)
  source('_Funcs_SSLasso.R')
  setwd(ORIG_DIR)
} #The code for fitting the chain graphs is not provided as a R package and must be loaded into the environment via running the R files '_Funcs_l1ML.R' and '_Funcs_SSLasso.R'. These files also call other files included in R/cg source.
```

```
## Loading required package: glmnet
```

```
## Loading required package: Matrix
```

```
## Loaded glmnet 4.1-8
```

```
## Loading required package: glasso
```

```
## Total number of workers = 9.
```

```
## Loading required package: huge
```

```
## Loading required package: flare
```

```
## Loading required package: lattice
```

```
## Loading required package: MASS
```

```
## Loading required package: igraph
```

```
## 
## Attaching package: 'igraph'
```

```
## The following objects are masked from 'package:stats':
## 
##     decompose, spectrum
```

```
## The following object is masked from 'package:base':
## 
##     union
```

```
## Registered S3 methods overwritten by 'flare':
##   method       from
##   print.roc    huge
##   print.select huge
##   print.sim    huge
##   plot.roc     huge
##   plot.select  huge
##   plot.sim     huge
```

```
## Loading required package: expm
```

```
## 
## Attaching package: 'expm'
```

```
## The following object is masked from 'package:Matrix':
## 
##     expm
```

```
## Loading required package: gdata
```

```
## 
## Attaching package: 'gdata'
```

```
## The following object is masked from 'package:stats':
## 
##     nobs
```

```
## The following object is masked from 'package:utils':
## 
##     object.size
```

```
## The following object is masked from 'package:base':
## 
##     startsWith
```

```
source('R/Function chaingraph R2 estimates v4.R')
source("R/FUNCTION chain displays.R")
source("R/FUNCTION_cg.data.R")
source("R/FUNCTION bootstrap estimation of CG CIs.R")
source("R/FUNCTION estimate partial correlations.R")
#These files facilitate the calling and reporting of results from the chain graph functions.
```

### Create Center Function

`center` is a simple function for centering a vector of
values around the vector mean.

```
center <- function(x) return(x - mean(x))
```

### Import Data

#### Data Sets

The data objects `conv` and `abf` contain the
data from the conventional and antibiotic free farms respectively. The
first 9 columns contain the \(log\_2\)
MIC results and produce the respective \(Y\) matrix:

- AZIlog: \(log\_2\) Azithromycin
  MICs
- CIPlog: \(log\_2\) Ciprofloxacin
  MICs
- ERYlog: \(log\_2\) Erythromycin
  MICs
- GENlog: \(log\_2\) Gentamicin
  MICs
- TETlog: \(log\_2\) Tetracycline
  MICs
- FFNlog: \(log\_2\) Florfenicol
  MICs
- NALlog: \(log\_2\) Nalidixic Acid
  MICs
- TELlog: \(log\_2\) Telithromycin
  MICs
- CLIlog: \(log\_2\) Clindamycin
  MICs

Matrix \(X\) is formed from the
genetic variables in columns 10 through 20:

- X23S\_A2075G: a 23S rRNA mutation associated with telithromycin
  resistance
- X50S\_L22\_A103V: a 50S L22 protein mutation associated with macrolide
  resistance
- aadE\_Cc: a 6’ adenyltransferase gene associated with aminoglycoside
  resistance
- acr3: an arsenite permease gene associated with increased resistance
  to arsenic compounds
- aph.3pr\_IIIa: a plasmid-encoded phosphotransferase associated with
  aminoglycoside resistance
- blaOXA\_193: a beta-lactamase gene associated with resistance to
  penicillins and cephalosporins
- blaOXA\_578: a beta-lactamase gene associated with resistance to
  penicillins and cephalosporins
- gyrA\_T86I: a gyrase A mutation associated with resistance for
  fluoroquinolones and nalidixic acid
- rpsL\_K43R: a 30S rRNA subunit mutation associated with streptomycin
  resistance
- tet.O: a ribosomal protection gene associated with tetracycline
  resistance.

These variables are coded 1 for the presence of the gene or mutation,
and 0 for the abscence of the gene or wild type.

Matrix \(W\) is formed from the
environmental variables. The specific variables in the matrices vary by
production system.

- Disinfectant.QuatAmmonium (Both systems): quartanary ammonium
  disinfectant used on the farm
- Obs.GI.signs (Both systems): evidence of gastrointestinal
  abnormalities observed in swine during researchers visit
- Ruminants (Both systems): ruminants kept on the farm premises
- SampleStage.F1 (Both systems): Sample from early finishing
  (referent: farrowing)
- SampleStage.F2 (Both systems): Sample from late finishing (referent:
  farrowing)
- SampleStage.N1 (Both systems): Sample from early nursery (referent:
  farrowing)
- SampleStage.N2 (Both systems): sample from late nursery (referent:
  farrowing)
- Source.Fecal.Sow (Both systems): sample collected from sow
  feces
- Source.Swab.Surface (Both systems): sample collected from building
  surface
- Source.Water (Both systems): sample collected from hogs’ drinking
  water
- Birds (ABF only): Presence or access of birds to hogs
- Chicks.Ducks (ABF only): presence or access of chickens or ducks
  hogs
- CuExp (ABF only): Hogs received copper supplements
- Disinfectant.Clorox (ABF only): Chlorox used as a disinfectant on
  premises
- FeedSource.Farm (ABF only): Feed milled/mixed on farm (1) or
  elsewhere (0)
- Obs.Toilet (ABF only): Toilet on premises
- Source.Soil (ABF only): Sample collected from soil (referent?)
- Visitors.Day (ABF only): Number of visitors per day, including
  workers
- WaterQC (ABF only): water control program for premises
- ZnExp (ABF only): Hogs received Zinc supplements
- Pig.Mixing (Conventional only): Weaned hogs from different litters
  comingled
- Enter.Hrs (Conventional only): Number of hours required to enter
  after visiting other hog production premises
- Rodents (Conventional only): Were rodents noted at researcher
  visit
- Insects (Conventional only): Were insects noted at researcher
  visit
- Visitors.Wk (Conventional only): number of visitors per week,
  including workers
- PressureWash (Conventional only): Were pens pressure washed between
  litters
- Obs.Entry.Measures (Conventional only): Measures required prior to
  entering premises
- Obs.Shower (Conventional only): Shower-in before entrance
- Obs.Resp.signs (Conventional only): hogs have visible clinical signs
  of respiratory disease
- Carbadox.dum (Conventional only): Carbadox used on site, any
  dose
- Tiamulin.dum (Conventional only): Tiamulin used on site, any
  dose
- CTC.dum (Conventional only): Chlortetracycline used on site, any
  dose
- Lincomycin.dum (Conventional only): Lincomycin used on site, any
  dose
- OTC.dum (Conventional only): Oxytetracycline used on site, any
  dose
- Roxarsone.dum (Conventional only): Roxarsone used on site, any
  dose
- Virginiamycin.dum (Conventional only): Virginamycin used on site,
  any dose
- Pen.G.dum (Conventional only): Penicillin used on site, any
  dose
- Enrofloxacin.dum (Conventional only): Enrofloxacin used on site, any
  dose
- Ceftiofur.dum (Conventional only): Ceftiofur used on site, any
  dose
- Site.Flow.Batch (Conventional only): Hogs moved as batches across
  entire site
- Barn.Flow.Batch (Conventional only): Hogs moved as batches by
  barn
- Room.Flow.Batch (Conventional only): Hogs moved as batches by
  room
- LabConf.Resp (Conventional only): lab tests used to confirm
  respiratory pathogens
- Source.Feed (Conventional only): Sample collected from feed
- Source.Lagoon (Conventional only): Sample collected from farm
  lagoon

```
#conv <- read.csv("data/campy-conv-cg-data_2021.10.21.csv", header = T)[,-c(1)]
#abf <- read.csv("data/campy-abf-cg-data_2021.10.21.csv", header = T)[,-c(1)]
#abf.leaner <- read.csv("data/campy-abf-cg-data_2022.02.15.csv", header = T)[,-c(1)]

#conv <- conv[,-c(1:9,19,31,33,34,59:63,74:76)]

load('data/campy-cg-data.rda')

conv.y <- as.matrix(conv[,c(1:9)])
conv.gen <- data.matrix(conv[,c(10:20)])
conv.env <- as.matrix(conv[,c(21:55)])
conv.env_gen <- as.matrix(conv[,c(10:55)])


#abf.lean <- abf[,-c(1:9,19,33:37,58:60)]

abf.y <- as.matrix(abf[,c(1:9)])
abf.gen <- data.matrix(abf[,c(10:22)])
abf.env <- as.matrix(abf[,c(23:42)])
abf.env_gen <- as.matrix(abf[,c(10:42)])
```

#### Center Data Sets

The data matrix columns are centered using the `center`
function defined above. Centering data in this way is suggested in these
chain graphs to avoid the need to fit an intercept in the linear
models.

```
conv.env_cent <- apply(conv.env, 2, center)
conv.gen_cent <- apply(conv.gen, 2, center)
conv.env_gen_cent <- apply(conv.env_gen, 2, center)
conv.y_cent <- apply(conv.y, 2, center)

abf.env_cent <- apply(abf.env, 2, center)
abf.gen_cent <- apply(abf.gen, 2, center)
abf.env_gen_cent <- apply(abf.env_gen, 2, center)
abf.y_cent <- apply(abf.y, 2, center)
```

### Bootstrapping

Bootstrapping is used to approximate the confidence intervals for the
partial correlations.

### Define Bootstrap Criteria for CIs

```
B <- 200 #B bootstraps will be fit

seeds <- 1:6 #Six sets of bootstraps are performed, each using a different static seed, 1 through 6.

n_b_conv <- round(0.5 * dim(conv.y)[1])
n_b_abf <- round(0.5 * dim(abf.y)[1])
#sets the bootstrap sample size to to 50% of the total sample in conventional and abf data sets.
```

#### Select Number of Cores for doParallel

```
RESERVED_CORES = 2 #Reserves a number of logical cores for the system to use while bootstraps are calculated in parallel

n_cl <- parallel::detectCores() - RESERVED_CORES #Determines the number of cores to use for parallel bootstrapping
```

## Fitting Chain Graphs

Six chain graps are fit, one for each combination of the two
productions systems and three predictor sets (\(X\): Environmental predictors, \(W\): Genetic predictors, \(WX\): Environmental and genetic predictors
combined). Comments explaining the code are provided in the first code
set (Conventional production + environmental predictors) only since code
is duplicated in later iterations.

### Conventional Farms

```
Y <- conv.y_cent
```

#### Environmental

```
X <- conv.env_cent

model.cg = l1ML_Main(
  Y = Y, 
  X = X, 
  initializer = 'Lasso', 
  screening = T,
  ss = T,
  alpha = 0.05,
  nboot = 20
)
```

```
## Step 0: screening is on, proceed with debiased Lasso in conjunction with BH correction, the cut-off for p-values is set at 0.05 .
## Step 1: Penalized LS initialization with Lasso .
## Step 2: Alternate update.
```

```
## Warning in l1ML_Main(Y = Y, X = X, initializer = "Lasso", screening = T, : l1ML_Main(): alternate update is broken manually at iteration =50
```

```
## Step 3: Refitting B Matrix.
## Step 4: Stability selection with a total number of 20 bootstrapped samples ...25%...50%...75%...Done!
## Step 5: Refitting with weighted glasso ...Done.
## Returns: B.est, Theta.est, BICvalue.
```

```
#The l1ML_Main function is the top-level function available from JH Lin's GitHub

model.cg_formatted <- format.chain.graph(model.cg, x.names = colnames(X), y.names = colnames(Y))
#format.chain.graph() adds names and to output data matrices and 

print(display.chain.graph(model.cg_formatted))
```

```
## 
## Lambda: 
## Edges: 119 (38%)
## 
## Beta Matrix:
##                            AZIlog CIPlog ERYlog GENlog TETlog FFNlog NALlog
## Pig.Mixing                 0.637  0.000  0.331 -0.090  0.000  0.000  0.000
## Enter.Hrs                  0.000  0.000  0.000  0.000  0.000  0.000  0.000
## Rodents                    0.000  0.000  0.000 -0.215  0.000 -0.118  0.000
## Ruminants                  2.166 -1.172  1.511  0.000  0.871  0.000 -0.150
## Insects                   -0.908  0.959 -0.235  0.000 -0.389  0.000  0.125
## Visitors.Wk                0.000  0.000  0.000  0.000  0.000  0.000  0.000
## Disinfectant.QuatAmmonium  0.473 -0.226  0.461 -0.122  0.146  0.000  0.000
## PressureWash               0.000  0.000 -0.303  0.000 -1.212  0.000  0.000
## Obs.Entry.Measures         0.000  2.116  0.000  0.000 -0.824  0.000  0.974
## Obs.Shower                -0.824  0.000 -0.643  0.000 -0.493  0.000  0.000
## Obs.GI.signs               0.000 -0.821  0.000  0.000 -0.272  0.000 -0.191
## Obs.Resp.signs             2.736  0.000  1.612  0.000  0.267 -0.206 -0.336
## Carbadox.dum               0.000  0.000  0.000  0.000  0.000  0.000  0.000
## Tiamulin.dum              -1.894  0.056 -0.914  0.000  0.000  0.000  0.080
## CTC.dum                    0.000  0.694  0.000 -0.050  0.000 -0.093  0.000
## Lincomycin.dum             0.000  1.311  0.000  0.000  1.699  0.000  0.515
## OTC.dum                    0.958  0.000  0.000  0.000  0.000  0.000  0.000
## Roxarsone.dum              2.401  0.000  1.262 -0.142  0.000  0.000  0.000
## Virginiamycin.dum          0.000  0.000  0.000  0.000  0.000  0.000  0.000
## Pen.G.dum                  0.000 -2.462  0.000  0.000  0.000  0.000 -1.022
## Enrofloxacin.dum           0.000  0.000  0.000  0.000 -1.256  0.000  0.000
## Ceftiofur.dum              0.000 -1.891  0.000  0.000  0.000  0.000 -0.593
## Site.Flow.Batch            0.000 -1.801  0.000  0.000  0.000  0.000 -1.191
## Barn.Flow.Batch            0.779  0.206  0.313  0.000 -0.152  0.000  0.354
## Room.Flow.Batch            0.000  0.000  0.000  0.000  0.000  0.000  0.000
## LabConf.Resp              -1.685  1.982 -0.699  0.000  0.000  0.000  1.127
## Source.Feed               -1.007 -0.131 -0.556  0.000  0.000  0.000  0.000
## Source.Water              -0.873 -0.218 -0.809  0.000  0.238  0.173 -0.208
## Source.Lagoon             -1.369 -0.074 -0.839  0.000  0.000  0.000 -0.142
## Source.Fecal.Sow          -1.115  0.000 -0.575  0.213  0.355  0.000  0.000
## Source.Swab.Surface       -0.987  0.000 -0.639  0.000  0.000  0.178  0.000
## SampleStage.N1             0.000  0.000  0.000  0.000  0.000  0.000  0.175
## SampleStage.N2            -0.452  0.000  0.000 -0.143  0.516  0.000  0.000
## SampleStage.F1             0.000  0.000  0.000  0.000  0.424  0.000  0.000
## SampleStage.F2             0.000  0.000  0.000 -0.231 -0.026 -0.259  0.000
##                           TELlog CLIlog
## Pig.Mixing                 0.000  0.000
## Enter.Hrs                  0.000  0.000
## Rodents                    0.484  0.000
## Ruminants                  0.336  0.132
## Insects                   -0.195  0.069
## Visitors.Wk                0.000  0.000
## Disinfectant.QuatAmmonium -0.088  0.151
## PressureWash               0.000 -0.125
## Obs.Entry.Measures         0.000  0.109
## Obs.Shower                -0.410 -0.094
## Obs.GI.signs               0.000  0.000
## Obs.Resp.signs             0.268  1.033
## Carbadox.dum               0.000  0.000
## Tiamulin.dum              -0.548 -0.526
## CTC.dum                    0.000  0.000
## Lincomycin.dum             0.000  0.000
## OTC.dum                    0.000  0.000
## Roxarsone.dum              0.000  0.184
## Virginiamycin.dum          0.000  0.000
## Pen.G.dum                  0.000  0.000
## Enrofloxacin.dum           0.000  0.000
## Ceftiofur.dum              0.213  0.000
## Site.Flow.Batch            0.000  0.000
## Barn.Flow.Batch            0.000  0.000
## Room.Flow.Batch            0.000  0.000
## LabConf.Resp              -0.125 -0.276
## Source.Feed                0.000 -0.146
## Source.Water               0.000 -0.107
## Source.Lagoon              0.000 -0.364
## Source.Fecal.Sow          -0.075 -0.106
## Source.Swab.Surface        0.000  0.000
## SampleStage.N1             0.000  0.000
## SampleStage.N2            -0.053  0.000
## SampleStage.F1             0.000  0.000
## SampleStage.F2             0.000  0.000
## 
## 
## Rho: 
## Edges: 26 (72%)
## 
## Omega Matrix: 
##        AZIlog CIPlog ERYlog GENlog TETlog FFNlog NALlog TELlog CLIlog
## AZIlog  0.000 -0.014  0.846 -0.077  0.000 -0.227 -0.008 -0.186  0.089
## CIPlog -0.014  0.000  0.000  0.000  0.028  0.139  0.912  0.044  0.000
## ERYlog  0.847  0.000  0.000  0.000  0.012  0.000  0.000  0.465  0.200
## GENlog -0.077  0.000  0.000  0.000  0.141  0.275  0.024  0.045  0.125
## TETlog  0.000  0.028  0.012  0.141  0.000  0.340  0.000  0.261 -0.214
## FFNlog -0.227  0.139  0.000  0.275  0.340  0.000 -0.129  0.362  0.000
## NALlog -0.008  0.912  0.000  0.024  0.000 -0.129  0.000  0.000 -0.031
## TELlog -0.186  0.044  0.464  0.045  0.261  0.362  0.000  0.000  0.324
## CLIlog  0.089  0.000  0.200  0.125 -0.214  0.000 -0.031  0.325  0.000
## 
## 
## NULL
```

```
R2.tab <- ChainGraph_R2_v4(
  X, 
  Y, 
  Beta = model.cg_formatted$B.est, 
  Omega = model.cg_formatted$Omega.est
)
#ChainGraph_R2_v4() uses the models selected in model.cg to estimate the R2 values for directed and undirected portions of the chain graph for each variable in Y

print(R2.tab)
```

```
##          Y
## AZIlog AZI
## CIPlog CIP
## ERYlog ERY
## GENlog GEN
## TETlog TET
## FFNlog FFN
## NALlog NAL
## TELlog TEL
## CLIlog CLI
##                                                                                                                                                                                                                                                               X_set
## AZIlog  Pig.Mixing, Ruminants, Insects, Disinfectant.QuatAmmonium, Obs.Shower, Obs.Resp.signs, Tiamulin.dum, OTC.dum, Roxarsone.dum, Barn.Flow.Batch, LabConf.Resp, Source.Feed, Source.Water, Source.Lagoon, Source.Fecal.Sow, Source.Swab.Surface, SampleStage.N2
## CIPlog                   Ruminants, Insects, Disinfectant.QuatAmmonium, Obs.Entry.Measures, Obs.GI.signs, Tiamulin.dum, CTC.dum, Lincomycin.dum, Pen.G.dum, Ceftiofur.dum, Site.Flow.Batch, Barn.Flow.Batch, LabConf.Resp, Source.Feed, Source.Water, Source.Lagoon
## ERYlog             Pig.Mixing, Ruminants, Insects, Disinfectant.QuatAmmonium, PressureWash, Obs.Shower, Obs.Resp.signs, Tiamulin.dum, Roxarsone.dum, Barn.Flow.Batch, LabConf.Resp, Source.Feed, Source.Water, Source.Lagoon, Source.Fecal.Sow, Source.Swab.Surface
## GENlog                                                                                                                                     Pig.Mixing, Rodents, Disinfectant.QuatAmmonium, CTC.dum, Roxarsone.dum, Source.Fecal.Sow, SampleStage.N2, SampleStage.F2
## TETlog Ruminants, Insects, Disinfectant.QuatAmmonium, PressureWash, Obs.Entry.Measures, Obs.Shower, Obs.GI.signs, Obs.Resp.signs, Lincomycin.dum, Enrofloxacin.dum, Barn.Flow.Batch, Source.Water, Source.Fecal.Sow, SampleStage.N2, SampleStage.F1, SampleStage.F2
## FFNlog                                                                                                                                                                          Rodents, Obs.Resp.signs, CTC.dum, Source.Water, Source.Swab.Surface, SampleStage.F2
## NALlog                                    Ruminants, Insects, Obs.Entry.Measures, Obs.GI.signs, Obs.Resp.signs, Tiamulin.dum, Lincomycin.dum, Pen.G.dum, Ceftiofur.dum, Site.Flow.Batch, Barn.Flow.Batch, LabConf.Resp, Source.Water, Source.Lagoon, SampleStage.N1
## TELlog                                                                                              Rodents, Ruminants, Insects, Disinfectant.QuatAmmonium, Obs.Shower, Obs.Resp.signs, Tiamulin.dum, Ceftiofur.dum, LabConf.Resp, Source.Fecal.Sow, SampleStage.N2
## CLIlog                                           Ruminants, Insects, Disinfectant.QuatAmmonium, PressureWash, Obs.Entry.Measures, Obs.Shower, Obs.Resp.signs, Tiamulin.dum, Roxarsone.dum, LabConf.Resp, Source.Feed, Source.Water, Source.Lagoon, Source.Fecal.Sow
##              R2_X                             Y_set   R2_e_Yj     R2_Yj
## AZIlog 0.15907495 CIP, ERY, GEN, FFN, NAL, TEL, CLI 0.9604589 0.8076740
## CIPlog 0.37523872           AZI, TET, FFN, NAL, TEL 0.8566358 0.5351929
## ERYlog 0.15287171                AZI, TET, TEL, CLI 0.9733070 0.8245159
## GENlog 0.05696755      AZI, TET, FFN, NAL, TEL, CLI 0.3902916 0.3680576
## TETlog 0.18027179      CIP, ERY, GEN, FFN, TEL, CLI 0.5469669 0.4483642
## FFNlog 0.02498098      AZI, CIP, GEN, TET, NAL, TEL 0.6592950 0.6428252
## NALlog 0.31988053           AZI, CIP, GEN, FFN, CLI 0.8478236 0.5766214
## TELlog 0.08212369 AZI, CIP, ERY, GEN, TET, FFN, CLI 0.9039150 0.8296822
## CLIlog 0.10130207      AZI, ERY, GEN, TET, NAL, TEL 0.8701123 0.7819681
##         R2_total
## AZIlog 0.9667489
## CIPlog 0.9104316
## ERYlog 0.9773876
## GENlog 0.4250252
## TETlog 0.6286360
## FFNlog 0.6678062
## NALlog 0.8965019
## TELlog 0.9118059
## CLIlog 0.8832702
```

##### Bootstrap

```
cl <- parallel::makeCluster(n_cl)
doParallel::registerDoParallel(cl)

model.cg_bs <- cg.bootstrap(
  Y = Y, 
  X = X, 
  b = B,
  n_b = n_b_conv,
  lambda = model.cg$lambda, 
  rho = model.cg$rho,
  initializer = 'Lasso', 
  screening = T,
  alpha = 0.05,
  nboot = 20,
  cluster = cl,
  seed.val = seeds[1]
)
```

```
## Starting parallel bootstrap at 2025-01-10 21:16:45.985432
```

```
## Ending bootstrap at 2025-01-10 21:28:04.000258
```

```
#stopCluster(cl)

summary.cg_bootstrap_results(model.cg_bs) #shows bootstrap results with a seperate row for each edge in the graph.
```

```
## Warning in summary.cg_bootstrap_results(model.cg_bs): Target number of
## subsamples (b = 200) exceeds available valid subsamples (b = 198).
```

```
## SAMPLE SIZES
## Original:  683 
## Subsample size:  342 
## 
## BOOTSTRAPS
## Total fit subsamples:  200 
## Subsamples for summary stats: 198 
## Subsamples that returned NAs: 2 
## 
## PENALTIES
## Lambda:  0.07214907 
## Rho:  0.05671878 
## 
## CI alpha: 95 %
## 
## BETA EDGES:
##                             X      Y n_non0  B_bar B_bar_non0  SE_B SE_non0
## 1                  Pig.Mixing AZIlog    143  0.652      0.903 0.510   0.364
## 2                     Rodents AZIlog    105  0.243      0.458 0.656   0.846
## 3                   Ruminants AZIlog    191  2.455      2.545 1.075   0.984
## 4                     Insects AZIlog    133 -0.624     -0.929 0.539   0.386
## 5   Disinfectant.QuatAmmonium AZIlog     89  0.403      0.896 0.596   0.590
## 6                PressureWash AZIlog     44 -0.192     -0.866 0.426   0.485
## 7          Obs.Entry.Measures AZIlog     52 -0.116     -0.440 0.248   0.304
## 8                  Obs.Shower AZIlog    147 -0.802     -1.080 0.651   0.518
## 9                Obs.GI.signs AZIlog     37 -0.115     -0.613 0.285   0.361
## 10             Obs.Resp.signs AZIlog    197  2.831      2.846 1.183   1.169
## 11               Tiamulin.dum AZIlog    181 -1.220     -1.334 0.632   0.533
## 12             Lincomycin.dum AZIlog     49  0.145      0.586 0.458   0.773
## 13                    OTC.dum AZIlog     92  0.203      0.438 0.448   0.575
## 14              Roxarsone.dum AZIlog    182  1.796      1.954 0.939   0.806
## 15          Virginiamycin.dum AZIlog      5 -0.028     -1.128 0.190   0.479
## 16                  Pen.G.dum AZIlog      5  0.023      0.923 0.153   0.338
## 17           Enrofloxacin.dum AZIlog      3 -0.006     -0.390 0.070   0.504
## 18              Ceftiofur.dum AZIlog     24  0.008      0.066 0.272   0.794
## 19            Site.Flow.Batch AZIlog     15  0.013      0.178 0.190   0.688
## 20            Barn.Flow.Batch AZIlog    117  0.482      0.816 0.467   0.309
## 21            Room.Flow.Batch AZIlog     72 -0.232     -0.639 0.415   0.462
## 22               LabConf.Resp AZIlog    184 -1.454     -1.564 0.642   0.520
## 23                Source.Feed AZIlog    142 -1.044     -1.456 1.040   0.952
## 24               Source.Water AZIlog    132 -0.809     -1.213 0.661   0.403
## 25              Source.Lagoon AZIlog    196 -1.463     -1.478 0.473   0.451
## 26           Source.Fecal.Sow AZIlog    153 -0.930     -1.204 0.647   0.460
## 27        Source.Swab.Surface AZIlog    121 -0.801     -1.310 0.728   0.444
## 28             SampleStage.N1 AZIlog     33  0.165      0.991 0.399   0.371
## 29             SampleStage.N2 AZIlog     83 -0.182     -0.435 0.342   0.413
## 30             SampleStage.F1 AZIlog     78  0.181      0.460 0.305   0.328
## 31             SampleStage.F2 AZIlog     10 -0.023     -0.451 0.239   1.016
## 32                 Pig.Mixing CIPlog    120 -0.240     -0.396 0.300   0.294
## 33                    Rodents CIPlog     59  0.070      0.234 0.371   0.655
## 34                  Ruminants CIPlog    127 -0.671     -1.046 0.622   0.457
## 35                    Insects CIPlog    167  0.777      0.921 0.443   0.315
## 36                Visitors.Wk CIPlog      1 -0.001     -0.119 0.008   0.000
## 37  Disinfectant.QuatAmmonium CIPlog     28 -0.048     -0.341 0.159   0.284
## 38               PressureWash CIPlog     58  0.063      0.215 0.167   0.252
## 39         Obs.Entry.Measures CIPlog    198  2.101      2.101 0.318   0.318
## 40                 Obs.Shower CIPlog      2  0.001      0.146 0.016   0.101
## 41               Obs.GI.signs CIPlog    131 -0.517     -0.782 0.439   0.288
## 42             Obs.Resp.signs CIPlog     15  0.035      0.460 0.153   0.345
## 43               Carbadox.dum CIPlog      7  0.014      0.389 0.092   0.327
## 44               Tiamulin.dum CIPlog    130  0.300      0.458 0.322   0.293
## 45                    CTC.dum CIPlog     25  0.077      0.608 0.219   0.239
## 46             Lincomycin.dum CIPlog    186  1.076      1.145 0.709   0.675
## 47                    OTC.dum CIPlog     35 -0.023     -0.133 0.183   0.424
## 48          Virginiamycin.dum CIPlog     17  0.069      0.802 0.253   0.408
## 49                  Pen.G.dum CIPlog    198 -2.367     -2.367 0.348   0.348
## 50              Ceftiofur.dum CIPlog    198 -1.880     -1.880 0.340   0.340
## 51            Site.Flow.Batch CIPlog    198 -1.904     -1.904 0.391   0.391
## 52            Barn.Flow.Batch CIPlog     78  0.192      0.486 0.317   0.334
## 53            Room.Flow.Batch CIPlog      7 -0.050     -1.428 0.273   0.389
## 54               LabConf.Resp CIPlog    198  2.219      2.219 0.261   0.261
## 55                Source.Feed CIPlog    106 -0.197     -0.368 0.231   0.191
## 56               Source.Water CIPlog     96 -0.180     -0.372 0.231   0.197
## 57              Source.Lagoon CIPlog     82 -0.121     -0.291 0.216   0.252
## 58           Source.Fecal.Sow CIPlog     14 -0.013     -0.185 0.116   0.410
## 59        Source.Swab.Surface CIPlog     43  0.023      0.106 0.193   0.408
## 60             SampleStage.N1 CIPlog     65  0.113      0.343 0.201   0.209
## 61             SampleStage.N2 CIPlog     25  0.024      0.190 0.071   0.090
## 62             SampleStage.F1 CIPlog     27 -0.007     -0.048 0.133   0.363
## 63             SampleStage.F2 CIPlog     16  0.060      0.739 0.220   0.321
## 64                 Pig.Mixing ERYlog    118  0.306      0.513 0.314   0.244
## 65                    Rodents ERYlog     86  0.101      0.232 0.341   0.489
## 66                  Ruminants ERYlog    192  1.592      1.642 0.684   0.633
## 67                    Insects ERYlog    123 -0.218     -0.351 0.257   0.244
## 68  Disinfectant.QuatAmmonium ERYlog     91  0.266      0.578 0.384   0.375
## 69               PressureWash ERYlog     79 -0.242     -0.605 0.376   0.365
## 70         Obs.Entry.Measures ERYlog     73 -0.091     -0.246 0.160   0.176
## 71                 Obs.Shower ERYlog    169 -0.616     -0.722 0.425   0.368
## 72               Obs.GI.signs ERYlog     71 -0.108     -0.302 0.188   0.202
## 73             Obs.Resp.signs ERYlog    192  1.736      1.790 0.815   0.767
## 74               Tiamulin.dum ERYlog    190 -0.783     -0.816 0.406   0.380
## 75             Lincomycin.dum ERYlog     45  0.104      0.459 0.305   0.501
## 76                    OTC.dum ERYlog     76 -0.073     -0.190 0.188   0.266
## 77              Roxarsone.dum ERYlog    190  1.222      1.274 0.583   0.537
## 78          Virginiamycin.dum ERYlog      8 -0.012     -0.302 0.080   0.284
## 79                  Pen.G.dum ERYlog      1  0.001      0.150 0.011   0.000
## 80           Enrofloxacin.dum ERYlog     26 -0.050     -0.380 0.139   0.145
## 81              Ceftiofur.dum ERYlog     36  0.051      0.280 0.179   0.337
## 82            Site.Flow.Batch ERYlog     15  0.012      0.157 0.090   0.299
## 83            Barn.Flow.Batch ERYlog     78  0.194      0.492 0.257   0.140
## 84            Room.Flow.Batch ERYlog     55 -0.064     -0.230 0.171   0.261
## 85               LabConf.Resp ERYlog    174 -0.661     -0.752 0.385   0.316
## 86                Source.Feed ERYlog    132 -0.531     -0.797 0.571   0.527
## 87               Source.Water ERYlog    173 -0.741     -0.848 0.376   0.265
## 88              Source.Lagoon ERYlog    194 -0.845     -0.863 0.302   0.279
## 89           Source.Fecal.Sow ERYlog    141 -0.504     -0.708 0.402   0.287
## 90        Source.Swab.Surface ERYlog    111 -0.457     -0.815 0.447   0.251
## 91             SampleStage.N1 ERYlog     25  0.057      0.454 0.166   0.197
## 92             SampleStage.N2 ERYlog     44 -0.059     -0.263 0.128   0.143
## 93             SampleStage.F1 ERYlog     63  0.077      0.243 0.142   0.152
## 94             SampleStage.F2 ERYlog     11 -0.009     -0.157 0.097   0.400
## 95                 Pig.Mixing GENlog     66 -0.047     -0.142 0.076   0.064
## 96                    Rodents GENlog    154 -0.206     -0.265 0.146   0.109
## 97                  Ruminants GENlog     40  0.046      0.229 0.096   0.064
## 98                    Insects GENlog     43 -0.033     -0.151 0.068   0.056
## 99  Disinfectant.QuatAmmonium GENlog     95 -0.100     -0.209 0.131   0.114
## 100              PressureWash GENlog      5  0.005      0.187 0.030   0.046
## 101        Obs.Entry.Measures GENlog     12  0.008      0.125 0.034   0.071
## 102                Obs.Shower GENlog     27 -0.017     -0.124 0.047   0.057
## 103              Obs.GI.signs GENlog      4  0.000      0.019 0.024   0.193
## 104            Obs.Resp.signs GENlog     13  0.003      0.051 0.044   0.172
## 105              Carbadox.dum GENlog      6 -0.001     -0.031 0.020   0.123
## 106              Tiamulin.dum GENlog     19 -0.006     -0.067 0.030   0.077
## 107                   CTC.dum GENlog    147 -0.094     -0.126 0.085   0.074
## 108            Lincomycin.dum GENlog     43 -0.055     -0.254 0.120   0.124
## 109             Roxarsone.dum GENlog    108 -0.107     -0.196 0.123   0.102
## 110         Virginiamycin.dum GENlog     17 -0.013     -0.153 0.047   0.067
## 111          Enrofloxacin.dum GENlog      9 -0.009     -0.197 0.046   0.102
## 112             Ceftiofur.dum GENlog     15 -0.009     -0.113 0.057   0.181
## 113           Site.Flow.Batch GENlog     10 -0.007     -0.146 0.051   0.184
## 114           Barn.Flow.Batch GENlog     79 -0.072     -0.180 0.100   0.075
## 115           Room.Flow.Batch GENlog     61  0.061      0.199 0.112   0.117
## 116              LabConf.Resp GENlog     29 -0.021     -0.146 0.055   0.052
## 117               Source.Feed GENlog     34 -0.049     -0.283 0.131   0.186
## 118              Source.Water GENlog     20  0.015      0.151 0.062   0.134
## 119             Source.Lagoon GENlog     46  0.045      0.193 0.085   0.049
## 120          Source.Fecal.Sow GENlog    151  0.210      0.276 0.150   0.108
## 121       Source.Swab.Surface GENlog     32  0.026      0.159 0.078   0.131
## 122            SampleStage.N1 GENlog      2  0.000      0.020 0.008   0.115
## 123            SampleStage.N2 GENlog    137 -0.135     -0.196 0.104   0.061
## 124            SampleStage.F1 GENlog     40  0.010      0.051 0.034   0.062
## 125            SampleStage.F2 GENlog    158 -0.172     -0.216 0.107   0.070
## 126                Pig.Mixing TETlog     61 -0.089     -0.289 0.248   0.379
## 127                   Rodents TETlog     28 -0.049     -0.347 0.198   0.423
## 128                 Ruminants TETlog    183  0.610      0.660 0.468   0.452
## 129                   Insects TETlog    140 -0.396     -0.560 0.327   0.244
## 130 Disinfectant.QuatAmmonium TETlog    127  0.435      0.678 0.568   0.581
## 131              PressureWash TETlog    198 -1.097     -1.097 0.428   0.428
## 132        Obs.Entry.Measures TETlog    198 -0.979     -0.979 0.438   0.438
## 133                Obs.Shower TETlog    183 -0.499     -0.540 0.223   0.178
## 134              Obs.GI.signs TETlog    134 -0.307     -0.453 0.321   0.293
## 135            Obs.Resp.signs TETlog     77  0.202      0.520 0.344   0.372
## 136              Carbadox.dum TETlog      7  0.016      0.441 0.097   0.300
## 137              Tiamulin.dum TETlog     30  0.034      0.227 0.236   0.578
## 138            Lincomycin.dum TETlog    136  1.174      1.709 1.000   0.733
## 139                   OTC.dum TETlog      8  0.021      0.509 0.106   0.181
## 140             Roxarsone.dum TETlog     50 -0.057     -0.226 0.305   0.579
## 141         Virginiamycin.dum TETlog     71 -0.316     -0.880 0.538   0.558
## 142                 Pen.G.dum TETlog     24 -0.145     -1.193 0.471   0.769
## 143          Enrofloxacin.dum TETlog    172 -0.959     -1.104 0.632   0.547
## 144             Ceftiofur.dum TETlog     80  0.129      0.320 0.273   0.352
## 145           Site.Flow.Batch TETlog     18  0.013      0.143 0.176   0.581
## 146           Barn.Flow.Batch TETlog     43 -0.091     -0.419 0.200   0.217
## 147           Room.Flow.Batch TETlog     88 -0.127     -0.286 0.417   0.590
## 148              LabConf.Resp TETlog     32  0.043      0.268 0.140   0.251
## 149               Source.Feed TETlog     94  0.109      0.229 0.536   0.761
## 150              Source.Water TETlog    110  0.235      0.422 0.268   0.223
## 151             Source.Lagoon TETlog     98  0.073      0.148 0.277   0.381
## 152          Source.Fecal.Sow TETlog    103  0.304      0.584 0.366   0.306
## 153       Source.Swab.Surface TETlog     74  0.118      0.316 0.255   0.334
## 154            SampleStage.N1 TETlog      7  0.002      0.050 0.047   0.265
## 155            SampleStage.N2 TETlog     66  0.200      0.601 0.295   0.141
## 156            SampleStage.F1 TETlog    153  0.423      0.547 0.426   0.408
## 157            SampleStage.F2 TETlog     16  0.028      0.350 0.319   1.102
## 158                Pig.Mixing FFNlog     38  0.012      0.064 0.062   0.130
## 159                   Rodents FFNlog     88 -0.110     -0.248 0.147   0.120
## 160                 Ruminants FFNlog     60 -0.069     -0.227 0.126   0.127
## 161                   Insects FFNlog     50 -0.043     -0.168 0.090   0.106
## 162 Disinfectant.QuatAmmonium FFNlog     21  0.007      0.064 0.100   0.307
## 163              PressureWash FFNlog      3  0.002      0.142 0.033   0.277
## 164        Obs.Entry.Measures FFNlog     11 -0.006     -0.101 0.032   0.101
## 165                Obs.Shower FFNlog     15 -0.003     -0.043 0.049   0.177
## 166              Obs.GI.signs FFNlog     22 -0.002     -0.014 0.046   0.140
## 167            Obs.Resp.signs FFNlog     96 -0.149     -0.308 0.191   0.163
## 168              Carbadox.dum FFNlog      3  0.002      0.104 0.016   0.095
## 169              Tiamulin.dum FFNlog      9 -0.002     -0.047 0.036   0.171
## 170                   CTC.dum FFNlog    108 -0.081     -0.148 0.100   0.091
## 171            Lincomycin.dum FFNlog     65 -0.076     -0.230 0.175   0.242
## 172                   OTC.dum FFNlog     10  0.001      0.027 0.030   0.136
## 173             Roxarsone.dum FFNlog     23 -0.009     -0.081 0.059   0.160
## 174         Virginiamycin.dum FFNlog     41 -0.021     -0.103 0.081   0.153
## 175                 Pen.G.dum FFNlog     18  0.037      0.407 0.121   0.104
## 176          Enrofloxacin.dum FFNlog     47  0.068      0.286 0.143   0.152
## 177             Ceftiofur.dum FFNlog    111  0.126      0.225 0.141   0.116
## 178           Site.Flow.Batch FFNlog     12  0.009      0.140 0.072   0.268
## 179           Barn.Flow.Batch FFNlog     97  0.006      0.013 0.095   0.135
## 180           Room.Flow.Batch FFNlog     36  0.023      0.128 0.083   0.158
## 181              LabConf.Resp FFNlog     29  0.000     -0.003 0.047   0.124
## 182               Source.Feed FFNlog     65  0.095      0.290 0.179   0.202
## 183              Source.Water FFNlog    152  0.176      0.230 0.124   0.087
## 184             Source.Lagoon FFNlog     64  0.049      0.152 0.090   0.098
## 185          Source.Fecal.Sow FFNlog     26  0.019      0.148 0.075   0.155
## 186       Source.Swab.Surface FFNlog    142  0.176      0.245 0.149   0.118
## 187            SampleStage.N1 FFNlog     20  0.002      0.015 0.045   0.144
## 188            SampleStage.N2 FFNlog     32  0.010      0.059 0.058   0.135
## 189            SampleStage.F1 FFNlog     10 -0.009     -0.175 0.058   0.200
## 190            SampleStage.F2 FFNlog    157 -0.181     -0.228 0.135   0.110
## 191                Pig.Mixing NALlog     29 -0.027     -0.188 0.094   0.175
## 192                   Rodents NALlog     46  0.018      0.076 0.164   0.336
## 193                 Ruminants NALlog     96 -0.148     -0.305 0.269   0.318
## 194                   Insects NALlog     86  0.114      0.263 0.161   0.144
## 195 Disinfectant.QuatAmmonium NALlog     17  0.008      0.088 0.076   0.254
## 196              PressureWash NALlog    114  0.089      0.155 0.116   0.115
## 197        Obs.Entry.Measures NALlog    198  0.990      0.990 0.180   0.180
## 198                Obs.Shower NALlog      6  0.002      0.078 0.028   0.153
## 199              Obs.GI.signs NALlog    172 -0.193     -0.223 0.264   0.272
## 200            Obs.Resp.signs NALlog     54  0.026      0.097 0.151   0.279
## 201              Carbadox.dum NALlog      2  0.003      0.262 0.032   0.247
## 202              Tiamulin.dum NALlog     64  0.046      0.142 0.129   0.196
## 203                   CTC.dum NALlog      6  0.000      0.005 0.048   0.302
## 204            Lincomycin.dum NALlog    183  0.569      0.615 0.397   0.376
## 205                   OTC.dum NALlog     52  0.074      0.283 0.141   0.129
## 206             Roxarsone.dum NALlog      2 -0.003     -0.265 0.028   0.126
## 207         Virginiamycin.dum NALlog      7 -0.008     -0.222 0.080   0.396
## 208                 Pen.G.dum NALlog    198 -1.134     -1.134 0.243   0.243
## 209             Ceftiofur.dum NALlog    198 -0.790     -0.790 0.238   0.238
## 210           Site.Flow.Batch NALlog    198 -1.039     -1.039 0.223   0.223
## 211           Barn.Flow.Batch NALlog     93  0.211      0.449 0.260   0.193
## 212           Room.Flow.Batch NALlog      5  0.012      0.467 0.075   0.102
## 213              LabConf.Resp NALlog    198  1.057      1.057 0.141   0.141
## 214               Source.Feed NALlog    112 -0.142     -0.251 0.158   0.129
## 215              Source.Water NALlog    142 -0.184     -0.256 0.150   0.114
## 216             Source.Lagoon NALlog    139 -0.171     -0.243 0.152   0.124
## 217          Source.Fecal.Sow NALlog     14  0.004      0.053 0.051   0.192
## 218       Source.Swab.Surface NALlog     33 -0.027     -0.163 0.083   0.139
## 219            SampleStage.N1 NALlog     96  0.107      0.220 0.142   0.129
## 220            SampleStage.N2 NALlog      7 -0.001     -0.024 0.036   0.207
## 221            SampleStage.F1 NALlog     14 -0.012     -0.177 0.066   0.186
## 222            SampleStage.F2 NALlog     16 -0.003     -0.034 0.073   0.263
## 223                Pig.Mixing TELlog     98  0.082      0.166 0.131   0.143
## 224                   Rodents TELlog    151  0.344      0.451 0.276   0.226
## 225                 Ruminants TELlog    183  0.437      0.473 0.298   0.281
## 226                   Insects TELlog    115 -0.096     -0.165 0.153   0.170
## 227 Disinfectant.QuatAmmonium TELlog     38 -0.008     -0.042 0.114   0.260
## 228              PressureWash TELlog     66 -0.047     -0.140 0.141   0.216
## 229        Obs.Entry.Measures TELlog    101 -0.050     -0.097 0.096   0.116
## 230                Obs.Shower TELlog    169 -0.298     -0.349 0.203   0.174
## 231              Obs.GI.signs TELlog     71 -0.050     -0.138 0.110   0.147
## 232            Obs.Resp.signs TELlog    168  0.457      0.538 0.394   0.373
## 233              Carbadox.dum TELlog      4 -0.006     -0.319 0.049   0.152
## 234              Tiamulin.dum TELlog    191 -0.411     -0.426 0.244   0.235
## 235                   CTC.dum TELlog     15 -0.019     -0.251 0.081   0.171
## 236            Lincomycin.dum TELlog     55  0.004      0.013 0.157   0.300
## 237                   OTC.dum TELlog     51 -0.075     -0.290 0.164   0.207
## 238             Roxarsone.dum TELlog     62  0.081      0.259 0.150   0.160
## 239         Virginiamycin.dum TELlog     32  0.010      0.061 0.111   0.274
## 240                 Pen.G.dum TELlog      4 -0.003     -0.173 0.056   0.410
## 241          Enrofloxacin.dum TELlog     31 -0.021     -0.135 0.069   0.126
## 242             Ceftiofur.dum TELlog    129  0.199      0.306 0.190   0.151
## 243           Site.Flow.Batch TELlog     17  0.000     -0.004 0.054   0.190
## 244           Barn.Flow.Batch TELlog      8  0.004      0.091 0.020   0.046
## 245           Room.Flow.Batch TELlog     61 -0.054     -0.176 0.101   0.109
## 246              LabConf.Resp TELlog    146 -0.129     -0.175 0.185   0.196
## 247               Source.Feed TELlog     54  0.015      0.057 0.168   0.321
## 248              Source.Water TELlog     12  0.009      0.143 0.064   0.230
## 249             Source.Lagoon TELlog     61 -0.054     -0.174 0.106   0.124
## 250          Source.Fecal.Sow TELlog     77 -0.071     -0.183 0.133   0.159
## 251       Source.Swab.Surface TELlog     46  0.073      0.314 0.165   0.204
## 252            SampleStage.N1 TELlog     35  0.003      0.017 0.071   0.171
## 253            SampleStage.N2 TELlog     99 -0.115     -0.229 0.147   0.129
## 254            SampleStage.F1 TELlog     40  0.019      0.093 0.062   0.110
## 255            SampleStage.F2 TELlog     13  0.007      0.114 0.044   0.139
## 256                Pig.Mixing CLIlog     83  0.039      0.093 0.108   0.151
## 257                   Rodents CLIlog     54  0.022      0.079 0.139   0.259
## 258                 Ruminants CLIlog    104  0.194      0.369 0.271   0.274
## 259                   Insects CLIlog     82  0.054      0.129 0.131   0.179
## 260 Disinfectant.QuatAmmonium CLIlog     68  0.057      0.166 0.157   0.232
## 261              PressureWash CLIlog     83 -0.082     -0.196 0.171   0.219
## 262        Obs.Entry.Measures CLIlog     87 -0.021     -0.048 0.104   0.154
## 263                Obs.Shower CLIlog     97 -0.094     -0.192 0.150   0.165
## 264              Obs.GI.signs CLIlog     17  0.013      0.157 0.075   0.214
## 265            Obs.Resp.signs CLIlog    196  1.017      1.027 0.407   0.395
## 266              Tiamulin.dum CLIlog    192 -0.477     -0.492 0.252   0.241
## 267                   CTC.dum CLIlog     22 -0.038     -0.339 0.128   0.217
## 268            Lincomycin.dum CLIlog     51 -0.085     -0.332 0.182   0.217
## 269                   OTC.dum CLIlog     20 -0.033     -0.324 0.123   0.240
## 270             Roxarsone.dum CLIlog    137  0.295      0.426 0.288   0.253
## 271         Virginiamycin.dum CLIlog     45  0.006      0.027 0.149   0.315
## 272                 Pen.G.dum CLIlog     11  0.005      0.091 0.036   0.131
## 273          Enrofloxacin.dum CLIlog     11  0.000      0.004 0.044   0.195
## 274             Ceftiofur.dum CLIlog     26  0.011      0.085 0.086   0.227
## 275           Site.Flow.Batch CLIlog     44  0.027      0.122 0.113   0.215
## 276           Barn.Flow.Batch CLIlog     22  0.005      0.049 0.039   0.109
## 277           Room.Flow.Batch CLIlog     27 -0.010     -0.073 0.061   0.153
## 278              LabConf.Resp CLIlog    174 -0.239     -0.272 0.244   0.243
## 279               Source.Feed CLIlog    110 -0.185     -0.332 0.320   0.368
## 280              Source.Water CLIlog     95 -0.090     -0.188 0.207   0.267
## 281             Source.Lagoon CLIlog    194 -0.394     -0.402 0.172   0.164
## 282          Source.Fecal.Sow CLIlog    127 -0.120     -0.186 0.202   0.227
## 283       Source.Swab.Surface CLIlog     56  0.061      0.214 0.219   0.372
## 284            SampleStage.N1 CLIlog     46 -0.001     -0.006 0.101   0.212
## 285            SampleStage.N2 CLIlog     62 -0.057     -0.181 0.131   0.180
## 286            SampleStage.F1 CLIlog     28  0.013      0.095 0.073   0.174
## 287            SampleStage.F2 CLIlog     22  0.011      0.099 0.065   0.173
##       B_50 CI_lower CI_upper n_na
## 1    0.672    0.000    1.681    0
## 2    0.000   -1.289    1.593    0
## 3    2.413    0.000    4.669    0
## 4   -0.667   -1.733    0.000    0
## 5    0.000   -0.085    1.969    0
## 6    0.000   -1.526    0.000    0
## 7    0.000   -0.882    0.000    0
## 8   -0.768   -2.169    0.000    0
## 9    0.000   -1.035    0.000    0
## 10   2.861    0.627    5.209    0
## 11  -1.240   -2.316    0.000    0
## 12   0.000   -0.330    1.533    0
## 13   0.000   -0.458    1.250    0
## 14   1.842    0.000    3.749    0
## 15   0.000   -0.055    0.000    0
## 16   0.000    0.000    0.051    0
## 17   0.000    0.000    0.000    0
## 18   0.000   -0.661    0.840    0
## 19   0.000   -0.160    0.447    0
## 20   0.546    0.000    1.411    0
## 21   0.000   -1.282    0.003    0
## 22  -1.504   -2.561    0.000    0
## 23  -1.083   -3.118    0.485    0
## 24  -0.930   -2.088    0.000    0
## 25  -1.448   -2.400   -0.479    0
## 26  -1.003   -2.102    0.000    0
## 27  -0.951   -2.037    0.000    0
## 28   0.000    0.000    1.344    0
## 29   0.000   -0.943    0.369    0
## 30   0.000    0.000    0.854    0
## 31   0.000    0.000    0.153    0
## 32  -0.142   -0.930    0.000    0
## 33   0.000   -0.333    1.094    0
## 34  -0.673   -1.831    0.000    0
## 35   0.818    0.000    1.606    0
## 36   0.000    0.000    0.000    0
## 37   0.000   -0.447    0.000    0
## 38   0.000   -0.172    0.494    0
## 39   2.068    1.527    2.741    0
## 40   0.000    0.000    0.000    0
## 41  -0.577   -1.278    0.000    0
## 42   0.000    0.000    0.570    0
## 43   0.000    0.000    0.216    0
## 44   0.237   -0.005    1.023    0
## 45   0.000    0.000    0.833    0
## 46   1.053    0.000    2.691    0
## 47   0.000   -0.619    0.307    0
## 48   0.000    0.000    1.026    0
## 49  -2.369   -3.217   -1.659    0
## 50  -1.896   -2.518   -1.261    0
## 51  -1.947   -2.551   -1.091    0
## 52   0.000    0.000    0.967    0
## 53   0.000   -1.022    0.000    0
## 54   2.206    1.770    2.777    0
## 55  -0.127   -0.676    0.000    0
## 56   0.000   -0.653    0.000    0
## 57   0.000   -0.648    0.000    0
## 58   0.000   -0.271    0.003    0
## 59   0.000   -0.373    0.501    0
## 60   0.000    0.000    0.644    0
## 61   0.000    0.000    0.240    0
## 62   0.000   -0.324    0.360    0
## 63   0.000    0.000    0.957    0
## 64   0.275    0.000    1.009    0
## 65   0.000   -0.659    0.838    0
## 66   1.512    0.000    2.928    0
## 67  -0.175   -0.806    0.070    0
## 68   0.000    0.000    1.247    0
## 69   0.000   -1.217    0.000    0
## 70   0.000   -0.598    0.000    0
## 71  -0.607   -1.421    0.000    0
## 72   0.000   -0.629    0.000    0
## 73   1.753    0.000    3.390    0
## 74  -0.802   -1.464    0.000    0
## 75   0.000   -0.117    0.924    0
## 76   0.000   -0.505    0.220    0
## 77   1.237    0.000    2.419    0
## 78   0.000   -0.108    0.000    0
## 79   0.000    0.000    0.000    0
## 80   0.000   -0.475    0.000    0
## 81   0.000    0.000    0.597    0
## 82   0.000    0.000    0.183    0
## 83   0.000    0.000    0.729    0
## 84   0.000   -0.532    0.118    0
## 85  -0.628   -1.408    0.000    0
## 86  -0.530   -1.706    0.136    0
## 87  -0.779   -1.284    0.000    0
## 88  -0.825   -1.340   -0.081    0
## 89  -0.526   -1.414    0.000    0
## 90  -0.595   -1.201    0.000    0
## 91   0.000    0.000    0.614    0
## 92   0.000   -0.410    0.000    0
## 93   0.000    0.000    0.447    0
## 94   0.000   -0.023    0.081    0
## 95   0.000   -0.214    0.000    0
## 96  -0.220   -0.472    0.000    0
## 97   0.000    0.000    0.305    0
## 98   0.000   -0.211    0.000    0
## 99   0.000   -0.403    0.000    0
## 100  0.000    0.000    0.009    0
## 101  0.000    0.000    0.121    0
## 102  0.000   -0.179    0.000    0
## 103  0.000    0.000    0.000    0
## 104  0.000   -0.072    0.112    0
## 105  0.000    0.000    0.000    0
## 106  0.000   -0.076    0.000    0
## 107 -0.086   -0.280    0.000    0
## 108  0.000   -0.425    0.000    0
## 109 -0.072   -0.374    0.000    0
## 110  0.000   -0.187    0.000    0
## 111  0.000   -0.163    0.000    0
## 112  0.000   -0.189    0.000    0
## 113  0.000   -0.208    0.000    0
## 114  0.000   -0.308    0.000    0
## 115  0.000    0.000    0.357    0
## 116  0.000   -0.184    0.000    0
## 117  0.000   -0.432    0.000    0
## 118  0.000    0.000    0.205    0
## 119  0.000    0.000    0.253    0
## 120  0.228    0.000    0.509    0
## 121  0.000    0.000    0.250    0
## 122  0.000    0.000    0.000    0
## 123 -0.164   -0.314    0.000    0
## 124  0.000   -0.031    0.118    0
## 125 -0.189   -0.357    0.000    0
## 126  0.000   -0.715    0.359    0
## 127  0.000   -0.604    0.000    0
## 128  0.625   -0.256    1.370    0
## 129 -0.399   -0.966    0.000    0
## 130  0.296   -0.244    1.672    0
## 131 -1.021   -2.179   -0.424    0
## 132 -0.993   -1.692   -0.116    0
## 133 -0.519   -0.920    0.000    0
## 134 -0.311   -1.043    0.045    0
## 135  0.000   -0.082    0.982    0
## 136  0.000    0.000    0.202    0
## 137  0.000   -0.295    0.795    0
## 138  1.246    0.000    3.253    0
## 139  0.000    0.000    0.402    0
## 140  0.000   -0.814    0.392    0
## 141  0.000   -1.554    0.000    0
## 142  0.000   -1.785    0.000    0
## 143 -0.960   -2.130    0.000    0
## 144  0.000   -0.255    0.767    0
## 145  0.000   -0.181    0.302    0
## 146  0.000   -0.712    0.000    0
## 147  0.000   -1.309    0.434    0
## 148  0.000    0.000    0.552    0
## 149  0.000   -1.053    1.158    0
## 150  0.181    0.000    0.855    0
## 151  0.000   -0.472    0.616    0
## 152  0.189    0.000    1.099    0
## 153  0.000   -0.287    0.740    0
## 154  0.000    0.000    0.009    0
## 155  0.000    0.000    0.805    0
## 156  0.432    0.000    0.901    0
## 157  0.000   -0.288    0.430    0
## 158  0.000   -0.128    0.175    0
## 159  0.000   -0.455    0.000    0
## 160  0.000   -0.362    0.000    0
## 161  0.000   -0.269    0.000    0
## 162  0.000   -0.144    0.266    0
## 163  0.000    0.000    0.000    0
## 164  0.000   -0.106    0.000    0
## 165  0.000   -0.127    0.000    0
## 166  0.000   -0.134    0.132    0
## 167  0.000   -0.584    0.000    0
## 168  0.000    0.000    0.000    0
## 169  0.000   -0.046    0.000    0
## 170 -0.042   -0.299    0.000    0
## 171  0.000   -0.560    0.146    0
## 172  0.000    0.000    0.052    0
## 173  0.000   -0.204    0.032    0
## 174  0.000   -0.224    0.112    0
## 175  0.000    0.000    0.474    0
## 176  0.000    0.000    0.498    0
## 177  0.115    0.000    0.435    0
## 178  0.000    0.000    0.111    0
## 179  0.000   -0.189    0.248    0
## 180  0.000    0.000    0.235    0
## 181  0.000   -0.126    0.134    0
## 182  0.000   -0.176    0.513    0
## 183  0.194    0.000    0.395    0
## 184  0.000   -0.008    0.258    0
## 185  0.000    0.000    0.208    0
## 186  0.180    0.000    0.464    0
## 187  0.000   -0.082    0.110    0
## 188  0.000   -0.089    0.167    0
## 189  0.000   -0.147    0.000    0
## 190 -0.176   -0.417    0.000    0
## 191  0.000   -0.361    0.000    0
## 192  0.000   -0.261    0.567    0
## 193  0.000   -0.812    0.296    0
## 194  0.000    0.000    0.482    0
## 195  0.000   -0.100    0.243    0
## 196  0.046    0.000    0.323    0
## 197  1.014    0.576    1.329    0
## 198  0.000    0.000    0.000    0
## 199 -0.249   -0.636    0.308    0
## 200  0.000   -0.309    0.423    0
## 201  0.000    0.000    0.000    0
## 202  0.000   -0.041    0.302    0
## 203  0.000    0.000    0.000    0
## 204  0.542    0.000    1.386    0
## 205  0.000    0.000    0.431    0
## 206  0.000    0.000    0.000    0
## 207  0.000   -0.021    0.000    0
## 208 -1.179   -1.540   -0.608    0
## 209 -0.787   -1.255   -0.371    0
## 210 -1.044   -1.417   -0.614    0
## 211  0.000    0.000    0.757    0
## 212  0.000    0.000    0.027    0
## 213  1.066    0.780    1.314    0
## 214 -0.145   -0.444    0.000    0
## 215 -0.181   -0.463    0.000    0
## 216 -0.164   -0.494    0.000    0
## 217  0.000   -0.063    0.137    0
## 218  0.000   -0.267    0.000    0
## 219  0.000    0.000    0.448    0
## 220  0.000    0.000    0.003    0
## 221  0.000   -0.210    0.000    0
## 222  0.000   -0.140    0.119    0
## 223  0.000   -0.089    0.424    0
## 224  0.348    0.000    0.879    0
## 225  0.395    0.000    1.107    0
## 226 -0.044   -0.377    0.208    0
## 227  0.000   -0.336    0.228    0
## 228  0.000   -0.411    0.227    0
## 229  0.000   -0.277    0.127    0
## 230 -0.305   -0.717    0.000    0
## 231  0.000   -0.294    0.135    0
## 232  0.400   -0.018    1.377    0
## 233  0.000    0.000    0.000    0
## 234 -0.428   -0.823    0.000    0
## 235  0.000   -0.281    0.000    0
## 236  0.000   -0.369    0.319    0
## 237  0.000   -0.537    0.000    0
## 238  0.000    0.000    0.463    0
## 239  0.000   -0.260    0.303    0
## 240  0.000    0.000    0.000    0
## 241  0.000   -0.250    0.000    0
## 242  0.194    0.000    0.595    0
## 243  0.000   -0.109    0.102    0
## 244  0.000    0.000    0.060    0
## 245  0.000   -0.338    0.000    0
## 246 -0.093   -0.558    0.196    0
## 247  0.000   -0.296    0.514    0
## 248  0.000    0.000    0.266    0
## 249  0.000   -0.333    0.000    0
## 250  0.000   -0.445    0.008    0
## 251  0.000    0.000    0.509    0
## 252  0.000   -0.232    0.199    0
## 253  0.000   -0.456    0.000    0
## 254  0.000   -0.005    0.178    0
## 255  0.000    0.000    0.167    0
## 256  0.000   -0.180    0.270    0
## 257  0.000   -0.255    0.386    0
## 258  0.069    0.000    0.955    0
## 259  0.000   -0.177    0.381    0
## 260  0.000   -0.216    0.480    0
## 261  0.000   -0.544    0.179    0
## 262  0.000   -0.260    0.195    0
## 263  0.000   -0.457    0.028    0
## 264  0.000   -0.042    0.312    0
## 265  1.052    0.274    1.743    0
## 266 -0.499   -0.877    0.005    0
## 267  0.000   -0.569    0.000    0
## 268  0.000   -0.544    0.000    0
## 269  0.000   -0.366    0.000    0
## 270  0.248    0.000    0.823    0
## 271  0.000   -0.332    0.410    0
## 272  0.000    0.000    0.089    0
## 273  0.000   -0.099    0.000    0
## 274  0.000   -0.150    0.231    0
## 275  0.000   -0.155    0.349    0
## 276  0.000   -0.003    0.138    0
## 277  0.000   -0.155    0.051    0
## 278 -0.245   -0.670    0.289    0
## 279  0.000   -0.827    0.466    0
## 280  0.000   -0.491    0.490    0
## 281 -0.385   -0.683   -0.077    0
## 282 -0.078   -0.569    0.198    0
## 283  0.000   -0.245    0.643    0
## 284  0.000   -0.248    0.221    0
## 285  0.000   -0.456    0.029    0
## 286  0.000   -0.040    0.179    0
## 287  0.000    0.000    0.203    0
## 
## 
## OMEGA EDGES:
##        Y1     Y2 n_non0 Omega_bar SE_Omega Omega_bar_non0 SE_Omega_non0
## 1  AZIlog CIPlog    175    -0.021    0.016         -0.023         0.016
## 2  AZIlog ERYlog    198     0.841    0.025          0.841         0.025
## 3  AZIlog GENlog    188    -0.056    0.031         -0.059         0.028
## 4  AZIlog TETlog     44    -0.004    0.013         -0.017         0.022
## 5  AZIlog FFNlog    198    -0.240    0.027         -0.240         0.027
## 6  AZIlog NALlog    163    -0.005    0.016         -0.007         0.017
## 7  AZIlog TELlog    198    -0.164    0.065         -0.164         0.065
## 8  AZIlog CLIlog    198     0.101    0.061          0.101         0.061
## 9  CIPlog ERYlog     12     0.001    0.003          0.012         0.007
## 10 CIPlog GENlog     70     0.004    0.014          0.013         0.021
## 11 CIPlog TETlog    197     0.055    0.041          0.056         0.041
## 12 CIPlog FFNlog    185     0.073    0.051          0.078         0.049
## 13 CIPlog NALlog    198     0.908    0.010          0.908         0.010
## 14 CIPlog TELlog    198     0.055    0.029          0.055         0.029
## 15 CIPlog CLIlog     52    -0.005    0.011         -0.020         0.013
## 16 ERYlog GENlog      5    -0.001    0.006         -0.031         0.027
## 17 ERYlog TETlog    105     0.017    0.023          0.031         0.023
## 18 ERYlog FFNlog      3     0.000    0.005         -0.032         0.036
## 19 ERYlog NALlog      8    -0.001    0.003         -0.013         0.009
## 20 ERYlog TELlog    198     0.450    0.039          0.450         0.039
## 21 ERYlog CLIlog    198     0.186    0.077          0.186         0.077
## 22 GENlog TETlog    195     0.132    0.054          0.134         0.052
## 23 GENlog FFNlog    198     0.278    0.050          0.278         0.050
## 24 GENlog NALlog     96     0.015    0.022          0.031         0.022
## 25 GENlog TELlog    180     0.058    0.046          0.064         0.044
## 26 GENlog CLIlog    154     0.075    0.062          0.096         0.054
## 27 TETlog FFNlog    198     0.340    0.044          0.340         0.044
## 28 TETlog NALlog     89    -0.024    0.039         -0.054         0.042
## 29 TETlog TELlog    198     0.246    0.042          0.246         0.042
## 30 TETlog CLIlog    198    -0.201    0.047         -0.201         0.047
## 31 FFNlog NALlog    150    -0.060    0.053         -0.080         0.046
## 32 FFNlog TELlog    198     0.380    0.038          0.380         0.038
## 33 FFNlog CLIlog      6     0.001    0.007          0.031         0.030
## 34 NALlog TELlog     59    -0.014    0.028         -0.048         0.031
## 35 NALlog CLIlog    123    -0.016    0.017         -0.026         0.015
## 36 TELlog CLIlog    198     0.341    0.044          0.341         0.044
##    Omega_50 CI_lower CI_upper n_na
## 1    -0.018   -0.060    0.000    0
## 2     0.840    0.796    0.884    0
## 3    -0.058   -0.113    0.000    0
## 4     0.000   -0.039    0.000    0
## 5    -0.239   -0.297   -0.193    0
## 6    -0.005   -0.031    0.029    0
## 7    -0.159   -0.282   -0.060    0
## 8     0.103   -0.001    0.211    0
## 9     0.000    0.000    0.010    0
## 10    0.000   -0.016    0.043    0
## 11    0.045    0.002    0.160    0
## 12    0.066    0.000    0.173    0
## 13    0.908    0.892    0.926    0
## 14    0.049    0.013    0.130    0
## 15    0.000   -0.037    0.000    0
## 16    0.000    0.000    0.000    0
## 17    0.003    0.000    0.079    0
## 18    0.000    0.000    0.000    0
## 19    0.000   -0.008    0.000    0
## 20    0.447    0.385    0.521    0
## 21    0.191    0.056    0.305    0
## 22    0.134    0.013    0.224    0
## 23    0.280    0.177    0.368    0
## 24    0.000    0.000    0.065    0
## 25    0.056   -0.022    0.147    0
## 26    0.076    0.000    0.187    0
## 27    0.339    0.256    0.427    0
## 28    0.000   -0.139    0.000    0
## 29    0.247    0.173    0.326    0
## 30   -0.198   -0.287   -0.104    0
## 31   -0.050   -0.159    0.000    0
## 32    0.383    0.304    0.448    0
## 33    0.000    0.000    0.001    0
## 34    0.000   -0.091    0.000    0
## 35   -0.011   -0.052    0.000    0
## 36    0.339    0.267    0.428    0
```

#### Genetic

```
X <- conv.gen_cent

model.cg = l1ML_Main(
  Y = Y, 
  X = X, 
  initializer = 'Lasso', 
  screening = T,
  ss = T,
  alpha = 0.05,
  nboot = 20
)
```

```
## Step 0: screening is on, proceed with debiased Lasso in conjunction with BH correction, the cut-off for p-values is set at 0.05 .
## Step 1: Penalized LS initialization with Lasso .
## Step 2: Alternate update.
```

```
## Warning in l1ML_Main(Y = Y, X = X, initializer = "Lasso", screening = T, : l1ML_Main(): alternate update is broken manually at iteration =50
```

```
## Step 3: Refitting B Matrix.
## Step 4: Stability selection with a total number of 20 bootstrapped samples ...25%...50%...75%...Done!
## Step 5: Refitting with weighted glasso ...Done.
## Returns: B.est, Theta.est, BICvalue.
```

```
model.cg_formatted <- format.chain.graph(model.cg, x.names = colnames(X), y.names = colnames(Y))
print(display.chain.graph(model.cg_formatted))
```

```
## 
## Lambda: 
## Edges: 58 (59%)
## 
## Beta Matrix:
##                 AZIlog CIPlog ERYlog GENlog TETlog FFNlog NALlog TELlog CLIlog
## X23S_A2075G     7.539 -0.176  5.011 -0.315  0.000 -0.652  0.000  1.835  1.995
## X50S_L22_A103V  0.000  0.304  0.000  0.000  0.769  0.000  0.154  0.367  0.000
## aadE_Cc         0.000  0.000  0.000  0.000  0.000  0.000  0.068  0.000  0.000
## acr3            0.000  0.367  0.000  0.150  0.884 -0.300 -0.107  0.000  0.000
## aph.3pr_IIIa    0.000  0.000  0.000 -0.294  1.029 -0.355  0.176  0.133  0.000
## blaOXA_193      0.000  0.000 -0.106  0.000 -0.698  0.000  0.000 -0.160  0.000
## blaOXA_489      2.013 -0.484  1.021 -0.304 -0.909  0.000 -0.163  0.000  0.585
## blaOXA_578      2.381 -0.419  1.494 -0.309 -0.691  0.000 -0.227  0.000  0.446
## gyrA_T86I      -1.448  6.277 -1.008 -0.129 -0.353 -0.169  3.656 -0.611 -0.714
## rpsL_K43R      -1.761  0.000 -0.843  0.000  1.653 -0.141 -0.134  0.000  0.000
## tet.O           1.517 -0.346  0.930  0.000  0.000 -0.237  0.000  0.000  0.524
## 
## 
## Rho: 
## Edges: 27 (75%)
## 
## Omega Matrix: 
##        AZIlog CIPlog ERYlog GENlog TETlog FFNlog NALlog TELlog CLIlog
## AZIlog  0.000 -0.062  0.826 -0.060 -0.042 -0.229  0.017 -0.094  0.094
## CIPlog -0.063  0.000  0.000  0.034  0.116  0.212  0.622  0.135  0.000
## ERYlog  0.826  0.000  0.000  0.000  0.097  0.000  0.000  0.402  0.220
## GENlog -0.060  0.034  0.000  0.000  0.118  0.193  0.140  0.070  0.107
## TETlog -0.042  0.116  0.097  0.118  0.000  0.297  0.000  0.135 -0.223
## FFNlog -0.229  0.212  0.000  0.193  0.297  0.000  0.000  0.388  0.000
## NALlog  0.017  0.622  0.000  0.140  0.000  0.000  0.000 -0.056  0.000
## TELlog -0.094  0.135  0.402  0.070  0.135  0.388 -0.056  0.000  0.308
## CLIlog  0.094  0.000  0.220  0.107 -0.223  0.000  0.000  0.308  0.000
## 
## 
## NULL
```

```
R2.tab <- ChainGraph_R2_v4(
  X, 
  Y, 
  Beta = model.cg_formatted$B.est, 
  Omega = model.cg_formatted$Omega.est
)

print(R2.tab)
```

```
##          Y
## AZIlog AZI
## CIPlog CIP
## ERYlog ERY
## GENlog GEN
## TETlog TET
## FFNlog FFN
## NALlog NAL
## TELlog TEL
## CLIlog CLI
##                                                                                               X_set
## AZIlog                             X23S_A2075G, blaOXA_489, blaOXA_578, gyrA_T86I, rpsL_K43R, tet.O
## CIPlog                  X23S_A2075G, X50S_L22_A103V, acr3, blaOXA_489, blaOXA_578, gyrA_T86I, tet.O
## ERYlog                 X23S_A2075G, blaOXA_193, blaOXA_489, blaOXA_578, gyrA_T86I, rpsL_K43R, tet.O
## GENlog                           X23S_A2075G, acr3, aph.3pr_IIIa, blaOXA_489, blaOXA_578, gyrA_T86I
## TETlog X50S_L22_A103V, acr3, aph.3pr_IIIa, blaOXA_193, blaOXA_489, blaOXA_578, gyrA_T86I, rpsL_K43R
## FFNlog                                 X23S_A2075G, acr3, aph.3pr_IIIa, gyrA_T86I, rpsL_K43R, tet.O
## NALlog    X50S_L22_A103V, aadE_Cc, acr3, aph.3pr_IIIa, blaOXA_489, blaOXA_578, gyrA_T86I, rpsL_K43R
## TELlog                             X23S_A2075G, X50S_L22_A103V, aph.3pr_IIIa, blaOXA_193, gyrA_T86I
## CLIlog                                        X23S_A2075G, blaOXA_489, blaOXA_578, gyrA_T86I, tet.O
##              R2_X                                  Y_set   R2_e_Yj     R2_Yj
## AZIlog 0.28769123 CIP, ERY, GEN, TET, FFN, NAL, TEL, CLI 0.9525752 0.6785277
## CIPlog 0.79747206           AZI, GEN, TET, FFN, NAL, TEL 0.7032468 0.1424271
## ERYlog 0.28131628                     AZI, TET, TEL, CLI 0.9657611 0.6940767
## GENlog 0.06377537      AZI, CIP, TET, FFN, NAL, TEL, CLI 0.4221920 0.3952665
## TETlog 0.08348312      AZI, CIP, ERY, GEN, FFN, TEL, CLI 0.5056028 0.4633935
## FFNlog 0.02788250                AZI, CIP, GEN, TET, TEL 0.6877530 0.6685767
## NALlog 0.76591116                     AZI, CIP, GEN, TEL 0.5757966 0.1347875
## TELlog 0.15931978 AZI, CIP, ERY, GEN, TET, FFN, NAL, CLI 0.8903062 0.7484628
## CLIlog 0.20230708                AZI, ERY, GEN, TET, TEL 0.8530922 0.6805056
##         R2_total
## AZIlog 0.9662189
## CIPlog 0.9398992
## ERYlog 0.9753930
## GENlog 0.4590419
## TETlog 0.5468766
## FFNlog 0.6964592
## NALlog 0.9006987
## TELlog 0.9077826
## CLIlog 0.8828127
```

##### Bootstrap

```
#cl <- parallel::makeCluster(n_cl)
#doParallel::registerDoParallel(cl)

model.cg_bs <- cg.bootstrap(
  Y = Y, 
  X = X, 
  b = B,
  n_b = n_b_conv,
  lambda = model.cg$lambda, 
  rho = model.cg$rho,
  initializer = 'Lasso', 
  screening = T,
  alpha = 0.05,
  nboot = 20,
  cluster = cl,
  seed.val = seeds[2]
)
```

```
## Starting parallel bootstrap at 2025-01-10 21:28:04.187375
```

```
## Ending bootstrap at 2025-01-10 21:39:05.781656
```

```
#stopCluster(cl)

summary.cg_bootstrap_results(model.cg_bs)
```

```
## SAMPLE SIZES
## Original:  683 
## Subsample size:  342 
## 
## BOOTSTRAPS
## Total fit subsamples:  200 
## Subsamples for summary stats: 200 
## Subsamples that returned NAs: 0 
## 
## PENALTIES
## Lambda:  0.05925224 
## Rho:  0.05671878 
## 
## CI alpha: 95 %
## 
## BETA EDGES:
##                 X      Y n_non0  B_bar B_bar_non0  SE_B SE_non0   B_50 CI_lower
## 1     X23S_A2075G AZIlog    200  7.109      7.109 0.600   0.600  7.162    5.940
## 2  X50S_L22_A103V AZIlog      7  0.032      0.925 0.176   0.265  0.000    0.000
## 3         aadE_Cc AZIlog      2  0.008      0.843 0.094   0.598  0.000    0.000
## 4            acr3 AZIlog     83 -0.558     -1.345 0.791   0.670  0.000   -2.153
## 5    aph.3pr_IIIa AZIlog     41 -0.186     -0.905 0.469   0.652  0.000   -1.418
## 6      blaOXA_193 AZIlog     82 -0.624     -1.523 0.796   0.416  0.000   -2.153
## 7      blaOXA_489 AZIlog    151  1.599      2.117 1.158   0.821  1.754    0.000
## 8      blaOXA_578 AZIlog    154  2.035      2.643 1.326   0.818  2.235    0.000
## 9       gyrA_T86I AZIlog    196 -1.506     -1.537 0.357   0.288 -1.526   -2.068
## 10      rpsL_K43R AZIlog    133 -1.124     -1.690 0.966   0.665 -1.192   -2.771
## 11          tet.O AZIlog    156  1.156      1.482 0.743   0.473  1.293    0.000
## 12    X23S_A2075G CIPlog    132 -0.220     -0.333 0.242   0.226 -0.151   -0.764
## 13 X50S_L22_A103V CIPlog    175  0.321      0.366 0.198   0.167  0.325    0.000
## 14        aadE_Cc CIPlog     15  0.005      0.070 0.027   0.072  0.000    0.000
## 15           acr3 CIPlog    199  0.437      0.439 0.119   0.115  0.433    0.196
## 16   aph.3pr_IIIa CIPlog    140  0.185      0.264 0.160   0.125  0.179    0.000
## 17     blaOXA_193 CIPlog      3  0.000      0.030 0.027   0.271  0.000    0.000
## 18     blaOXA_489 CIPlog    197 -0.463     -0.470 0.157   0.147 -0.480   -0.729
## 19     blaOXA_578 CIPlog    198 -0.476     -0.481 0.213   0.209 -0.452   -0.885
## 20      gyrA_T86I CIPlog    200  6.286      6.286 0.177   0.177  6.299    5.946
## 21      rpsL_K43R CIPlog     71  0.220      0.619 0.348   0.305  0.000    0.000
## 22          tet.O CIPlog    153 -0.291     -0.380 0.211   0.156 -0.311   -0.651
## 23    X23S_A2075G ERYlog    200  4.663      4.663 0.514   0.514  4.665    3.699
## 24 X50S_L22_A103V ERYlog     32  0.040      0.247 0.152   0.308  0.000    0.000
## 25        aadE_Cc ERYlog     18  0.030      0.333 0.114   0.210  0.000    0.000
## 26           acr3 ERYlog     38 -0.082     -0.432 0.287   0.537  0.000   -0.869
## 27   aph.3pr_IIIa ERYlog     46  0.105      0.458 0.233   0.273  0.000    0.000
## 28     blaOXA_193 ERYlog     92 -0.414     -0.901 0.557   0.486  0.000   -1.517
## 29     blaOXA_489 ERYlog    144  0.858      1.191 0.748   0.616  0.891   -0.093
## 30     blaOXA_578 ERYlog    170  1.393      1.639 0.919   0.768  1.525    0.000
## 31      gyrA_T86I ERYlog    199 -1.016     -1.021 0.266   0.256 -1.034   -1.450
## 32      rpsL_K43R ERYlog     78 -0.341     -0.874 0.524   0.488  0.000   -1.544
## 33          tet.O ERYlog    164  0.723      0.882 0.482   0.378  0.823    0.000
## 34    X23S_A2075G GENlog    138 -0.217     -0.314 0.170   0.104 -0.260   -0.515
## 35 X50S_L22_A103V GENlog     56 -0.070     -0.250 0.121   0.086  0.000   -0.370
## 36        aadE_Cc GENlog     15 -0.016     -0.218 0.061   0.075  0.000   -0.248
## 37           acr3 GENlog    198  0.245      0.247 0.076   0.072  0.242    0.121
## 38   aph.3pr_IIIa GENlog    120 -0.182     -0.303 0.172   0.112 -0.187   -0.501
## 39     blaOXA_193 GENlog     78  0.105      0.269 0.139   0.073  0.000    0.000
## 40     blaOXA_489 GENlog    175 -0.218     -0.250 0.134   0.113 -0.211   -0.486
## 41     blaOXA_578 GENlog    136 -0.185     -0.272 0.161   0.120 -0.185   -0.486
## 42      gyrA_T86I GENlog    150 -0.099     -0.131 0.082   0.068 -0.098   -0.253
## 43      rpsL_K43R GENlog     71 -0.120     -0.339 0.189   0.164  0.000   -0.574
## 44          tet.O GENlog     54  0.018      0.067 0.103   0.192  0.000   -0.203
## 45    X23S_A2075G TETlog     30  0.150      1.003 0.370   0.236  0.000    0.000
## 46 X50S_L22_A103V TETlog    189  0.698      0.738 0.329   0.290  0.699    0.000
## 47        aadE_Cc TETlog     87  0.275      0.631 0.332   0.167  0.000    0.000
## 48           acr3 TETlog    200  1.017      1.017 0.159   0.159  1.030    0.688
## 49   aph.3pr_IIIa TETlog    196  1.056      1.078 0.340   0.307  1.079    0.470
## 50     blaOXA_193 TETlog     99 -0.353     -0.712 0.375   0.163  0.000   -0.979
## 51     blaOXA_489 TETlog    178 -0.771     -0.866 0.488   0.430 -0.771   -1.697
## 52     blaOXA_578 TETlog    107 -0.401     -0.749 0.424   0.272 -0.337   -1.197
## 53      gyrA_T86I TETlog    121 -0.285     -0.471 0.262   0.159 -0.318   -0.799
## 54      rpsL_K43R TETlog    194  1.615      1.665 0.646   0.588  1.660    0.000
## 55          tet.O TETlog     90  0.156      0.348 0.582   0.831  0.000   -0.980
## 56    X23S_A2075G FFNlog    198 -0.630     -0.636 0.150   0.137 -0.634   -0.875
## 57 X50S_L22_A103V FFNlog     36 -0.046     -0.256 0.107   0.097  0.000   -0.364
## 58        aadE_Cc FFNlog     53  0.020      0.075 0.066   0.112  0.000   -0.064
## 59           acr3 FFNlog     88 -0.103     -0.233 0.132   0.096  0.000   -0.402
## 60   aph.3pr_IIIa FFNlog     79 -0.079     -0.199 0.156   0.195  0.000   -0.510
## 61     blaOXA_193 FFNlog      5 -0.001     -0.025 0.014   0.093  0.000    0.000
## 62     blaOXA_489 FFNlog     75  0.021      0.055 0.136   0.219  0.000   -0.208
## 63     blaOXA_578 FFNlog     55 -0.020     -0.074 0.129   0.240  0.000   -0.361
## 64      gyrA_T86I FFNlog    162 -0.146     -0.180 0.112   0.097 -0.139   -0.353
## 65      rpsL_K43R FFNlog     49 -0.060     -0.245 0.154   0.227  0.000   -0.495
## 66          tet.O FFNlog    146 -0.153     -0.210 0.159   0.150 -0.152   -0.473
## 67    X23S_A2075G NALlog     33 -0.020     -0.121 0.068   0.127  0.000   -0.239
## 68 X50S_L22_A103V NALlog    134  0.136      0.203 0.124   0.096  0.133    0.000
## 69        aadE_Cc NALlog     97  0.048      0.100 0.058   0.041  0.000    0.000
## 70           acr3 NALlog     18 -0.007     -0.073 0.046   0.141  0.000   -0.148
## 71   aph.3pr_IIIa NALlog    176  0.258      0.293 0.155   0.131  0.259    0.000
## 72     blaOXA_193 NALlog     15  0.008      0.107 0.035   0.078  0.000    0.000
## 73     blaOXA_489 NALlog    140 -0.124     -0.177 0.105   0.080 -0.130   -0.321
## 74     blaOXA_578 NALlog    164 -0.209     -0.255 0.140   0.111 -0.217   -0.468
## 75      gyrA_T86I NALlog    200  3.674      3.674 0.096   0.096  3.677    3.468
## 76      rpsL_K43R NALlog     93 -0.066     -0.141 0.136   0.172  0.000   -0.419
## 77          tet.O NALlog     34 -0.018     -0.104 0.072   0.147  0.000   -0.204
## 78    X23S_A2075G TELlog    199  1.597      1.605 0.349   0.331  1.625    0.881
## 79 X50S_L22_A103V TELlog    144  0.178      0.247 0.205   0.203  0.180   -0.156
## 80        aadE_Cc TELlog     55  0.011      0.040 0.083   0.156  0.000   -0.132
## 81           acr3 TELlog     65  0.075      0.230 0.143   0.165  0.000    0.000
## 82   aph.3pr_IIIa TELlog    101  0.148      0.294 0.195   0.180  0.000   -0.002
## 83     blaOXA_193 TELlog    156 -0.251     -0.321 0.324   0.335 -0.114   -0.926
## 84     blaOXA_489 TELlog     90  0.109      0.243 0.360   0.507  0.000   -0.645
## 85     blaOXA_578 TELlog     87  0.351      0.807 0.469   0.371  0.000    0.000
## 86      gyrA_T86I TELlog    200 -0.585     -0.585 0.197   0.197 -0.603   -0.917
## 87      rpsL_K43R TELlog     35  0.058      0.329 0.156   0.225  0.000    0.000
## 88          tet.O TELlog     40  0.044      0.220 0.102   0.116  0.000    0.000
## 89    X23S_A2075G CLIlog    200  1.905      1.905 0.428   0.428  1.909    1.036
## 90 X50S_L22_A103V CLIlog     16 -0.003     -0.035 0.096   0.347  0.000   -0.189
## 91        aadE_Cc CLIlog      9 -0.012     -0.274 0.082   0.295  0.000   -0.202
## 92           acr3 CLIlog     31  0.043      0.278 0.142   0.257  0.000   -0.001
## 93   aph.3pr_IIIa CLIlog     44  0.086      0.392 0.189   0.206  0.000    0.000
## 94     blaOXA_193 CLIlog     46 -0.078     -0.341 0.250   0.431  0.000   -0.655
## 95     blaOXA_489 CLIlog    160  0.559      0.699 0.448   0.391  0.584   -0.049
## 96     blaOXA_578 CLIlog    167  0.593      0.711 0.540   0.515  0.612   -0.467
## 97      gyrA_T86I CLIlog    199 -0.694     -0.697 0.186   0.180 -0.709   -0.994
## 98      rpsL_K43R CLIlog     92  0.026      0.057 0.305   0.449  0.000   -0.664
## 99          tet.O CLIlog    176  0.398      0.452 0.282   0.256  0.454   -0.142
##    CI_upper n_na
## 1     8.188    0
## 2     0.652    0
## 3     0.000    0
## 4     0.000    0
## 5     0.123    0
## 6     0.000    0
## 7     3.788    0
## 8     4.006    0
## 9    -0.936    0
## 10    0.000    0
## 11    2.349    0
## 12    0.000    0
## 13    0.679    0
## 14    0.094    0
## 15    0.635    0
## 16    0.525    0
## 17    0.000    0
## 18   -0.126    0
## 19   -0.100    0
## 20    6.598    0
## 21    1.060    0
## 22    0.000    0
## 23    5.557    0
## 24    0.427    0
## 25    0.340    0
## 26    0.230    0
## 27    0.702    0
## 28    0.000    0
## 29    2.407    0
## 30    2.796    0
## 31   -0.566    0
## 32    0.087    0
## 33    1.540    0
## 34    0.000    0
## 35    0.000    0
## 36    0.000    0
## 37    0.394    0
## 38    0.000    0
## 39    0.367    0
## 40    0.000    0
## 41    0.000    0
## 42    0.000    0
## 43    0.000    0
## 44    0.263    0
## 45    1.156    0
## 46    1.275    0
## 47    0.885    0
## 48    1.330    0
## 49    1.641    0
## 50    0.000    0
## 51    0.000    0
## 52    0.000    0
## 53    0.000    0
## 54    2.763    0
## 55    1.530    0
## 56   -0.270    0
## 57    0.000    0
## 58    0.229    0
## 59    0.000    0
## 60    0.125    0
## 61    0.000    0
## 62    0.394    0
## 63    0.270    0
## 64    0.000    0
## 65    0.000    0
## 66    0.134    0
## 67    0.000    0
## 68    0.395    0
## 69    0.171    0
## 70    0.003    0
## 71    0.575    0
## 72    0.118    0
## 73    0.000    0
## 74    0.000    0
## 75    3.839    0
## 76    0.051    0
## 77    0.083    0
## 78    2.164    0
## 79    0.550    0
## 80    0.181    0
## 81    0.450    0
## 82    0.633    0
## 83    0.167    0
## 84    0.948    0
## 85    1.269    0
## 86   -0.249    0
## 87    0.490    0
## 88    0.367    0
## 89    2.720    0
## 90    0.167    0
## 91    0.000    0
## 92    0.517    0
## 93    0.630    0
## 94    0.587    0
## 95    1.508    0
## 96    1.494    0
## 97   -0.408    0
## 98    0.678    0
## 99    0.872    0
## 
## 
## OMEGA EDGES:
##        Y1     Y2 n_non0 Omega_bar SE_Omega Omega_bar_non0 SE_Omega_non0
## 1  AZIlog CIPlog    197    -0.050    0.018         -0.051         0.017
## 2  AZIlog ERYlog    200     0.823    0.030          0.823         0.030
## 3  AZIlog GENlog    115    -0.019    0.023         -0.034         0.021
## 4  AZIlog TETlog    120    -0.033    0.045         -0.055         0.047
## 5  AZIlog FFNlog    200    -0.239    0.030         -0.239         0.030
## 6  AZIlog NALlog     84    -0.004    0.009         -0.008         0.012
## 7  AZIlog TELlog    200    -0.101    0.061         -0.101         0.061
## 8  AZIlog CLIlog    200     0.111    0.059          0.111         0.059
## 9  CIPlog ERYlog      4     0.001    0.005          0.037         0.015
## 10 CIPlog GENlog    192     0.042    0.042          0.044         0.042
## 11 CIPlog TETlog    200     0.129    0.036          0.129         0.036
## 12 CIPlog FFNlog    200     0.229    0.046          0.229         0.046
## 13 CIPlog NALlog    200     0.617    0.059          0.617         0.059
## 14 CIPlog TELlog    200     0.110    0.030          0.110         0.030
## 15 CIPlog CLIlog     24    -0.003    0.010         -0.021         0.023
## 16 ERYlog GENlog      3     0.000    0.002         -0.001         0.022
## 17 ERYlog TETlog    187     0.075    0.052          0.080         0.050
## 18 ERYlog FFNlog      1     0.000    0.004         -0.052            NA
## 19 ERYlog NALlog      9     0.000    0.002         -0.003         0.008
## 20 ERYlog TELlog    200     0.410    0.038          0.410         0.038
## 21 ERYlog CLIlog    200     0.202    0.072          0.202         0.072
## 22 GENlog TETlog    194     0.106    0.051          0.109         0.048
## 23 GENlog FFNlog    200     0.204    0.047          0.204         0.047
## 24 GENlog NALlog    196     0.133    0.050          0.136         0.047
## 25 GENlog TELlog    198     0.078    0.045          0.079         0.044
## 26 GENlog CLIlog    101     0.035    0.047          0.069         0.045
## 27 TETlog FFNlog    200     0.294    0.044          0.294         0.044
## 28 TETlog NALlog    126    -0.023    0.034         -0.037         0.036
## 29 TETlog TELlog    200     0.137    0.049          0.137         0.049
## 30 TETlog CLIlog    200    -0.194    0.043         -0.194         0.043
## 31 FFNlog NALlog    126    -0.027    0.039         -0.043         0.041
## 32 FFNlog TELlog    200     0.379    0.044          0.379         0.044
## 33 FFNlog CLIlog     47     0.017    0.041          0.071         0.059
## 34 NALlog TELlog     60    -0.009    0.018         -0.029         0.023
## 35 NALlog CLIlog     29    -0.003    0.010         -0.024         0.014
## 36 TELlog CLIlog    200     0.313    0.050          0.313         0.050
##    Omega_50 CI_lower CI_upper n_na
## 1    -0.050   -0.085   -0.013    0
## 2     0.825    0.767    0.873    0
## 3    -0.009   -0.079    0.000    0
## 4    -0.011   -0.151    0.000    0
## 5    -0.239   -0.296   -0.181    0
## 6     0.000   -0.024    0.013    0
## 7    -0.094   -0.235    0.005    0
## 8     0.108    0.010    0.229    0
## 9     0.000    0.000    0.000    0
## 10    0.040   -0.037    0.120    0
## 11    0.129    0.065    0.204    0
## 12    0.229    0.143    0.315    0
## 13    0.622    0.483    0.710    0
## 14    0.109    0.053    0.169    0
## 15    0.000   -0.030    0.000    0
## 16    0.000    0.000    0.000    0
## 17    0.073    0.000    0.182    0
## 18    0.000    0.000    0.000    0
## 19    0.000   -0.002    0.000    0
## 20    0.407    0.348    0.489    0
## 21    0.214    0.066    0.312    0
## 22    0.100    0.000    0.199    0
## 23    0.204    0.112    0.285    0
## 24    0.136    0.021    0.222    0
## 25    0.079   -0.010    0.159    0
## 26    0.004    0.000    0.143    0
## 27    0.293    0.213    0.377    0
## 28   -0.009   -0.103    0.000    0
## 29    0.138    0.043    0.222    0
## 30   -0.190   -0.284   -0.110    0
## 31   -0.012   -0.123    0.000    0
## 32    0.381    0.266    0.452    0
## 33    0.000    0.000    0.146    0
## 34    0.000   -0.058    0.000    0
## 35    0.000   -0.034    0.000    0
## 36    0.315    0.214    0.402    0
```

#### Environmental + Genetic

```
X <- conv.env_gen_cent

model.cg = l1ML_Main(
  Y = Y, 
  X = X, 
  initializer = 'Lasso', 
  screening = T,
  ss = T,
  alpha = 0.05,
  nboot = 20
)
```

```
## Step 0: screening is on, proceed with debiased Lasso in conjunction with BH correction, the cut-off for p-values is set at 0.05 .
## Step 1: Penalized LS initialization with Lasso .
## Step 2: Alternate update.
```

```
## Warning in l1ML_Main(Y = Y, X = X, initializer = "Lasso", screening = T, : l1ML_Main(): alternate update is broken manually at iteration =50
```

```
## Step 3: Refitting B Matrix.
## Step 4: Stability selection with a total number of 20 bootstrapped samples ...25%...50%...75%...Done!
## Step 5: Refitting with weighted glasso ...Done.
## Returns: B.est, Theta.est, BICvalue.
```

```
model.cg_formatted <- format.chain.graph(model.cg, x.names = colnames(X), y.names = colnames(Y))
print(display.chain.graph(model.cg_formatted))
```

```
## 
## Lambda: 
## Edges: 124 (30%)
## 
## Beta Matrix:
##                            AZIlog CIPlog ERYlog GENlog TETlog FFNlog NALlog
## X23S_A2075G                6.147 -0.320  3.961 -0.285  0.000 -0.848  0.000
## X50S_L22_A103V             0.000  0.237  0.000 -0.238  0.708  0.000  0.000
## aadE_Cc                    0.000  0.000  0.266  0.000  0.000  0.000  0.043
## acr3                       0.000  0.382  0.000  0.056  0.042 -0.188  0.000
## aph.3pr_IIIa               0.000  0.129  0.000  0.000  0.228  0.000  0.255
## blaOXA_193                -1.388  0.000 -0.901  0.000 -0.509  0.000  0.000
## blaOXA_489                 1.579 -0.322  0.810 -0.226 -0.207  0.103  0.000
## blaOXA_578                 1.868 -0.278  1.139 -0.108 -0.515  0.000 -0.041
## gyrA_T86I                 -1.758  6.222 -1.159  0.000  0.000 -0.117  3.662
## rpsL_K43R                 -0.695  0.000  0.000 -0.405  0.000  0.000 -0.318
## tet.O                      1.118 -0.299  0.717  0.000  0.000 -0.156  0.000
## Pig.Mixing                 0.000 -0.344  0.000  0.000  0.000  0.000  0.000
## Enter.Hrs                  0.000  0.000  0.000  0.000  0.000  0.000  0.000
## Rodents                    0.000  0.000  0.000 -0.132  0.000  0.000  0.000
## Ruminants                  1.309  0.000  0.885  0.000  0.679  0.000  0.000
## Insects                   -0.111  0.000  0.000  0.000 -0.200  0.000 -0.067
## Visitors.Wk                0.000  0.000  0.000  0.000  0.000  0.000  0.000
## Disinfectant.QuatAmmonium  0.000  0.000  0.000  0.000  0.000  0.000  0.000
## PressureWash               0.000  0.000  0.000  0.000 -1.119  0.000  0.000
## Obs.Entry.Measures         0.000  0.000  0.000  0.000 -0.891  0.000  0.000
## Obs.Shower                 0.000  0.000  0.000  0.000 -0.542  0.000 -0.074
## Obs.GI.signs               0.000  0.000 -0.089  0.000 -0.206  0.000  0.000
## Obs.Resp.signs             2.097  0.000  1.308  0.000  0.000  0.000  0.000
## Carbadox.dum               0.000  0.000  0.000  0.000  0.000  0.000  0.000
## Tiamulin.dum               0.000  0.000  0.000  0.000  0.000  0.000  0.000
## CTC.dum                    0.000  0.000  0.000 -0.139  0.000 -0.095  0.000
## Lincomycin.dum             0.520  0.147  0.229 -0.295  1.553  0.000  0.000
## OTC.dum                    0.000  0.000  0.000  0.000  0.000  0.000  0.000
## Roxarsone.dum              0.832  0.000  0.790  0.000  0.000  0.000  0.000
## Virginiamycin.dum          0.000  0.000  0.000  0.000  0.000  0.000  0.000
## Pen.G.dum                  0.000 -0.242  0.000  0.000  0.000  0.000 -0.029
## Enrofloxacin.dum           0.000  0.000  0.000  0.000 -1.336  0.000  0.000
## Ceftiofur.dum              0.000  0.000  0.000  0.000  0.000  0.000  0.000
## Site.Flow.Batch           -0.929  0.000 -0.424  0.000  0.000  0.000 -0.045
## Barn.Flow.Batch            0.930 -0.225  0.510 -0.089  0.000  0.000  0.000
## Room.Flow.Batch            0.000  0.000  0.000  0.000  0.000  0.000  0.000
## LabConf.Resp              -0.589  0.182 -0.291  0.000  0.000  0.000  0.000
## Source.Feed               -0.635  0.000 -0.301  0.000  0.000  0.000  0.000
## Source.Water               0.000  0.000 -0.397  0.000  0.324  0.000  0.000
## Source.Lagoon             -1.047  0.000 -0.608  0.000  0.000  0.000  0.000
## Source.Fecal.Sow          -0.340  0.000 -0.145  0.111  0.205  0.000  0.000
## Source.Swab.Surface        0.000  0.119  0.000  0.000  0.000  0.079  0.000
## SampleStage.N1             0.000  0.000  0.000  0.000  0.000  0.000  0.000
## SampleStage.N2             0.000  0.000  0.000 -0.228  0.000  0.000  0.000
## SampleStage.F1             0.000  0.000  0.000  0.000  0.263  0.000  0.000
## SampleStage.F2             0.000  0.000  0.000 -0.121  0.000 -0.100  0.000
##                           TELlog CLIlog
## X23S_A2075G                1.467  1.711
## X50S_L22_A103V             0.260  0.000
## aadE_Cc                    0.060  0.000
## acr3                       0.071  0.000
## aph.3pr_IIIa               0.256  0.180
## blaOXA_193                -0.742 -0.431
## blaOXA_489                 0.000  0.582
## blaOXA_578                 0.000  0.343
## gyrA_T86I                 -0.539 -0.634
## rpsL_K43R                  0.000  0.000
## tet.O                      0.000  0.532
## Pig.Mixing                 0.000  0.000
## Enter.Hrs                  0.000  0.000
## Rodents                    0.000  0.000
## Ruminants                  0.347  0.085
## Insects                    0.000  0.153
## Visitors.Wk                0.000  0.000
## Disinfectant.QuatAmmonium  0.000  0.000
## PressureWash               0.000  0.000
## Obs.Entry.Measures         0.000  0.000
## Obs.Shower                 0.000  0.000
## Obs.GI.signs               0.000  0.000
## Obs.Resp.signs             0.409  0.647
## Carbadox.dum               0.000  0.000
## Tiamulin.dum               0.000 -0.010
## CTC.dum                    0.000  0.000
## Lincomycin.dum             0.000  0.000
## OTC.dum                    0.000  0.000
## Roxarsone.dum              0.000  0.000
## Virginiamycin.dum          0.000  0.000
## Pen.G.dum                  0.000  0.000
## Enrofloxacin.dum           0.000  0.000
## Ceftiofur.dum              0.000  0.000
## Site.Flow.Batch            0.000  0.000
## Barn.Flow.Batch            0.000  0.000
## Room.Flow.Batch            0.000  0.000
## LabConf.Resp              -0.118 -0.107
## Source.Feed                0.000  0.000
## Source.Water               0.000  0.000
## Source.Lagoon              0.000 -0.338
## Source.Fecal.Sow           0.000  0.000
## Source.Swab.Surface        0.230  0.289
## SampleStage.N1             0.000  0.000
## SampleStage.N2            -0.121  0.000
## SampleStage.F1             0.000  0.000
## SampleStage.F2             0.000  0.000
## 
## 
## Rho: 
## Edges: 27 (75%)
## 
## Omega Matrix: 
##        AZIlog CIPlog ERYlog GENlog TETlog FFNlog NALlog TELlog CLIlog
## AZIlog  0.000 -0.043  0.829 -0.040  0.000 -0.204 -0.012 -0.157  0.108
## CIPlog -0.043  0.000  0.000  0.051  0.111  0.217  0.623  0.108  0.000
## ERYlog  0.829  0.000  0.000  0.000  0.013  0.000  0.000  0.445  0.205
## GENlog -0.040  0.051  0.000  0.000  0.187  0.142  0.133  0.072  0.073
## TETlog  0.000  0.111  0.013  0.187  0.000  0.346 -0.066  0.199 -0.197
## FFNlog -0.204  0.217  0.000  0.142  0.346  0.000  0.000  0.337  0.000
## NALlog -0.012  0.623  0.000  0.133 -0.066  0.000  0.000 -0.005  0.000
## TELlog -0.158  0.108  0.444  0.072  0.199  0.337 -0.005  0.000  0.321
## CLIlog  0.108  0.000  0.205  0.073 -0.197  0.000  0.000  0.321  0.000
## 
## 
## NULL
```

```
R2.tab <- ChainGraph_R2_v4(
  X, 
  Y, 
  Beta = model.cg_formatted$B.est, 
  Omega = model.cg_formatted$Omega.est
)

print(R2.tab)
```

```
##          Y
## AZIlog AZI
## CIPlog CIP
## ERYlog ERY
## GENlog GEN
## TETlog TET
## FFNlog FFN
## NALlog NAL
## TELlog TEL
## CLIlog CLI
##                                                                                                                                                                                                                                                                 X_set
## AZIlog                  X23S_A2075G, blaOXA_193, blaOXA_489, blaOXA_578, gyrA_T86I, rpsL_K43R, tet.O, Ruminants, Insects, Obs.Resp.signs, Lincomycin.dum, Roxarsone.dum, Site.Flow.Batch, Barn.Flow.Batch, LabConf.Resp, Source.Feed, Source.Lagoon, Source.Fecal.Sow
## CIPlog                                                                           X23S_A2075G, X50S_L22_A103V, acr3, aph.3pr_IIIa, blaOXA_489, blaOXA_578, gyrA_T86I, tet.O, Pig.Mixing, Lincomycin.dum, Pen.G.dum, Barn.Flow.Batch, LabConf.Resp, Source.Swab.Surface
## ERYlog X23S_A2075G, aadE_Cc, blaOXA_193, blaOXA_489, blaOXA_578, gyrA_T86I, tet.O, Ruminants, Obs.GI.signs, Obs.Resp.signs, Lincomycin.dum, Roxarsone.dum, Site.Flow.Batch, Barn.Flow.Batch, LabConf.Resp, Source.Feed, Source.Water, Source.Lagoon, Source.Fecal.Sow
## GENlog                                                                                      X23S_A2075G, X50S_L22_A103V, acr3, blaOXA_489, blaOXA_578, rpsL_K43R, Rodents, CTC.dum, Lincomycin.dum, Barn.Flow.Batch, Source.Fecal.Sow, SampleStage.N2, SampleStage.F2
## TETlog                       X50S_L22_A103V, acr3, aph.3pr_IIIa, blaOXA_193, blaOXA_489, blaOXA_578, Ruminants, Insects, PressureWash, Obs.Entry.Measures, Obs.Shower, Obs.GI.signs, Lincomycin.dum, Enrofloxacin.dum, Source.Water, Source.Fecal.Sow, SampleStage.F1
## FFNlog                                                                                                                                                                  X23S_A2075G, acr3, blaOXA_489, gyrA_T86I, tet.O, CTC.dum, Source.Swab.Surface, SampleStage.F2
## NALlog                                                                                                                                                       aadE_Cc, aph.3pr_IIIa, blaOXA_578, gyrA_T86I, rpsL_K43R, Insects, Obs.Shower, Pen.G.dum, Site.Flow.Batch
## TELlog                                                                                                  X23S_A2075G, X50S_L22_A103V, aadE_Cc, acr3, aph.3pr_IIIa, blaOXA_193, gyrA_T86I, Ruminants, Obs.Resp.signs, LabConf.Resp, Source.Swab.Surface, SampleStage.N2
## CLIlog                                                                            X23S_A2075G, aph.3pr_IIIa, blaOXA_193, blaOXA_489, blaOXA_578, gyrA_T86I, tet.O, Ruminants, Insects, Obs.Resp.signs, Tiamulin.dum, LabConf.Resp, Source.Lagoon, Source.Swab.Surface
##              R2_X                                  Y_set   R2_e_Yj     R2_Yj
## AZIlog 0.36041222      CIP, ERY, GEN, FFN, NAL, TEL, CLI 0.9493615 0.6072000
## CIPlog 0.80314137           AZI, GEN, TET, FFN, NAL, TEL 0.7035107 0.1384922
## ERYlog 0.34828108                     AZI, TET, TEL, CLI 0.9651370 0.6289980
## GENlog 0.10324125      AZI, CIP, TET, FFN, NAL, TEL, CLI 0.4384191 0.3931562
## TETlog 0.19043431      CIP, ERY, GEN, FFN, NAL, TEL, CLI 0.5627187 0.4555577
## FFNlog 0.05220664                AZI, CIP, GEN, TET, TEL 0.6860163 0.6502017
## NALlog 0.76508045                AZI, CIP, GEN, TET, TEL 0.5762018 0.1353611
## TELlog 0.21133282 AZI, CIP, ERY, GEN, TET, FFN, NAL, CLI 0.8919716 0.7034687
## CLIlog 0.24027263                AZI, ERY, GEN, TET, TEL 0.8542199 0.6489743
##         R2_total
## AZIlog 0.9676122
## CIPlog 0.9416335
## ERYlog 0.9772791
## GENlog 0.4963974
## TETlog 0.6459920
## FFNlog 0.7024083
## NALlog 0.9004415
## TELlog 0.9148015
## CLIlog 0.8892469
```

##### Bootstrap

```
#cl <- parallel::makeCluster(n_cl)
#doParallel::registerDoParallel(cl)

model.cg_bs <- cg.bootstrap(
  Y = Y, 
  X = X, 
  b = B,
  n_b = n_b_conv,
  lambda = model.cg$lambda, 
  rho = model.cg$rho,
  initializer = 'Lasso', 
  screening = T,
  alpha = 0.05,
  nboot = 20,
  cluster = cl,
  seed.val = seeds[3]
)
```

```
## Starting parallel bootstrap at 2025-01-10 21:39:24.793776
```

```
## Ending bootstrap at 2025-01-10 21:51:19.813908
```

```
#stopCluster(cl)

summary.cg_bootstrap_results(model.cg_bs)
```

```
## Warning in summary.cg_bootstrap_results(model.cg_bs): Target number of
## subsamples (b = 200) exceeds available valid subsamples (b = 199).
```

```
## Warning in summary.cg_bootstrap_results(model.cg_bs): Some edges had no
## instances of non-zero values in the included sub-samples.
```

```
## SAMPLE SIZES
## Original:  683 
## Subsample size:  342 
## 
## BOOTSTRAPS
## Total fit subsamples:  200 
## Subsamples for summary stats: 199 
## Subsamples that returned NAs: 1 
## 
## PENALTIES
## Lambda:  0.07487072 
## Rho:  0.05671878 
## 
## CI alpha: 95 %
## 
## BETA EDGES:
##                             X      Y n_non0  B_bar B_bar_non0  SE_B SE_non0
## 1                 X23S_A2075G AZIlog    199  5.920      5.920 0.721   0.721
## 2              X50S_L22_A103V AZIlog     34  0.128      0.748 0.309   0.308
## 3                     aadE_Cc AZIlog     25  0.103      0.821 0.295   0.320
## 4                        acr3 AZIlog     81 -0.307     -0.755 0.683   0.902
## 5                aph.3pr_IIIa AZIlog     45 -0.076     -0.336 0.384   0.759
## 6                  blaOXA_193 AZIlog    174 -1.132     -1.294 0.562   0.388
## 7                  blaOXA_489 AZIlog    174  1.483      1.696 0.852   0.684
## 8                  blaOXA_578 AZIlog    184  1.911      2.067 0.935   0.789
## 9                   gyrA_T86I AZIlog    199 -1.680     -1.680 0.404   0.404
## 10                  rpsL_K43R AZIlog     94 -0.425     -0.899 0.679   0.741
## 11                      tet.O AZIlog    183  1.043      1.134 0.534   0.454
## 12                 Pig.Mixing AZIlog     69  0.180      0.520 0.322   0.351
## 13                    Rodents AZIlog     59 -0.164     -0.553 0.524   0.847
## 14                  Ruminants AZIlog    188  1.544      1.635 0.842   0.777
## 15                    Insects AZIlog    104 -0.267     -0.511 0.372   0.374
## 16  Disinfectant.QuatAmmonium AZIlog     65 -0.005     -0.016 0.461   0.811
## 17               PressureWash AZIlog      2  0.000      0.014 0.048   0.681
## 18         Obs.Entry.Measures AZIlog     14  0.052      0.746 0.219   0.413
## 19                 Obs.Shower AZIlog     54 -0.149     -0.549 0.290   0.301
## 20               Obs.GI.signs AZIlog     52 -0.141     -0.539 0.270   0.255
## 21             Obs.Resp.signs AZIlog    192  2.116      2.193 0.926   0.848
## 22               Carbadox.dum AZIlog      1  0.000     -0.086 0.006   0.000
## 23               Tiamulin.dum AZIlog     63 -0.208     -0.657 0.398   0.454
## 24                    CTC.dum AZIlog      6  0.013      0.439 0.100   0.411
## 25             Lincomycin.dum AZIlog     87  0.317      0.726 0.585   0.699
## 26                    OTC.dum AZIlog     37  0.056      0.304 0.227   0.453
## 27              Roxarsone.dum AZIlog    121  0.651      1.071 0.652   0.499
## 28          Virginiamycin.dum AZIlog      2 -0.009     -0.863 0.095   0.573
## 29                  Pen.G.dum AZIlog      5  0.022      0.867 0.150   0.444
## 30           Enrofloxacin.dum AZIlog     50  0.172      0.685 0.340   0.331
## 31              Ceftiofur.dum AZIlog     39 -0.068     -0.347 0.343   0.717
## 32            Site.Flow.Batch AZIlog    105 -0.552     -1.046 0.628   0.478
## 33            Barn.Flow.Batch AZIlog    179  0.823      0.915 0.432   0.351
## 34            Room.Flow.Batch AZIlog      4 -0.004     -0.194 0.074   0.557
## 35               LabConf.Resp AZIlog    161 -0.719     -0.889 0.493   0.385
## 36                Source.Feed AZIlog    133 -0.591     -0.884 0.886   0.957
## 37               Source.Water AZIlog     58 -0.210     -0.721 0.444   0.555
## 38              Source.Lagoon AZIlog    183 -1.003     -1.090 0.507   0.428
## 39           Source.Fecal.Sow AZIlog    128 -0.501     -0.779 0.499   0.413
## 40        Source.Swab.Surface AZIlog     48 -0.087     -0.361 0.365   0.678
## 41             SampleStage.N1 AZIlog     59  0.246      0.828 0.415   0.313
## 42             SampleStage.N2 AZIlog     74 -0.078     -0.209 0.250   0.376
## 43             SampleStage.F1 AZIlog     42  0.057      0.272 0.173   0.290
## 44             SampleStage.F2 AZIlog     19 -0.021     -0.220 0.197   0.619
## 45                X23S_A2075G CIPlog    175 -0.193     -0.220 0.150   0.141
## 46             X50S_L22_A103V CIPlog    166  0.242      0.290 0.155   0.122
## 47                    aadE_Cc CIPlog     16  0.005      0.062 0.025   0.068
## 48                       acr3 CIPlog    195  0.403      0.412 0.148   0.137
## 49               aph.3pr_IIIa CIPlog    116  0.136      0.234 0.142   0.108
## 50                 blaOXA_193 CIPlog     11  0.012      0.214 0.052   0.082
## 51                 blaOXA_489 CIPlog    191 -0.365     -0.380 0.146   0.127
## 52                 blaOXA_578 CIPlog    192 -0.283     -0.293 0.142   0.134
## 53                  gyrA_T86I CIPlog    199  6.207      6.207 0.183   0.183
## 54                  rpsL_K43R CIPlog     12  0.022      0.360 0.148   0.512
## 55                      tet.O CIPlog    137 -0.240     -0.348 0.196   0.134
## 56                 Pig.Mixing CIPlog    165 -0.284     -0.342 0.180   0.139
## 57                    Rodents CIPlog     89 -0.078     -0.173 0.101   0.079
## 58                  Ruminants CIPlog     80  0.068      0.169 0.119   0.135
## 59                    Insects CIPlog      5  0.004      0.146 0.031   0.150
## 60  Disinfectant.QuatAmmonium CIPlog     28  0.003      0.019 0.084   0.227
## 61               PressureWash CIPlog      2 -0.002     -0.152 0.015   0.013
## 62         Obs.Entry.Measures CIPlog     52 -0.047     -0.179 0.115   0.163
## 63                 Obs.Shower CIPlog      9 -0.005     -0.104 0.026   0.070
## 64               Obs.GI.signs CIPlog     15 -0.002     -0.021 0.028   0.104
## 65             Obs.Resp.signs CIPlog     62 -0.049     -0.159 0.105   0.135
## 66               Carbadox.dum CIPlog      7  0.002      0.068 0.022   0.102
## 67                    CTC.dum CIPlog      1  0.001      0.195 0.014   0.000
## 68             Lincomycin.dum CIPlog    128  0.232      0.361 0.251   0.226
## 69                    OTC.dum CIPlog     34  0.021      0.120 0.056   0.081
## 70              Roxarsone.dum CIPlog     11 -0.011     -0.202 0.048   0.060
## 71          Virginiamycin.dum CIPlog     27 -0.015     -0.113 0.056   0.110
## 72                  Pen.G.dum CIPlog    119 -0.161     -0.270 0.164   0.125
## 73           Enrofloxacin.dum CIPlog     35 -0.020     -0.114 0.057   0.087
## 74              Ceftiofur.dum CIPlog     64 -0.108     -0.337 0.178   0.146
## 75            Site.Flow.Batch CIPlog      4  0.004      0.189 0.037   0.211
## 76            Barn.Flow.Batch CIPlog    133 -0.174     -0.260 0.149   0.104
## 77            Room.Flow.Batch CIPlog      3 -0.004     -0.240 0.037   0.220
## 78               LabConf.Resp CIPlog    134  0.154      0.229 0.125   0.078
## 79                Source.Feed CIPlog     37  0.002      0.010 0.152   0.355
## 80               Source.Water CIPlog      9  0.003      0.066 0.033   0.149
## 81              Source.Lagoon CIPlog     61  0.069      0.226 0.118   0.100
## 82           Source.Fecal.Sow CIPlog     61  0.062      0.201 0.101   0.073
## 83        Source.Swab.Surface CIPlog    110  0.138      0.250 0.146   0.104
## 84             SampleStage.N1 CIPlog     36 -0.005     -0.028 0.080   0.189
## 85             SampleStage.N2 CIPlog     85  0.066      0.154 0.089   0.069
## 86             SampleStage.F1 CIPlog     28  0.021      0.148 0.065   0.109
## 87             SampleStage.F2 CIPlog     56 -0.026     -0.092 0.061   0.085
## 88                X23S_A2075G ERYlog    199  3.862      3.862 0.527   0.527
## 89             X50S_L22_A103V ERYlog     66  0.140      0.423 0.258   0.284
## 90                    aadE_Cc ERYlog     89  0.175      0.392 0.266   0.271
## 91                       acr3 ERYlog     70 -0.073     -0.207 0.309   0.496
## 92               aph.3pr_IIIa ERYlog     91  0.123      0.269 0.261   0.332
## 93                 blaOXA_193 ERYlog    166 -0.770     -0.923 0.440   0.301
## 94                 blaOXA_489 ERYlog    154  0.800      1.034 0.573   0.426
## 95                 blaOXA_578 ERYlog    181  1.254      1.379 0.653   0.544
## 96                  gyrA_T86I ERYlog    199 -1.062     -1.062 0.271   0.271
## 97                  rpsL_K43R ERYlog     70 -0.036     -0.102 0.319   0.534
## 98                      tet.O ERYlog    179  0.651      0.724 0.344   0.280
## 99                 Pig.Mixing ERYlog     51  0.076      0.296 0.164   0.201
## 100                   Rodents ERYlog     51 -0.129     -0.504 0.310   0.434
## 101                 Ruminants ERYlog    192  1.065      1.104 0.540   0.509
## 102                   Insects ERYlog     84 -0.101     -0.239 0.190   0.230
## 103 Disinfectant.QuatAmmonium ERYlog     66 -0.014     -0.043 0.245   0.426
## 104              PressureWash ERYlog     10 -0.013     -0.254 0.071   0.209
## 105        Obs.Entry.Measures ERYlog     10  0.018      0.352 0.095   0.263
## 106                Obs.Shower ERYlog     79 -0.155     -0.390 0.239   0.228
## 107              Obs.GI.signs ERYlog     82 -0.120     -0.290 0.187   0.188
## 108            Obs.Resp.signs ERYlog    187  1.229      1.307 0.649   0.587
## 109              Tiamulin.dum ERYlog    117 -0.156     -0.265 0.237   0.259
## 110                   CTC.dum ERYlog      3  0.003      0.218 0.050   0.423
## 111            Lincomycin.dum ERYlog    101  0.302      0.594 0.399   0.373
## 112                   OTC.dum ERYlog     55 -0.046     -0.168 0.109   0.150
## 113             Roxarsone.dum ERYlog    150  0.579      0.768 0.447   0.346
## 114         Virginiamycin.dum ERYlog      8 -0.005     -0.113 0.054   0.264
## 115                 Pen.G.dum ERYlog      1  0.003      0.558 0.040   0.000
## 116          Enrofloxacin.dum ERYlog     10 -0.002     -0.030 0.073   0.339
## 117             Ceftiofur.dum ERYlog     37  0.018      0.098 0.177   0.406
## 118           Site.Flow.Batch ERYlog     60 -0.148     -0.491 0.274   0.284
## 119           Barn.Flow.Batch ERYlog    146  0.386      0.526 0.283   0.188
## 120           Room.Flow.Batch ERYlog      2 -0.001     -0.073 0.008   0.042
## 121              LabConf.Resp ERYlog    138 -0.330     -0.475 0.285   0.218
## 122               Source.Feed ERYlog    119 -0.263     -0.440 0.445   0.503
## 123              Source.Water ERYlog    110 -0.337     -0.609 0.343   0.214
## 124             Source.Lagoon ERYlog    182 -0.571     -0.624 0.291   0.243
## 125          Source.Fecal.Sow ERYlog    119 -0.229     -0.383 0.263   0.238
## 126       Source.Swab.Surface ERYlog     40 -0.065     -0.323 0.216   0.388
## 127            SampleStage.N1 ERYlog     36  0.068      0.377 0.172   0.221
## 128            SampleStage.N2 ERYlog     30 -0.029     -0.193 0.101   0.193
## 129            SampleStage.F1 ERYlog     19  0.013      0.140 0.059   0.138
## 130            SampleStage.F2 ERYlog     22  0.000      0.002 0.095   0.292
## 131               X23S_A2075G GENlog    162 -0.219     -0.269 0.151   0.120
## 132            X50S_L22_A103V GENlog     78 -0.123     -0.313 0.162   0.085
## 133                   aadE_Cc GENlog      9 -0.004     -0.095 0.021   0.030
## 134                      acr3 GENlog    161  0.157      0.194 0.108   0.085
## 135              aph.3pr_IIIa GENlog     71 -0.108     -0.302 0.159   0.111
## 136                blaOXA_193 GENlog     41  0.042      0.204 0.087   0.059
## 137                blaOXA_489 GENlog    172 -0.242     -0.280 0.134   0.100
## 138                blaOXA_578 GENlog    145 -0.166     -0.227 0.141   0.116
## 139                 gyrA_T86I GENlog     96 -0.049     -0.102 0.064   0.057
## 140                 rpsL_K43R GENlog    114 -0.184     -0.321 0.203   0.168
## 141                     tet.O GENlog     36  0.015      0.085 0.086   0.189
## 142                Pig.Mixing GENlog     47 -0.031     -0.133 0.063   0.056
## 143                   Rodents GENlog     86 -0.076     -0.177 0.100   0.074
## 144                 Ruminants GENlog     15  0.003      0.036 0.050   0.185
## 145                   Insects GENlog     40 -0.030     -0.151 0.066   0.059
## 146 Disinfectant.QuatAmmonium GENlog     73 -0.051     -0.139 0.082   0.079
## 147              PressureWash GENlog      6  0.007      0.223 0.039   0.028
## 148        Obs.Entry.Measures GENlog     26  0.017      0.133 0.048   0.048
## 149                Obs.Shower GENlog     27 -0.019     -0.138 0.050   0.043
## 150              Obs.GI.signs GENlog      4  0.002      0.091 0.022   0.147
## 151            Obs.Resp.signs GENlog     15  0.005      0.062 0.056   0.203
## 152              Carbadox.dum GENlog      1 -0.001     -0.136 0.010   0.000
## 153              Tiamulin.dum GENlog     27 -0.012     -0.091 0.036   0.050
## 154                   CTC.dum GENlog    128 -0.071     -0.110 0.078   0.071
## 155            Lincomycin.dum GENlog     58 -0.087     -0.299 0.149   0.115
## 156             Roxarsone.dum GENlog     39 -0.029     -0.147 0.075   0.108
## 157         Virginiamycin.dum GENlog     10 -0.007     -0.131 0.038   0.115
## 158                 Pen.G.dum GENlog      2  0.003      0.296 0.030   0.035
## 159          Enrofloxacin.dum GENlog      4 -0.004     -0.221 0.032   0.050
## 160             Ceftiofur.dum GENlog     29 -0.026     -0.177 0.080   0.133
## 161           Site.Flow.Batch GENlog      2 -0.003     -0.269 0.028   0.105
## 162           Barn.Flow.Batch GENlog    107 -0.081     -0.150 0.087   0.060
## 163           Room.Flow.Batch GENlog      8  0.005      0.127 0.030   0.088
## 164              LabConf.Resp GENlog     21 -0.009     -0.086 0.035   0.072
## 165               Source.Feed GENlog     40 -0.069     -0.341 0.152   0.146
## 166              Source.Water GENlog     16  0.000      0.001 0.051   0.184
## 167             Source.Lagoon GENlog     29  0.024      0.165 0.061   0.048
## 168          Source.Fecal.Sow GENlog    144  0.175      0.242 0.138   0.100
## 169       Source.Swab.Surface GENlog     21 -0.008     -0.077 0.070   0.209
## 170            SampleStage.N1 GENlog      1 -0.001     -0.113 0.008   0.000
## 171            SampleStage.N2 GENlog    152 -0.155     -0.203 0.103   0.064
## 172            SampleStage.F1 GENlog     43  0.023      0.104 0.049   0.049
## 173            SampleStage.F2 GENlog    135 -0.107     -0.157 0.089   0.061
## 174               X23S_A2075G TETlog     72  0.184      0.509 0.387   0.499
## 175            X50S_L22_A103V TETlog    195  0.877      0.895 0.335   0.313
## 176                   aadE_Cc TETlog     22  0.053      0.476 0.160   0.177
## 177                      acr3 TETlog    135  0.321      0.474 0.317   0.276
## 178              aph.3pr_IIIa TETlog    124  0.305      0.490 0.358   0.339
## 179                blaOXA_193 TETlog    106 -0.301     -0.564 0.301   0.145
## 180                blaOXA_489 TETlog    110 -0.198     -0.358 0.412   0.501
## 181                blaOXA_578 TETlog    162 -0.665     -0.817 0.421   0.305
## 182                 gyrA_T86I TETlog     82 -0.083     -0.201 0.148   0.173
## 183                 rpsL_K43R TETlog    132  0.802      1.209 0.715   0.526
## 184                     tet.O TETlog     90  0.051      0.112 0.471   0.698
## 185                Pig.Mixing TETlog     51 -0.082     -0.320 0.194   0.266
## 186                   Rodents TETlog     38 -0.120     -0.630 0.262   0.193
## 187                 Ruminants TETlog    182  0.624      0.682 0.398   0.365
## 188                   Insects TETlog    151 -0.400     -0.527 0.305   0.236
## 189 Disinfectant.QuatAmmonium TETlog     90  0.263      0.582 0.491   0.591
## 190              PressureWash TETlog    194 -0.886     -0.909 0.459   0.442
## 191        Obs.Entry.Measures TETlog    198 -1.034     -1.039 0.450   0.445
## 192                Obs.Shower TETlog    177 -0.390     -0.439 0.205   0.160
## 193              Obs.GI.signs TETlog    120 -0.294     -0.487 0.307   0.249
## 194            Obs.Resp.signs TETlog     75  0.204      0.540 0.336   0.343
## 195              Carbadox.dum TETlog      3  0.009      0.577 0.074   0.223
## 196              Tiamulin.dum TETlog     15  0.039      0.524 0.224   0.659
## 197            Lincomycin.dum TETlog    127  0.925      1.450 0.887   0.685
## 198                   OTC.dum TETlog     10  0.010      0.203 0.070   0.255
## 199             Roxarsone.dum TETlog     48  0.016      0.065 0.310   0.633
## 200         Virginiamycin.dum TETlog     74 -0.252     -0.678 0.452   0.511
## 201                 Pen.G.dum TETlog     31 -0.192     -1.231 0.525   0.705
## 202          Enrofloxacin.dum TETlog    168 -0.765     -0.906 0.582   0.522
## 203             Ceftiofur.dum TETlog     73  0.120      0.328 0.343   0.505
## 204           Site.Flow.Batch TETlog     12  0.001      0.019 0.165   0.699
## 205           Barn.Flow.Batch TETlog     29 -0.049     -0.335 0.144   0.216
## 206           Room.Flow.Batch TETlog     98 -0.191     -0.389 0.485   0.634
## 207              LabConf.Resp TETlog     35  0.048      0.272 0.155   0.277
## 208               Source.Feed TETlog    117  0.338      0.575 0.665   0.786
## 209              Source.Water TETlog    117  0.297      0.506 0.299   0.216
## 210             Source.Lagoon TETlog     70  0.042      0.119 0.256   0.423
## 211          Source.Fecal.Sow TETlog     92  0.239      0.516 0.364   0.379
## 212       Source.Swab.Surface TETlog     75  0.094      0.249 0.242   0.342
## 213            SampleStage.N1 TETlog      6 -0.002     -0.066 0.060   0.371
## 214            SampleStage.N2 TETlog     49  0.176      0.714 0.318   0.156
## 215            SampleStage.F1 TETlog    145  0.338      0.464 0.337   0.312
## 216            SampleStage.F2 TETlog     19 -0.004     -0.038 0.218   0.724
## 217               X23S_A2075G FFNlog    196 -0.559     -0.567 0.225   0.216
## 218            X50S_L22_A103V FFNlog     79 -0.138     -0.348 0.184   0.111
## 219                   aadE_Cc FFNlog     53  0.015      0.055 0.055   0.097
## 220                      acr3 FFNlog     53 -0.063     -0.238 0.122   0.120
## 221              aph.3pr_IIIa FFNlog     71 -0.076     -0.214 0.148   0.180
## 222                blaOXA_193 FFNlog     13 -0.005     -0.084 0.035   0.114
## 223                blaOXA_489 FFNlog     33  0.009      0.054 0.076   0.182
## 224                blaOXA_578 FFNlog     43 -0.007     -0.033 0.098   0.211
## 225                 gyrA_T86I FFNlog    165 -0.142     -0.171 0.093   0.074
## 226                 rpsL_K43R FFNlog     72 -0.149     -0.413 0.253   0.260
## 227                     tet.O FFNlog    123 -0.114     -0.184 0.117   0.095
## 228                Pig.Mixing FFNlog     31 -0.009     -0.058 0.058   0.138
## 229                   Rodents FFNlog     31 -0.023     -0.149 0.066   0.098
## 230                 Ruminants FFNlog     31 -0.041     -0.264 0.114   0.157
## 231                   Insects FFNlog     19 -0.009     -0.093 0.043   0.108
## 232 Disinfectant.QuatAmmonium FFNlog     22 -0.003     -0.023 0.075   0.230
## 233              PressureWash FFNlog      6 -0.001     -0.020 0.015   0.091
## 234        Obs.Entry.Measures FFNlog     27  0.026      0.189 0.073   0.092
## 235                Obs.Shower FFNlog     21  0.004      0.040 0.028   0.079
## 236              Obs.GI.signs FFNlog     38 -0.014     -0.075 0.052   0.098
## 237            Obs.Resp.signs FFNlog     60 -0.058     -0.193 0.121   0.152
## 238              Carbadox.dum FFNlog      3  0.000      0.031 0.012   0.118
## 239              Tiamulin.dum FFNlog     32 -0.019     -0.116 0.060   0.107
## 240                   CTC.dum FFNlog     78 -0.034     -0.088 0.076   0.101
## 241            Lincomycin.dum FFNlog     53 -0.056     -0.210 0.132   0.182
## 242                   OTC.dum FFNlog      8  0.000      0.000 0.033   0.173
## 243             Roxarsone.dum FFNlog     37  0.039      0.210 0.110   0.172
## 244         Virginiamycin.dum FFNlog     53 -0.014     -0.053 0.061   0.110
## 245                 Pen.G.dum FFNlog     21  0.048      0.455 0.147   0.134
## 246          Enrofloxacin.dum FFNlog     30  0.026      0.171 0.077   0.120
## 247             Ceftiofur.dum FFNlog     81  0.069      0.170 0.115   0.124
## 248           Site.Flow.Batch FFNlog      7  0.002      0.070 0.037   0.198
## 249           Barn.Flow.Batch FFNlog    110 -0.034     -0.062 0.095   0.121
## 250           Room.Flow.Batch FFNlog     18  0.013      0.147 0.058   0.137
## 251              LabConf.Resp FFNlog     30  0.014      0.091 0.058   0.124
## 252               Source.Feed FFNlog     32  0.007      0.043 0.110   0.274
## 253              Source.Water FFNlog    115  0.092      0.160 0.106   0.093
## 254             Source.Lagoon FFNlog     44 -0.017     -0.077 0.072   0.138
## 255          Source.Fecal.Sow FFNlog     16 -0.008     -0.105 0.058   0.181
## 256       Source.Swab.Surface FFNlog    110  0.085      0.153 0.098   0.084
## 257            SampleStage.N1 FFNlog     24  0.009      0.071 0.054   0.143
## 258            SampleStage.N2 FFNlog     37 -0.002     -0.012 0.064   0.149
## 259            SampleStage.F1 FFNlog     18 -0.015     -0.170 0.059   0.113
## 260            SampleStage.F2 FFNlog    126 -0.091     -0.143 0.093   0.078
## 261               X23S_A2075G NALlog     33 -0.023     -0.136 0.062   0.089
## 262            X50S_L22_A103V NALlog     88  0.085      0.193 0.115   0.095
## 263                   aadE_Cc NALlog     84  0.042      0.100 0.059   0.050
## 264                      acr3 NALlog      7  0.005      0.131 0.037   0.158
## 265              aph.3pr_IIIa NALlog    179  0.278      0.309 0.156   0.133
## 266                blaOXA_193 NALlog     56  0.036      0.128 0.065   0.058
## 267                blaOXA_489 NALlog     94 -0.075     -0.158 0.088   0.057
## 268                blaOXA_578 NALlog    125 -0.114     -0.182 0.114   0.092
## 269                 gyrA_T86I NALlog    199  3.638      3.638 0.095   0.095
## 270                 rpsL_K43R NALlog    107 -0.101     -0.187 0.143   0.148
## 271                     tet.O NALlog     26 -0.001     -0.009 0.063   0.178
## 272                Pig.Mixing NALlog     38  0.001      0.003 0.069   0.160
## 273                   Rodents NALlog     12 -0.005     -0.078 0.037   0.136
## 274                 Ruminants NALlog     39  0.029      0.150 0.080   0.122
## 275                   Insects NALlog     79 -0.043     -0.109 0.064   0.055
## 276 Disinfectant.QuatAmmonium NALlog     31  0.024      0.157 0.077   0.133
## 277              PressureWash NALlog     32  0.008      0.050 0.028   0.053
## 278        Obs.Entry.Measures NALlog     85 -0.055     -0.128 0.072   0.054
## 279                Obs.Shower NALlog     51 -0.025     -0.098 0.047   0.036
## 280              Obs.GI.signs NALlog      4  0.000     -0.023 0.016   0.127
## 281            Obs.Resp.signs NALlog     71  0.080      0.224 0.135   0.138
## 282              Carbadox.dum NALlog      5 -0.001     -0.047 0.008   0.020
## 283                   CTC.dum NALlog     13 -0.010     -0.160 0.044   0.081
## 284            Lincomycin.dum NALlog     95  0.102      0.213 0.139   0.129
## 285                   OTC.dum NALlog      6  0.002      0.059 0.012   0.040
## 286             Roxarsone.dum NALlog     44 -0.031     -0.140 0.066   0.066
## 287         Virginiamycin.dum NALlog      2 -0.001     -0.096 0.010   0.026
## 288                 Pen.G.dum NALlog     49 -0.014     -0.059 0.035   0.048
## 289          Enrofloxacin.dum NALlog     23 -0.009     -0.080 0.029   0.041
## 290             Ceftiofur.dum NALlog     63  0.071      0.225 0.113   0.073
## 291           Site.Flow.Batch NALlog     73 -0.047     -0.128 0.077   0.076
## 292           Barn.Flow.Batch NALlog     18 -0.008     -0.090 0.038   0.095
## 293           Room.Flow.Batch NALlog     51  0.038      0.147 0.075   0.076
## 294              LabConf.Resp NALlog     18  0.009      0.095 0.031   0.048
## 295               Source.Feed NALlog     59 -0.069     -0.233 0.154   0.205
## 296              Source.Water NALlog     63 -0.063     -0.198 0.096   0.046
## 297             Source.Lagoon NALlog     59 -0.069     -0.232 0.118   0.097
## 298          Source.Fecal.Sow NALlog     23  0.024      0.209 0.070   0.058
## 299       Source.Swab.Surface NALlog     16  0.000     -0.004 0.056   0.202
## 300            SampleStage.N1 NALlog     42  0.021      0.102 0.055   0.080
## 301            SampleStage.N2 NALlog     16 -0.006     -0.076 0.032   0.087
## 302            SampleStage.F1 NALlog     60  0.024      0.078 0.045   0.050
## 303            SampleStage.F2 NALlog     46 -0.030     -0.128 0.060   0.053
## 304               X23S_A2075G TELlog    197  1.197      1.209 0.407   0.391
## 305            X50S_L22_A103V TELlog    153  0.243      0.316 0.188   0.151
## 306                   aadE_Cc TELlog     82  0.059      0.143 0.116   0.144
## 307                      acr3 TELlog     68  0.077      0.226 0.163   0.211
## 308              aph.3pr_IIIa TELlog    132  0.225      0.339 0.201   0.148
## 309                blaOXA_193 TELlog    195 -0.545     -0.556 0.238   0.227
## 310                blaOXA_489 TELlog     95  0.046      0.097 0.281   0.401
## 311                blaOXA_578 TELlog     95  0.308      0.645 0.349   0.190
## 312                 gyrA_T86I TELlog    198 -0.534     -0.536 0.167   0.163
## 313                 rpsL_K43R TELlog     71  0.238      0.667 0.378   0.339
## 314                     tet.O TELlog     25  0.009      0.076 0.071   0.190
## 315                Pig.Mixing TELlog     69  0.062      0.177 0.100   0.092
## 316                   Rodents TELlog     86  0.189      0.438 0.258   0.213
## 317                 Ruminants TELlog    191  0.457      0.476 0.247   0.233
## 318                   Insects TELlog    107 -0.088     -0.164 0.124   0.127
## 319 Disinfectant.QuatAmmonium TELlog     39 -0.042     -0.214 0.103   0.131
## 320              PressureWash TELlog      8  0.000      0.005 0.041   0.217
## 321        Obs.Entry.Measures TELlog      5  0.000     -0.016 0.018   0.126
## 322                Obs.Shower TELlog     53 -0.049     -0.183 0.109   0.142
## 323              Obs.GI.signs TELlog     92 -0.061     -0.132 0.092   0.094
## 324            Obs.Resp.signs TELlog    165  0.370      0.446 0.316   0.294
## 325              Carbadox.dum TELlog      2 -0.002     -0.227 0.023   0.022
## 326              Tiamulin.dum TELlog    129 -0.090     -0.139 0.149   0.166
## 327                   CTC.dum TELlog     30 -0.034     -0.225 0.097   0.138
## 328            Lincomycin.dum TELlog     62  0.057      0.183 0.131   0.179
## 329                   OTC.dum TELlog     33 -0.040     -0.241 0.108   0.147
## 330             Roxarsone.dum TELlog     19 -0.001     -0.006 0.070   0.233
## 331         Virginiamycin.dum TELlog     28  0.008      0.058 0.082   0.215
## 332          Enrofloxacin.dum TELlog     14  0.002      0.022 0.050   0.194
## 333             Ceftiofur.dum TELlog     70  0.102      0.289 0.160   0.135
## 334           Site.Flow.Batch TELlog     15 -0.008     -0.102 0.051   0.162
## 335           Barn.Flow.Batch TELlog     30  0.026      0.170 0.068   0.081
## 336           Room.Flow.Batch TELlog     11 -0.009     -0.160 0.042   0.088
## 337              LabConf.Resp TELlog    107 -0.082     -0.152 0.125   0.136
## 338               Source.Feed TELlog     65  0.059      0.182 0.208   0.333
## 339              Source.Water TELlog     35  0.052      0.295 0.126   0.134
## 340             Source.Lagoon TELlog     51 -0.033     -0.130 0.095   0.153
## 341          Source.Fecal.Sow TELlog     46 -0.028     -0.122 0.073   0.110
## 342       Source.Swab.Surface TELlog    117  0.134      0.228 0.140   0.109
## 343            SampleStage.N1 TELlog     34  0.001      0.009 0.061   0.149
## 344            SampleStage.N2 TELlog    103 -0.118     -0.228 0.145   0.124
## 345            SampleStage.F1 TELlog     14  0.005      0.068 0.035   0.117
## 346            SampleStage.F2 TELlog     14  0.010      0.142 0.051   0.138
## 347               X23S_A2075G CLIlog    199  1.565      1.565 0.442   0.442
## 348            X50S_L22_A103V CLIlog     35  0.019      0.108 0.112   0.252
## 349                   aadE_Cc CLIlog     35  0.006      0.034 0.106   0.253
## 350                      acr3 CLIlog     48  0.025      0.102 0.146   0.286
## 351              aph.3pr_IIIa CLIlog     94  0.089      0.188 0.205   0.266
## 352                blaOXA_193 CLIlog    106 -0.234     -0.440 0.237   0.122
## 353                blaOXA_489 CLIlog    172  0.572      0.661 0.358   0.298
## 354                blaOXA_578 CLIlog    174  0.571      0.654 0.374   0.326
## 355                 gyrA_T86I CLIlog    199 -0.601     -0.601 0.154   0.154
## 356                 rpsL_K43R CLIlog     68  0.001      0.003 0.306   0.526
## 357                     tet.O CLIlog    191  0.486      0.507 0.192   0.168
## 358                Pig.Mixing CLIlog     30  0.003      0.021 0.046   0.119
## 359                   Rodents CLIlog     52  0.068      0.260 0.174   0.258
## 360                 Ruminants CLIlog    107  0.148      0.276 0.219   0.232
## 361                   Insects CLIlog     71  0.023      0.064 0.109   0.175
## 362 Disinfectant.QuatAmmonium CLIlog     60  0.071      0.236 0.145   0.177
## 363              PressureWash CLIlog     19 -0.012     -0.129 0.065   0.176
## 364        Obs.Entry.Measures CLIlog      7 -0.004     -0.128 0.042   0.201
## 365                Obs.Shower CLIlog     15 -0.004     -0.059 0.040   0.137
## 366              Obs.GI.signs CLIlog     21  0.010      0.094 0.067   0.188
## 367            Obs.Resp.signs CLIlog    194  0.659      0.676 0.361   0.349
## 368              Tiamulin.dum CLIlog    150 -0.158     -0.209 0.183   0.183
## 369                   CTC.dum CLIlog     39 -0.058     -0.298 0.139   0.164
## 370            Lincomycin.dum CLIlog     55  0.024      0.085 0.120   0.217
## 371                   OTC.dum CLIlog      6 -0.006     -0.215 0.041   0.106
## 372             Roxarsone.dum CLIlog     48  0.044      0.183 0.116   0.175
## 373         Virginiamycin.dum CLIlog     62 -0.005     -0.017 0.148   0.266
## 374                 Pen.G.dum CLIlog      3 -0.002     -0.126 0.019   0.111
## 375          Enrofloxacin.dum CLIlog     16  0.010      0.120 0.064   0.202
## 376             Ceftiofur.dum CLIlog     35 -0.005     -0.028 0.098   0.236
## 377           Site.Flow.Batch CLIlog     24  0.051      0.423 0.163   0.255
## 378           Barn.Flow.Batch CLIlog     56  0.031      0.109 0.076   0.109
## 379           Room.Flow.Batch CLIlog      2 -0.001     -0.087 0.019   0.231
## 380              LabConf.Resp CLIlog    138 -0.122     -0.176 0.165   0.173
## 381               Source.Feed CLIlog    110 -0.142     -0.257 0.279   0.334
## 382              Source.Water CLIlog     75 -0.035     -0.092 0.190   0.301
## 383             Source.Lagoon CLIlog    184 -0.332     -0.359 0.185   0.165
## 384          Source.Fecal.Sow CLIlog     96 -0.072     -0.150 0.121   0.136
## 385       Source.Swab.Surface CLIlog    110  0.150      0.271 0.183   0.165
## 386            SampleStage.N1 CLIlog     53  0.006      0.024 0.108   0.211
## 387            SampleStage.N2 CLIlog     43 -0.035     -0.160 0.095   0.149
## 388            SampleStage.F1 CLIlog     17  0.010      0.115 0.055   0.158
## 389            SampleStage.F2 CLIlog     21  0.013      0.120 0.075   0.205
##       B_50 CI_lower CI_upper n_na
## 1    5.950    4.451    7.284    0
## 2    0.000    0.000    1.069    0
## 3    0.000    0.000    1.103    0
## 4    0.000   -1.909    0.864    0
## 5    0.000   -1.225    0.532    0
## 6   -1.222   -1.965    0.000    0
## 7    1.471    0.000    3.180    0
## 8    1.945    0.000    3.646    0
## 9   -1.641   -2.500   -0.971    0
## 10   0.000   -1.864    0.452    0
## 11   1.051    0.000    2.018    0
## 12   0.000    0.000    1.017    0
## 13   0.000   -1.687    0.649    0
## 14   1.560    0.000    3.075    0
## 15  -0.074   -1.113    0.000    0
## 16   0.000   -1.169    0.923    0
## 17   0.000    0.000    0.000    0
## 18   0.000    0.000    0.860    0
## 19   0.000   -0.914    0.000    0
## 20   0.000   -0.902    0.000    0
## 21   2.171    0.000    3.893    0
## 22   0.000    0.000    0.000    0
## 23   0.000   -1.246    0.000    0
## 24   0.000    0.000    0.111    0
## 25   0.000   -0.238    1.712    0
## 26   0.000   -0.241    0.715    0
## 27   0.600    0.000    1.954    0
## 28   0.000    0.000    0.000    0
## 29   0.000    0.000    0.005    0
## 30   0.000    0.000    1.054    0
## 31   0.000   -0.992    0.503    0
## 32  -0.369   -1.885    0.000    0
## 33   0.885    0.000    1.524    0
## 34   0.000    0.000    0.000    0
## 35  -0.704   -1.546    0.000    0
## 36  -0.543   -2.253    0.840    0
## 37   0.000   -1.352    0.013    0
## 38  -1.002   -1.968    0.000    0
## 39  -0.479   -1.701    0.000    0
## 40   0.000   -1.156    0.491    0
## 41   0.000    0.000    1.212    0
## 42   0.000   -0.736    0.369    0
## 43   0.000    0.000    0.542    0
## 44   0.000   -0.533    0.188    0
## 45  -0.169   -0.512    0.000    0
## 46   0.246    0.000    0.529    0
## 47   0.000    0.000    0.069    0
## 48   0.402    0.136    0.712    0
## 49   0.127    0.000    0.412    0
## 50   0.000    0.000    0.200    0
## 51  -0.365   -0.661    0.000    0
## 52  -0.277   -0.570    0.000    0
## 53   6.214    5.839    6.580    0
## 54   0.000    0.000    0.068    0
## 55  -0.248   -0.599    0.000    0
## 56  -0.292   -0.602    0.000    0
## 57   0.000   -0.309    0.000    0
## 58   0.000   -0.003    0.359    0
## 59   0.000    0.000    0.000    0
## 60   0.000   -0.240    0.173    0
## 61   0.000    0.000    0.000    0
## 62   0.000   -0.321    0.091    0
## 63   0.000   -0.102    0.000    0
## 64   0.000   -0.056    0.000    0
## 65   0.000   -0.304    0.000    0
## 66   0.000    0.000    0.055    0
## 67   0.000    0.000    0.000    0
## 68   0.174    0.000    0.814    0
## 69   0.000    0.000    0.207    0
## 70   0.000   -0.193    0.000    0
## 71   0.000   -0.193    0.000    0
## 72  -0.144   -0.496    0.000    0
## 73   0.000   -0.218    0.000    0
## 74   0.000   -0.518    0.000    0
## 75   0.000    0.000    0.000    0
## 76  -0.176   -0.459    0.000    0
## 77   0.000    0.000    0.000    0
## 78   0.176    0.000    0.360    0
## 79   0.000   -0.337    0.374    0
## 80   0.000    0.000    0.109    0
## 81   0.000    0.000    0.347    0
## 82   0.000    0.000    0.299    0
## 83   0.115    0.000    0.392    0
## 84   0.000   -0.264    0.164    0
## 85   0.000    0.000    0.265    0
## 86   0.000    0.000    0.218    0
## 87   0.000   -0.194    0.000    0
## 88   3.859    2.778    4.896    0
## 89   0.000    0.000    0.888    0
## 90   0.000    0.000    0.998    0
## 91   0.000   -0.877    0.474    0
## 92   0.000   -0.391    0.614    0
## 93  -0.823   -1.446    0.000    0
## 94   0.811    0.000    1.954    0
## 95   1.231    0.000    2.425    0
## 96  -1.052   -1.579   -0.591    0
## 97   0.000   -0.700    0.667    0
## 98   0.667    0.000    1.226    0
## 99   0.000    0.000    0.557    0
## 100  0.000   -1.121    0.073    0
## 101  1.052    0.000    2.090    0
## 102  0.000   -0.630    0.091    0
## 103  0.000   -0.692    0.431    0
## 104  0.000   -0.167    0.000    0
## 105  0.000    0.000    0.382    0
## 106  0.000   -0.741    0.000    0
## 107  0.000   -0.560    0.000    0
## 108  1.212    0.000    2.463    0
## 109 -0.046   -0.694    0.210    0
## 110  0.000    0.000    0.000    0
## 111  0.000    0.000    1.184    0
## 112  0.000   -0.366    0.003    0
## 113  0.601    0.000    1.356    0
## 114  0.000    0.000    0.000    0
## 115  0.000    0.000    0.000    0
## 116  0.000   -0.003    0.001    0
## 117  0.000   -0.368    0.541    0
## 118  0.000   -0.890    0.000    0
## 119  0.429    0.000    0.854    0
## 120  0.000    0.000    0.000    0
## 121 -0.320   -0.862    0.000    0
## 122  0.000   -1.153    0.502    0
## 123 -0.371   -1.045    0.000    0
## 124 -0.574   -1.089    0.000    0
## 125 -0.165   -0.841    0.000    0
## 126  0.000   -0.739    0.176    0
## 127  0.000    0.000    0.658    0
## 128  0.000   -0.330    0.000    0
## 129  0.000    0.000    0.184    0
## 130  0.000   -0.146    0.195    0
## 131 -0.223   -0.518    0.000    0
## 132  0.000   -0.434    0.000    0
## 133  0.000   -0.087    0.000    0
## 134  0.166    0.000    0.344    0
## 135  0.000   -0.474    0.000    0
## 136  0.000    0.000    0.283    0
## 137 -0.258   -0.478    0.000    0
## 138 -0.170   -0.421    0.000    0
## 139  0.000   -0.194    0.000    0
## 140 -0.132   -0.673    0.000    0
## 141  0.000   -0.175    0.248    0
## 142  0.000   -0.204    0.000    0
## 143  0.000   -0.278    0.000    0
## 144  0.000   -0.130    0.175    0
## 145  0.000   -0.196    0.000    0
## 146  0.000   -0.245    0.000    0
## 147  0.000    0.000    0.184    0
## 148  0.000    0.000    0.179    0
## 149  0.000   -0.166    0.000    0
## 150  0.000    0.000    0.000    0
## 151  0.000   -0.100    0.167    0
## 152  0.000    0.000    0.000    0
## 153  0.000   -0.134    0.000    0
## 154 -0.058   -0.244    0.000    0
## 155  0.000   -0.484    0.000    0
## 156  0.000   -0.239    0.000    0
## 157  0.000   -0.117    0.000    0
## 158  0.000    0.000    0.000    0
## 159  0.000    0.000    0.000    0
## 160  0.000   -0.272    0.000    0
## 161  0.000    0.000    0.000    0
## 162 -0.077   -0.274    0.000    0
## 163  0.000    0.000    0.064    0
## 164  0.000   -0.133    0.000    0
## 165  0.000   -0.512    0.000    0
## 166  0.000   -0.172    0.150    0
## 167  0.000    0.000    0.212    0
## 168  0.182    0.000    0.413    0
## 169  0.000   -0.213    0.153    0
## 170  0.000    0.000    0.000    0
## 171 -0.175   -0.313    0.000    0
## 172  0.000    0.000    0.161    0
## 173 -0.118   -0.276    0.000    0
## 174  0.000   -0.052    1.165    0
## 175  0.893    0.232    1.447    0
## 176  0.000    0.000    0.585    0
## 177  0.264    0.000    0.992    0
## 178  0.272   -0.375    0.989    0
## 179 -0.368   -0.821    0.000    0
## 180  0.000   -1.023    0.689    0
## 181 -0.708   -1.511    0.000    0
## 182  0.000   -0.471    0.008    0
## 183  0.851    0.000    2.060    0
## 184  0.000   -0.936    1.093    0
## 185  0.000   -0.650    0.000    0
## 186  0.000   -0.863    0.000    0
## 187  0.647    0.000    1.396    0
## 188 -0.409   -0.931    0.000    0
## 189  0.000   -0.420    1.350    0
## 190 -0.839   -1.911    0.000    0
## 191 -1.026   -1.802   -0.003    0
## 192 -0.425   -0.745    0.000    0
## 193 -0.247   -0.927    0.000    0
## 194  0.000    0.000    1.095    0
## 195  0.000    0.000    0.000    0
## 196  0.000   -0.002    0.715    0
## 197  0.806    0.000    2.470    0
## 198  0.000    0.000    0.278    0
## 199  0.000   -0.732    0.836    0
## 200  0.000   -1.133    0.000    0
## 201  0.000   -2.121    0.000    0
## 202 -0.732   -1.923    0.000    0
## 203  0.000   -0.564    0.916    0
## 204  0.000    0.000    0.243    0
## 205  0.000   -0.500    0.000    0
## 206  0.000   -1.926    0.385    0
## 207  0.000    0.000    0.530    0
## 208  0.000   -0.888    1.659    0
## 209  0.271    0.000    0.965    0
## 210  0.000   -0.530    0.634    0
## 211  0.000   -0.103    1.083    0
## 212  0.000   -0.537    0.583    0
## 213  0.000    0.000    0.000    0
## 214  0.000    0.000    0.904    0
## 215  0.355    0.000    0.886    0
## 216  0.000   -0.359    0.002    0
## 217 -0.590   -0.949   -0.035    0
## 218  0.000   -0.494    0.000    0
## 219  0.000   -0.091    0.164    0
## 220  0.000   -0.387    0.000    0
## 221  0.000   -0.439    0.122    0
## 222  0.000   -0.106    0.000    0
## 223  0.000   -0.163    0.248    0
## 224  0.000   -0.175    0.239    0
## 225 -0.150   -0.314    0.000    0
## 226  0.000   -0.783    0.000    0
## 227 -0.090   -0.365    0.000    0
## 228  0.000   -0.196    0.093    0
## 229  0.000   -0.253    0.000    0
## 230  0.000   -0.430    0.000    0
## 231  0.000   -0.139    0.000    0
## 232  0.000   -0.196    0.199    0
## 233  0.000    0.000    0.000    0
## 234  0.000    0.000    0.247    0
## 235  0.000   -0.002    0.084    0
## 236  0.000   -0.159    0.065    0
## 237  0.000   -0.411    0.000    0
## 238  0.000    0.000    0.000    0
## 239  0.000   -0.225    0.000    0
## 240  0.000   -0.246    0.085    0
## 241  0.000   -0.398    0.069    0
## 242  0.000    0.000    0.000    0
## 243  0.000    0.000    0.383    0
## 244  0.000   -0.156    0.116    0
## 245  0.000    0.000    0.589    0
## 246  0.000    0.000    0.302    0
## 247  0.000    0.000    0.354    0
## 248  0.000    0.000    0.001    0
## 249  0.000   -0.226    0.195    0
## 250  0.000    0.000    0.218    0
## 251  0.000   -0.073    0.188    0
## 252  0.000   -0.253    0.288    0
## 253  0.089    0.000    0.322    0
## 254  0.000   -0.204    0.113    0
## 255  0.000   -0.190    0.003    0
## 256  0.088    0.000    0.259    0
## 257  0.000   -0.003    0.141    0
## 258  0.000   -0.173    0.138    0
## 259  0.000   -0.167    0.000    0
## 260 -0.081   -0.286    0.000    0
## 261  0.000   -0.209    0.000    0
## 262  0.000    0.000    0.351    0
## 263  0.000    0.000    0.185    0
## 264  0.000    0.000    0.114    0
## 265  0.276    0.000    0.589    0
## 266  0.000    0.000    0.210    0
## 267  0.000   -0.253    0.000    0
## 268 -0.099   -0.343    0.000    0
## 269  3.637    3.467    3.829    0
## 270 -0.035   -0.455    0.009    0
## 271  0.000   -0.145    0.187    0
## 272  0.000   -0.169    0.197    0
## 273  0.000   -0.127    0.000    0
## 274  0.000    0.000    0.261    0
## 275  0.000   -0.187    0.000    0
## 276  0.000    0.000    0.283    0
## 277  0.000   -0.010    0.093    0
## 278  0.000   -0.212    0.000    0
## 279  0.000   -0.146    0.000    0
## 280  0.000    0.000    0.000    0
## 281  0.000    0.000    0.372    0
## 282  0.000   -0.001    0.000    0
## 283  0.000   -0.164    0.000    0
## 284  0.000    0.000    0.438    0
## 285  0.000    0.000    0.033    0
## 286  0.000   -0.214    0.000    0
## 287  0.000    0.000    0.000    0
## 288  0.000   -0.108    0.000    0
## 289  0.000   -0.098    0.000    0
## 290  0.000    0.000    0.349    0
## 291  0.000   -0.251    0.000    0
## 292  0.000   -0.157    0.001    0
## 293  0.000    0.000    0.241    0
## 294  0.000    0.000    0.107    0
## 295  0.000   -0.432    0.000    0
## 296  0.000   -0.270    0.000    0
## 297  0.000   -0.370    0.000    0
## 298  0.000    0.000    0.250    0
## 299  0.000   -0.202    0.130    0
## 300  0.000    0.000    0.181    0
## 301  0.000   -0.118    0.000    0
## 302  0.000    0.000    0.146    0
## 303  0.000   -0.176    0.000    0
## 304  1.271    0.286    1.814    0
## 305  0.258    0.000    0.623    0
## 306  0.000   -0.038    0.393    0
## 307  0.000   -0.096    0.516    0
## 308  0.241    0.000    0.632    0
## 309 -0.581   -0.919   -0.067    0
## 310  0.000   -0.568    0.620    0
## 311  0.000    0.000    0.963    0
## 312 -0.538   -0.871   -0.217    0
## 313  0.000    0.000    1.204    0
## 314  0.000    0.000    0.156    0
## 315  0.000    0.000    0.334    0
## 316  0.000    0.000    0.780    0
## 317  0.461    0.000    0.975    0
## 318 -0.009   -0.374    0.043    0
## 319  0.000   -0.348    0.000    0
## 320  0.000    0.000    0.000    0
## 321  0.000    0.000    0.000    0
## 322  0.000   -0.365    0.000    0
## 323  0.000   -0.268    0.039    0
## 324  0.332    0.000    0.947    0
## 325  0.000    0.000    0.000    0
## 326 -0.040   -0.413    0.217    0
## 327  0.000   -0.342    0.000    0
## 328  0.000   -0.108    0.432    0
## 329  0.000   -0.338    0.000    0
## 330  0.000   -0.074    0.105    0
## 331  0.000   -0.153    0.256    0
## 332  0.000   -0.006    0.106    0
## 333  0.000    0.000    0.488    0
## 334  0.000   -0.117    0.000    0
## 335  0.000    0.000    0.230    0
## 336  0.000   -0.152    0.000    0
## 337  0.000   -0.386    0.070    0
## 338  0.000   -0.242    0.739    0
## 339  0.000    0.000    0.464    0
## 340  0.000   -0.275    0.054    0
## 341  0.000   -0.247    0.000    0
## 342  0.107    0.000    0.421    0
## 343  0.000   -0.151    0.137    0
## 344 -0.046   -0.445    0.000    0
## 345  0.000    0.000    0.113    0
## 346  0.000    0.000    0.121    0
## 347  1.616    0.641    2.311    0
## 348  0.000   -0.116    0.326    0
## 349  0.000   -0.265    0.291    0
## 350  0.000   -0.212    0.478    0
## 351  0.000   -0.237    0.636    0
## 352 -0.254   -0.622    0.000    0
## 353  0.556    0.000    1.322    0
## 354  0.578    0.000    1.322    0
## 355 -0.607   -0.883   -0.311    0
## 356  0.000   -0.704    0.769    0
## 357  0.494    0.000    0.849    0
## 358  0.000   -0.084    0.108    0
## 359  0.000   -0.095    0.553    0
## 360  0.000   -0.103    0.628    0
## 361  0.000   -0.185    0.284    0
## 362  0.000    0.000    0.443    0
## 363  0.000   -0.201    0.000    0
## 364  0.000   -0.002    0.000    0
## 365  0.000   -0.113    0.000    0
## 366  0.000   -0.089    0.283    0
## 367  0.649    0.000    1.283    0
## 368 -0.142   -0.466    0.211    0
## 369  0.000   -0.490    0.000    0
## 370  0.000   -0.200    0.357    0
## 371  0.000   -0.046    0.000    0
## 372  0.000   -0.001    0.373    0
## 373  0.000   -0.310    0.352    0
## 374  0.000    0.000    0.000    0
## 375  0.000    0.000    0.188    0
## 376  0.000   -0.249    0.201    0
## 377  0.000    0.000    0.642    0
## 378  0.000   -0.038    0.239    0
## 379  0.000    0.000    0.000    0
## 380 -0.092   -0.479    0.136    0
## 381  0.000   -0.718    0.370    0
## 382  0.000   -0.437    0.409    0
## 383 -0.326   -0.654    0.000    0
## 384  0.000   -0.382    0.092    0
## 385  0.103   -0.070    0.548    0
## 386  0.000   -0.249    0.241    0
## 387  0.000   -0.397    0.000    0
## 388  0.000    0.000    0.094    0
## 389  0.000   -0.003    0.210    0
## 
## 
## OMEGA EDGES:
##        Y1     Y2 n_non0 Omega_bar SE_Omega Omega_bar_non0 SE_Omega_non0
## 1  AZIlog CIPlog    192    -0.041    0.021         -0.042         0.020
## 2  AZIlog ERYlog    199     0.824    0.028          0.824         0.028
## 3  AZIlog GENlog    116    -0.019    0.023         -0.032         0.022
## 4  AZIlog TETlog     94    -0.011    0.021         -0.024         0.025
## 5  AZIlog FFNlog    199    -0.216    0.025         -0.216         0.025
## 6  AZIlog NALlog    116    -0.008    0.011         -0.013         0.012
## 7  AZIlog TELlog    199    -0.141    0.062         -0.141         0.062
## 8  AZIlog CLIlog    199     0.107    0.058          0.107         0.058
## 9  CIPlog ERYlog      4     0.001    0.009          0.055         0.038
## 10 CIPlog GENlog    185     0.049    0.046          0.052         0.046
## 11 CIPlog TETlog    196     0.091    0.048          0.093         0.047
## 12 CIPlog FFNlog    199     0.229    0.047          0.229         0.047
## 13 CIPlog NALlog    199     0.630    0.052          0.630         0.052
## 14 CIPlog TELlog    199     0.115    0.033          0.115         0.033
## 15 CIPlog CLIlog     66    -0.012    0.024         -0.037         0.029
## 16 ERYlog GENlog      3     0.000    0.003          0.017         0.024
## 17 ERYlog TETlog     75     0.014    0.026          0.037         0.031
## 18 ERYlog FFNlog      0     0.000    0.000            NaN            NA
## 19 ERYlog NALlog      8     0.000    0.003         -0.006         0.015
## 20 ERYlog TELlog    199     0.438    0.037          0.438         0.037
## 21 ERYlog CLIlog    199     0.201    0.068          0.201         0.068
## 22 GENlog TETlog    199     0.161    0.051          0.161         0.051
## 23 GENlog FFNlog    198     0.169    0.050          0.170         0.049
## 24 GENlog NALlog    195     0.133    0.050          0.136         0.047
## 25 GENlog TELlog    197     0.069    0.043          0.070         0.043
## 26 GENlog CLIlog    120     0.038    0.045          0.062         0.043
## 27 TETlog FFNlog    199     0.338    0.043          0.338         0.043
## 28 TETlog NALlog    159    -0.047    0.048         -0.059         0.047
## 29 TETlog TELlog    199     0.192    0.040          0.192         0.040
## 30 TETlog CLIlog    199    -0.168    0.047         -0.168         0.047
## 31 FFNlog NALlog    101    -0.014    0.032         -0.028         0.041
## 32 FFNlog TELlog    199     0.341    0.044          0.341         0.044
## 33 FFNlog CLIlog     12     0.003    0.013          0.049         0.029
## 34 NALlog TELlog     65    -0.009    0.019         -0.028         0.025
## 35 NALlog CLIlog     46    -0.006    0.014         -0.026         0.019
## 36 TELlog CLIlog    199     0.329    0.041          0.329         0.041
##    Omega_50 CI_lower CI_upper n_na
## 1    -0.042   -0.079    0.000    0
## 2     0.825    0.760    0.873    0
## 3    -0.007   -0.076    0.000    0
## 4     0.000   -0.064    0.001    0
## 5    -0.216   -0.267   -0.164    0
## 6    -0.003   -0.033    0.009    0
## 7    -0.138   -0.268   -0.025    0
## 8     0.097    0.001    0.220    0
## 9     0.000    0.000    0.000    0
## 10    0.044   -0.025    0.139    0
## 11    0.088    0.004    0.196    0
## 12    0.230    0.143    0.325    0
## 13    0.633    0.533    0.717    0
## 14    0.114    0.055    0.185    0
## 15    0.000   -0.080    0.000    0
## 16    0.000    0.000    0.000    0
## 17    0.000    0.000    0.096    0
## 18    0.000    0.000    0.000    0
## 19    0.000   -0.003    0.000    0
## 20    0.437    0.372    0.519    0
## 21    0.211    0.060    0.325    0
## 22    0.161    0.070    0.256    0
## 23    0.169    0.059    0.254    0
## 24    0.138    0.019    0.223    0
## 25    0.072   -0.014    0.148    0
## 26    0.014    0.000    0.142    0
## 27    0.340    0.243    0.410    0
## 28   -0.032   -0.150    0.000    0
## 29    0.196    0.123    0.274    0
## 30   -0.169   -0.260   -0.075    0
## 31    0.000   -0.092    0.029    0
## 32    0.344    0.252    0.415    0
## 33    0.000    0.000    0.059    0
## 34    0.000   -0.077    0.000    0
## 35    0.000   -0.048    0.000    0
## 36    0.332    0.247    0.410    0
```

### ABF Farms

```
Y <- abf.y_cent
```

#### Environmental

```
X <- abf.env_cent

model.cg = l1ML_Main(
  Y = Y, 
  X = X, 
  initializer = 'Lasso', 
  screening = T,
  ss = T,
  alpha = 0.05,
  nboot = 20
)
```

```
## Step 0: screening is on, proceed with debiased Lasso in conjunction with BH correction, the cut-off for p-values is set at 0.05 .
## Step 1: Penalized LS initialization with Lasso .
## Step 2: Alternate update.
```

```
## Warning in l1ML_Main(Y = Y, X = X, initializer = "Lasso", screening = T, : l1ML_Main(): alternate update is broken manually at iteration =50
```

```
## Step 3: Refitting B Matrix.
## Step 4: Stability selection with a total number of 20 bootstrapped samples ...25%...50%...75%...Done!
## Step 5: Refitting with weighted glasso ...Done.
## Returns: B.est, Theta.est, BICvalue.
```

```
model.cg_formatted <- format.chain.graph(model.cg, x.names = colnames(X), y.names = colnames(Y))
print(display.chain.graph(model.cg_formatted))
```

```
## 
## Lambda: 
## Edges: 57 (32%)
## 
## Beta Matrix:
##                            AZIlog CIPlog ERYlog GENlog TETlog FFNlog NALlog
## Birds                     -1.158  0.000 -0.912  0.000 -2.401  0.000  0.000
## Chicks.Ducks               0.000  0.000  0.978 -0.261  1.226  0.000  0.000
## Ruminants                  0.000  0.000  0.000  0.000  0.000  0.000  0.000
## Visitors.Day               0.000 -0.260  0.000 -0.355  0.000 -0.146  0.000
## Disinfectant.Clorox        0.000  0.000 -0.469  0.000 -0.815  0.232  0.000
## Disinfectant.QuatAmmonium  0.000  0.000 -0.431  0.000  0.833 -0.422  0.000
## Obs.Toilet                 1.419  0.000  0.000  0.000  0.000  0.000  0.000
## Obs.GI.signs               0.000  0.000  0.000  0.000  0.000 -0.041 -0.069
## CuExp                      1.911  0.230  0.794  0.000  2.100  0.534  0.000
## ZnExp                      2.895  0.248  2.070  0.000  0.997  0.000  0.000
## FeedSource.Farm            4.267  0.000  2.491  0.000  0.000  0.000 -0.199
## WaterQC                    0.000  0.000  0.000  0.000  0.000 -0.338  0.000
## Source.Water               0.000  0.000  0.000  0.357 -1.044  0.000  0.000
## Source.Soil                1.272  0.000  0.414  0.000  0.000  0.000  0.172
## Source.Fecal.Sow          -0.338  0.000  0.000  0.000  0.621  0.000  0.000
## Source.Swab.Surface        0.000  0.000  0.000  0.000  0.000  0.000  0.000
## SampleStage.N1             0.000  0.005  0.000  0.000  0.000  0.000  0.000
## SampleStage.N2             0.000  0.000  0.000  0.000 -1.422  0.000  0.000
## SampleStage.F1             0.000 -0.183  0.000  0.000  0.000  0.000  0.000
## SampleStage.F2             0.000 -0.142  0.000  0.000  0.000  0.000  0.000
##                           TELlog CLIlog
## Birds                      0.000 -0.164
## Chicks.Ducks              -0.211  0.000
## Ruminants                  0.000  0.000
## Visitors.Day               0.000  0.000
## Disinfectant.Clorox        0.000  0.204
## Disinfectant.QuatAmmonium  1.024  0.274
## Obs.Toilet                 0.000 -0.572
## Obs.GI.signs               0.000  0.000
## CuExp                      0.732 -0.750
## ZnExp                      0.557  0.000
## FeedSource.Farm            1.457  1.183
## WaterQC                    0.000  0.000
## Source.Water              -0.296  0.000
## Source.Soil                0.000  0.095
## Source.Fecal.Sow          -0.463 -0.407
## Source.Swab.Surface        0.000  0.000
## SampleStage.N1             0.000  0.000
## SampleStage.N2             0.000  0.000
## SampleStage.F1             0.000  0.000
## SampleStage.F2             0.000  0.000
## 
## 
## Rho: 
## Edges: 27 (75%)
## 
## Omega Matrix: 
##        AZIlog CIPlog ERYlog GENlog TETlog FFNlog NALlog TELlog CLIlog
## AZIlog  0.000 -0.037  0.806 -0.044  0.134 -0.219  0.000 -0.273  0.232
## CIPlog -0.037  0.000  0.000  0.041  0.010  0.116  0.797  0.033  0.000
## ERYlog  0.806  0.000  0.000  0.000 -0.073  0.000  0.000  0.478  0.130
## GENlog -0.044  0.041  0.000  0.000 -0.017  0.418  0.067  0.000  0.153
## TETlog  0.133  0.010 -0.073 -0.017  0.000  0.086  0.076  0.541 -0.336
## FFNlog -0.219  0.116  0.000  0.418  0.086  0.000  0.000  0.131  0.074
## NALlog  0.000  0.797  0.000  0.066  0.077  0.000  0.000  0.000  0.018
## TELlog -0.273  0.033  0.478  0.000  0.541  0.131  0.000  0.000  0.422
## CLIlog  0.232  0.000  0.130  0.153 -0.336  0.074  0.018  0.422  0.000
## 
## 
## NULL
```

```
R2.tab <- ChainGraph_R2_v4(
  X, 
  Y, 
  Beta = model.cg_formatted$B.est, 
  Omega = model.cg_formatted$Omega.est
)

print(R2.tab)
```

```
##          Y
## AZIlog AZI
## CIPlog CIP
## ERYlog ERY
## GENlog GEN
## TETlog TET
## FFNlog FFN
## NALlog NAL
## TELlog TEL
## CLIlog CLI
##                                                                                                                                    X_set
## AZIlog                                                   Birds, Obs.Toilet, CuExp, ZnExp, FeedSource.Farm, Source.Soil, Source.Fecal.Sow
## CIPlog                                                        Visitors.Day, CuExp, ZnExp, SampleStage.N1, SampleStage.F1, SampleStage.F2
## ERYlog                   Birds, Chicks.Ducks, Disinfectant.Clorox, Disinfectant.QuatAmmonium, CuExp, ZnExp, FeedSource.Farm, Source.Soil
## GENlog                                                                                          Chicks.Ducks, Visitors.Day, Source.Water
## TETlog Birds, Chicks.Ducks, Disinfectant.Clorox, Disinfectant.QuatAmmonium, CuExp, ZnExp, Source.Water, Source.Fecal.Sow, SampleStage.N2
## FFNlog                                        Visitors.Day, Disinfectant.Clorox, Disinfectant.QuatAmmonium, Obs.GI.signs, CuExp, WaterQC
## NALlog                                                                                        Obs.GI.signs, FeedSource.Farm, Source.Soil
## TELlog                            Chicks.Ducks, Disinfectant.QuatAmmonium, CuExp, ZnExp, FeedSource.Farm, Source.Water, Source.Fecal.Sow
## CLIlog          Birds, Disinfectant.Clorox, Disinfectant.QuatAmmonium, Obs.Toilet, CuExp, FeedSource.Farm, Source.Soil, Source.Fecal.Sow
##               R2_X                                  Y_set   R2_e_Yj     R2_Yj
## AZIlog  0.33620693      CIP, ERY, GEN, TET, FFN, TEL, CLI 0.9431795 0.6260760
## CIPlog  0.06077242           AZI, GEN, TET, FFN, NAL, TEL 0.7322077 0.6877096
## ERYlog  0.30704846                     AZI, TET, TEL, CLI 0.9610427 0.6659561
## GENlog  0.22755143           AZI, CIP, TET, FFN, NAL, CLI 0.3629578 0.2803663
## TETlog  0.12122175 AZI, CIP, ERY, GEN, FFN, NAL, TEL, CLI 0.5879619 0.5166881
## FFNlog  0.14911334           AZI, CIP, GEN, TET, TEL, CLI 0.4579559 0.3896685
## NALlog -0.03224444                     CIP, GEN, TET, CLI 0.7109878 0.7339132
## TELlog  0.21658181           AZI, CIP, ERY, TET, FFN, CLI 0.8992974 0.7045259
## CLIlog  0.10837633      AZI, ERY, GEN, TET, FFN, NAL, TEL 0.8732326 0.7785949
##         R2_total
## AZIlog 0.9622830
## CIPlog 0.7484821
## ERYlog 0.9730045
## GENlog 0.5079177
## TETlog 0.6379099
## FFNlog 0.5387819
## NALlog 0.7016688
## TELlog 0.9211078
## CLIlog 0.8869712
```

#####Bootstrap

```
#cl <- parallel::makeCluster(n_cl)
#doParallel::registerDoParallel(cl)

model.cg_bs <- cg.bootstrap(
  Y = Y, 
  X = X, 
  b = B,
  n_b = n_b_abf,
  lambda = model.cg$lambda, 
  rho = model.cg$rho,
  initializer = 'Lasso', 
  screening = T,
  alpha = 0.05,
  nboot = 20,
  cluster = cl,
  seed.val = seeds[4]
)
```

```
## Starting parallel bootstrap at 2025-01-10 21:51:37.222005
```

```
## Ending bootstrap at 2025-01-10 22:01:38.812347
```

```
#stopCluster(cl)

summary.cg_bootstrap_results(model.cg_bs)
```

```
## Warning in summary.cg_bootstrap_results(model.cg_bs): Target number of
## subsamples (b = 200) exceeds available valid subsamples (b = 197).
```

```
## SAMPLE SIZES
## Original:  399 
## Subsample size:  200 
## 
## BOOTSTRAPS
## Total fit subsamples:  200 
## Subsamples for summary stats: 197 
## Subsamples that returned NAs: 3 
## 
## PENALTIES
## Lambda:  0.0866493 
## Rho:  0.07420801 
## 
## CI alpha: 95 %
## 
## BETA EDGES:
##                             X      Y n_non0  B_bar B_bar_non0  SE_B SE_non0
## 1                       Birds AZIlog    177 -1.385     -1.542 1.160   1.121
## 2                Chicks.Ducks AZIlog     72  0.716      1.958 1.794   2.533
## 3                   Ruminants AZIlog      6 -0.050     -1.643 0.363   1.428
## 4                Visitors.Day AZIlog      5  0.040      1.570 0.255   0.420
## 5         Disinfectant.Clorox AZIlog    156 -0.814     -1.028 0.896   0.891
## 6   Disinfectant.QuatAmmonium AZIlog     99 -1.001     -1.991 1.260   1.088
## 7                  Obs.Toilet AZIlog    118  1.433      2.392 1.362   0.892
## 8                Obs.GI.signs AZIlog     33  0.212      1.267 0.535   0.610
## 9                       CuExp AZIlog    148  1.110      1.478 1.045   0.954
## 10                      ZnExp AZIlog    108  1.960      3.576 1.909   0.920
## 11            FeedSource.Farm AZIlog    190  4.239      4.395 1.582   1.380
## 12               Source.Water AZIlog    121 -0.388     -0.631 0.907   1.090
## 13                Source.Soil AZIlog    167  1.085      1.280 0.990   0.952
## 14           Source.Fecal.Sow AZIlog    137 -0.074     -0.106 1.147   1.376
## 15        Source.Swab.Surface AZIlog    116 -0.463     -0.786 0.981   1.176
## 16             SampleStage.N1 AZIlog     62  0.362      1.151 0.716   0.852
## 17             SampleStage.N2 AZIlog     15  0.078      1.018 0.529   1.703
## 18             SampleStage.F1 AZIlog     38  0.264      1.367 0.711   1.064
## 19             SampleStage.F2 AZIlog     29  0.183      1.243 0.579   0.990
## 20                      Birds CIPlog     46  0.033      0.141 0.159   0.307
## 21               Chicks.Ducks CIPlog    102 -0.255     -0.493 0.347   0.339
## 22                  Ruminants CIPlog     30  0.024      0.156 0.129   0.302
## 23               Visitors.Day CIPlog     89 -0.125     -0.277 0.154   0.099
## 24        Disinfectant.Clorox CIPlog     65  0.061      0.186 0.191   0.297
## 25  Disinfectant.QuatAmmonium CIPlog     94 -0.084     -0.176 0.232   0.312
## 26                 Obs.Toilet CIPlog      8 -0.003     -0.075 0.078   0.403
## 27               Obs.GI.signs CIPlog     21  0.021      0.200 0.109   0.283
## 28                      CuExp CIPlog    132  0.245      0.366 0.255   0.229
## 29                      ZnExp CIPlog     77  0.083      0.211 0.179   0.235
## 30            FeedSource.Farm CIPlog     60  0.107      0.351 0.182   0.153
## 31                    WaterQC CIPlog     36 -0.048     -0.263 0.110   0.099
## 32               Source.Water CIPlog     63  0.067      0.210 0.121   0.124
## 33                Source.Soil CIPlog     30 -0.005     -0.031 0.089   0.230
## 34           Source.Fecal.Sow CIPlog    114  0.172      0.297 0.604   0.771
## 35        Source.Swab.Surface CIPlog     80 -0.059     -0.145 0.133   0.177
## 36             SampleStage.N1 CIPlog     56  0.004      0.016 0.068   0.128
## 37             SampleStage.N2 CIPlog     77 -0.063     -0.161 0.112   0.128
## 38             SampleStage.F1 CIPlog    119 -0.141     -0.233 0.143   0.110
## 39             SampleStage.F2 CIPlog     95 -0.115     -0.238 0.145   0.119
## 40                      Birds ERYlog    167 -0.537     -0.634 0.626   0.633
## 41               Chicks.Ducks ERYlog    121  0.919      1.496 1.183   1.190
## 42                  Ruminants ERYlog      4 -0.026     -1.279 0.190   0.477
## 43               Visitors.Day ERYlog      3  0.011      0.752 0.107   0.536
## 44        Disinfectant.Clorox ERYlog    172 -0.709     -0.812 0.556   0.520
## 45  Disinfectant.QuatAmmonium ERYlog    153 -0.949     -1.222 0.824   0.735
## 46                 Obs.Toilet ERYlog     63  0.528      1.650 0.826   0.523
## 47               Obs.GI.signs ERYlog     60  0.207      0.679 0.397   0.445
## 48                      CuExp ERYlog    146  0.743      1.002 0.753   0.711
## 49                      ZnExp ERYlog    172  1.599      1.831 1.308   1.238
## 50            FeedSource.Farm ERYlog    174  2.614      2.960 1.234   0.834
## 51                    WaterQC ERYlog      1 -0.001     -0.141 0.010   0.000
## 52               Source.Water ERYlog    116 -0.133     -0.225 0.548   0.701
## 53                Source.Soil ERYlog    151  0.533      0.695 0.575   0.564
## 54           Source.Fecal.Sow ERYlog    142  0.156      0.216 0.635   0.740
## 55        Source.Swab.Surface ERYlog    109 -0.239     -0.431 0.580   0.725
## 56             SampleStage.N1 ERYlog      6 -0.006     -0.205 0.076   0.419
## 57             SampleStage.N2 ERYlog     13 -0.025     -0.384 0.129   0.352
## 58             SampleStage.F1 ERYlog     13  0.034      0.511 0.200   0.626
## 59             SampleStage.F2 ERYlog      6 -0.003     -0.096 0.062   0.371
## 60                      Birds GENlog     48 -0.066     -0.269 0.133   0.136
## 61               Chicks.Ducks GENlog    109 -0.241     -0.436 0.267   0.209
## 62                  Ruminants GENlog     19  0.022      0.228 0.083   0.162
## 63               Visitors.Day GENlog    144 -0.252     -0.345 0.176   0.101
## 64        Disinfectant.Clorox GENlog     14  0.019      0.269 0.077   0.127
## 65  Disinfectant.QuatAmmonium GENlog     32  0.054      0.332 0.141   0.175
## 66                 Obs.Toilet GENlog     62  0.061      0.195 0.126   0.156
## 67               Obs.GI.signs GENlog     68  0.060      0.173 0.117   0.140
## 68                      CuExp GENlog     80 -0.126     -0.309 0.202   0.209
## 69                      ZnExp GENlog      3  0.002      0.114 0.041   0.382
## 70            FeedSource.Farm GENlog     57 -0.050     -0.171 0.123   0.178
## 71                    WaterQC GENlog      1  0.001      0.134 0.010   0.000
## 72               Source.Water GENlog    192  0.350      0.359 0.100   0.084
## 73                Source.Soil GENlog     62  0.093      0.297 0.167   0.168
## 74           Source.Fecal.Sow GENlog     30 -0.013     -0.084 0.180   0.462
## 75        Source.Swab.Surface GENlog     14 -0.001     -0.019 0.063   0.244
## 76             SampleStage.N1 GENlog     22 -0.020     -0.180 0.062   0.075
## 77             SampleStage.N2 GENlog     32  0.032      0.198 0.087   0.118
## 78             SampleStage.F1 GENlog      2  0.000     -0.039 0.019   0.263
## 79             SampleStage.F2 GENlog      1  0.000      0.095 0.007   0.000
## 80                      Birds TETlog    192 -2.529     -2.595 1.124   1.060
## 81               Chicks.Ducks TETlog    130  0.879      1.333 1.694   1.937
## 82                  Ruminants TETlog     50  0.218      0.858 0.542   0.783
## 83               Visitors.Day TETlog      6 -0.023     -0.771 0.169   0.650
## 84        Disinfectant.Clorox TETlog    129 -0.999     -1.525 1.039   0.919
## 85  Disinfectant.QuatAmmonium TETlog    101  0.098      0.191 0.805   1.119
## 86                 Obs.Toilet TETlog     39 -0.336     -1.699 0.747   0.707
## 87               Obs.GI.signs TETlog     39 -0.160     -0.810 0.723   1.468
## 88                      CuExp TETlog    136  1.546      2.240 1.342   1.025
## 89                      ZnExp TETlog    138  0.725      1.034 0.715   0.639
## 90            FeedSource.Farm TETlog     48  0.317      1.299 0.629   0.590
## 91                    WaterQC TETlog     34  0.041      0.239 0.430   1.024
## 92               Source.Water TETlog    176 -1.185     -1.326 0.717   0.622
## 93                Source.Soil TETlog    101  0.043      0.083 0.764   1.067
## 94           Source.Fecal.Sow TETlog    142  0.364      0.504 1.910   2.236
## 95        Source.Swab.Surface TETlog    102 -0.143     -0.276 0.569   0.769
## 96             SampleStage.N1 TETlog     57 -0.060     -0.209 0.421   0.767
## 97             SampleStage.N2 TETlog    149 -1.228     -1.624 0.801   0.450
## 98             SampleStage.F1 TETlog     43  0.218      0.998 0.501   0.613
## 99             SampleStage.F2 TETlog     61 -0.251     -0.810 0.526   0.666
## 100                     Birds FFNlog     23 -0.025     -0.215 0.122   0.301
## 101              Chicks.Ducks FFNlog     92  0.273      0.584 0.368   0.328
## 102                 Ruminants FFNlog     29 -0.019     -0.126 0.165   0.419
## 103              Visitors.Day FFNlog    140 -0.044     -0.061 0.200   0.236
## 104       Disinfectant.Clorox FFNlog     97  0.139      0.282 0.199   0.200
## 105 Disinfectant.QuatAmmonium FFNlog     69 -0.114     -0.327 0.262   0.358
## 106                Obs.Toilet FFNlog     56 -0.043     -0.150 0.162   0.277
## 107              Obs.GI.signs FFNlog     93  0.066      0.139 0.176   0.235
## 108                     CuExp FFNlog    157  0.354      0.445 0.335   0.318
## 109                     ZnExp FFNlog     11  0.032      0.566 0.197   0.652
## 110           FeedSource.Farm FFNlog     39  0.008      0.040 0.140   0.316
## 111                   WaterQC FFNlog     35 -0.066     -0.373 0.160   0.175
## 112              Source.Water FFNlog     74  0.086      0.229 0.124   0.087
## 113               Source.Soil FFNlog     46  0.073      0.311 0.149   0.145
## 114          Source.Fecal.Sow FFNlog     59  0.077      0.257 0.324   0.556
## 115       Source.Swab.Surface FFNlog     73  0.065      0.174 0.170   0.243
## 116            SampleStage.N1 FFNlog      8  0.011      0.261 0.062   0.184
## 117            SampleStage.N2 FFNlog      3 -0.007     -0.461 0.065   0.308
## 118            SampleStage.F1 FFNlog     12 -0.027     -0.443 0.115   0.185
## 119            SampleStage.F2 FFNlog     18 -0.035     -0.378 0.123   0.190
## 120                     Birds NALlog     47 -0.004     -0.017 0.140   0.288
## 121              Chicks.Ducks NALlog     63  0.087      0.271 0.220   0.319
## 122                 Ruminants NALlog     44 -0.076     -0.342 0.168   0.188
## 123              Visitors.Day NALlog     13  0.001      0.020 0.028   0.111
## 124       Disinfectant.Clorox NALlog     13  0.010      0.147 0.047   0.120
## 125 Disinfectant.QuatAmmonium NALlog     58  0.067      0.227 0.120   0.111
## 126                Obs.Toilet NALlog      7  0.005      0.148 0.041   0.175
## 127              Obs.GI.signs NALlog    109 -0.072     -0.130 0.107   0.114
## 128                     CuExp NALlog     43 -0.059     -0.272 0.178   0.297
## 129                     ZnExp NALlog     31 -0.063     -0.399 0.194   0.327
## 130           FeedSource.Farm NALlog    130 -0.149     -0.225 0.159   0.144
## 131                   WaterQC NALlog     16  0.008      0.097 0.032   0.062
## 132              Source.Water NALlog     63 -0.049     -0.155 0.080   0.060
## 133               Source.Soil NALlog    100  0.086      0.169 0.109   0.096
## 134          Source.Fecal.Sow NALlog     50 -0.067     -0.263 0.189   0.300
## 135       Source.Swab.Surface NALlog     20  0.002      0.022 0.052   0.166
## 136            SampleStage.N1 NALlog     12  0.000     -0.003 0.025   0.107
## 137            SampleStage.N2 NALlog      5  0.000      0.001 0.025   0.178
## 138            SampleStage.F1 NALlog     27 -0.021     -0.155 0.059   0.069
## 139            SampleStage.F2 NALlog      7  0.004      0.100 0.020   0.046
## 140                     Birds TELlog    119  0.014      0.023 0.420   0.541
## 141              Chicks.Ducks TELlog     95 -0.053     -0.110 0.554   0.796
## 142                 Ruminants TELlog     24  0.007      0.060 0.217   0.630
## 143              Visitors.Day TELlog      3 -0.002     -0.157 0.051   0.473
## 144       Disinfectant.Clorox TELlog    128 -0.107     -0.165 0.431   0.526
## 145 Disinfectant.QuatAmmonium TELlog     88  0.209      0.468 0.543   0.735
## 146                Obs.Toilet TELlog     33  0.093      0.553 0.294   0.516
## 147              Obs.GI.signs TELlog     19  0.003      0.029 0.134   0.442
## 148                     CuExp TELlog    167  0.555      0.655 0.531   0.516
## 149                     ZnExp TELlog    155  0.440      0.559 0.814   0.881
## 150           FeedSource.Farm TELlog    161  1.420      1.738 0.811   0.500
## 151                   WaterQC TELlog      2  0.002      0.182 0.019   0.054
## 152              Source.Water TELlog    127 -0.269     -0.418 0.278   0.240
## 153               Source.Soil TELlog    133  0.214      0.318 0.310   0.332
## 154          Source.Fecal.Sow TELlog    110 -0.181     -0.325 0.706   0.921
## 155       Source.Swab.Surface TELlog     72  0.034      0.093 0.235   0.383
## 156            SampleStage.N1 TELlog     21 -0.029     -0.274 0.156   0.410
## 157            SampleStage.N2 TELlog     38 -0.018     -0.092 0.143   0.317
## 158            SampleStage.F1 TELlog     25 -0.017     -0.131 0.174   0.481
## 159            SampleStage.F2 TELlog     44 -0.060     -0.270 0.193   0.335
## 160                     Birds CLIlog    149 -0.321     -0.425 0.471   0.500
## 161              Chicks.Ducks CLIlog     50  0.132      0.521 0.415   0.693
## 162                 Ruminants CLIlog      6 -0.004     -0.128 0.175   1.086
## 163              Visitors.Day CLIlog     22 -0.060     -0.537 0.194   0.291
## 164       Disinfectant.Clorox CLIlog    126  0.309      0.483 0.422   0.440
## 165 Disinfectant.QuatAmmonium CLIlog    109 -0.174     -0.314 0.534   0.688
## 166                Obs.Toilet CLIlog    145 -0.377     -0.512 0.569   0.609
## 167              Obs.GI.signs CLIlog      5 -0.002     -0.065 0.090   0.624
## 168                     CuExp CLIlog    159 -0.557     -0.690 0.575   0.564
## 169                     ZnExp CLIlog     65  0.398      1.207 0.728   0.795
## 170           FeedSource.Farm CLIlog    188  1.115      1.168 0.735   0.709
## 171              Source.Water CLIlog     54  0.033      0.121 0.273   0.515
## 172               Source.Soil CLIlog    150  0.424      0.557 0.414   0.389
## 173          Source.Fecal.Sow CLIlog    105 -0.061     -0.115 0.464   0.632
## 174       Source.Swab.Surface CLIlog     64 -0.126     -0.389 0.296   0.410
## 175            SampleStage.N1 CLIlog     29  0.036      0.247 0.175   0.400
## 176            SampleStage.N2 CLIlog      5  0.004      0.158 0.075   0.496
## 177            SampleStage.F1 CLIlog      6 -0.014     -0.445 0.099   0.387
## 178            SampleStage.F2 CLIlog      3  0.001      0.055 0.052   0.515
##       B_50 CI_lower CI_upper n_na
## 1   -1.215   -3.791    0.147    0
## 2    0.000   -1.813    5.328    0
## 3    0.000   -0.025    0.000    0
## 4    0.000    0.000    0.115    0
## 5   -0.614   -2.437    0.609    0
## 6    0.000   -3.988    0.000    0
## 7    1.390    0.000    3.892    0
## 8    0.000    0.000    1.675    0
## 9    1.070   -0.459    2.873    0
## 10   2.543    0.000    5.236    0
## 11   4.348    0.000    6.645    0
## 12   0.000   -2.331    1.353    0
## 13   0.891   -0.098    3.100    0
## 14   0.000   -2.338    2.673    0
## 15   0.000   -2.606    1.499    0
## 16   0.000    0.000    2.905    0
## 17   0.000   -0.058    1.977    0
## 18   0.000    0.000    2.265    0
## 19   0.000    0.000    2.172    0
## 20   0.000   -0.307    0.519    0
## 21  -0.064   -1.166    0.000    0
## 22   0.000   -0.021    0.271    0
## 23   0.000   -0.449    0.000    0
## 24   0.000   -0.342    0.598    0
## 25   0.000   -0.563    0.290    0
## 26   0.000   -0.003    0.000    0
## 27   0.000    0.000    0.335    0
## 28   0.248    0.000    0.737    0
## 29   0.000   -0.120    0.471    0
## 30   0.000    0.000    0.551    0
## 31   0.000   -0.384    0.000    0
## 32   0.000    0.000    0.350    0
## 33   0.000   -0.298    0.243    0
## 34   0.000   -1.235    1.470    0
## 35   0.000   -0.362    0.188    0
## 36   0.000   -0.124    0.162    0
## 37   0.000   -0.346    0.060    0
## 38  -0.132   -0.467    0.000    0
## 39   0.000   -0.454    0.000    0
## 40  -0.472   -1.808    0.439    0
## 41   0.530   -0.008    3.614    0
## 42   0.000    0.000    0.000    0
## 43   0.000    0.000    0.000    0
## 44  -0.682   -1.806    0.114    0
## 45  -0.804   -2.645    0.000    0
## 46   0.000    0.000    2.331    0
## 47   0.000    0.000    1.325    0
## 48   0.596   -0.323    2.189    0
## 49   1.756    0.000    3.989    0
## 50   2.763    0.000    4.447    0
## 51   0.000    0.000    0.000    0
## 52   0.000   -1.420    1.004    0
## 53   0.363   -0.107    1.940    0
## 54   0.000   -1.093    1.402    0
## 55   0.000   -1.565    0.849    0
## 56   0.000   -0.002    0.000    0
## 57   0.000   -0.363    0.000    0
## 58   0.000    0.000    0.580    0
## 59   0.000    0.000    0.000    0
## 60   0.000   -0.447    0.000    0
## 61  -0.213   -0.807    0.000    0
## 62   0.000    0.000    0.298    0
## 63  -0.293   -0.555    0.000    0
## 64   0.000    0.000    0.255    0
## 65   0.000    0.000    0.520    0
## 66   0.000   -0.096    0.337    0
## 67   0.000    0.000    0.349    0
## 68   0.000   -0.567    0.007    0
## 69   0.000    0.000    0.000    0
## 70   0.000   -0.407    0.053    0
## 71   0.000    0.000    0.000    0
## 72   0.362    0.144    0.506    0
## 73   0.000    0.000    0.520    0
## 74   0.000   -0.483    0.463    0
## 75   0.000   -0.196    0.150    0
## 76   0.000   -0.227    0.000    0
## 77   0.000    0.000    0.329    0
## 78   0.000    0.000    0.000    0
## 79   0.000    0.000    0.000    0
## 80  -2.476   -4.602    0.000    0
## 81   0.492   -2.553    4.882    0
## 82   0.000   -0.033    1.584    0
## 83   0.000   -0.038    0.000    0
## 84  -0.916   -3.024    0.125    0
## 85   0.000   -1.252    1.427    0
## 86   0.000   -2.357    0.000    0
## 87   0.000   -2.399    1.041    0
## 88   1.619    0.000    4.443    0
## 89   0.616   -0.150    2.231    0
## 90   0.000    0.000    1.991    0
## 91   0.000   -0.829    1.397    0
## 92  -1.181   -2.438    0.000    0
## 93   0.000   -1.953    1.551    0
## 94   0.000   -4.441    3.838    0
## 95   0.000   -1.295    1.126    0
## 96   0.000   -1.157    0.900    0
## 97  -1.435   -2.543    0.000    0
## 98   0.000    0.000    1.521    0
## 99   0.000   -1.518    0.353    0
## 100  0.000   -0.333    0.000    0
## 101  0.000    0.000    1.120    0
## 102  0.000   -0.427    0.216    0
## 103 -0.044   -0.358    0.553    0
## 104  0.000    0.000    0.523    0
## 105  0.000   -0.800    0.255    0
## 106  0.000   -0.416    0.214    0
## 107  0.000   -0.372    0.447    0
## 108  0.313    0.000    1.142    0
## 109  0.000    0.000    0.528    0
## 110  0.000   -0.254    0.323    0
## 111  0.000   -0.570    0.000    0
## 112  0.000    0.000    0.365    0
## 113  0.000    0.000    0.483    0
## 114  0.000   -0.625    0.837    0
## 115  0.000   -0.302    0.402    0
## 116  0.000    0.000    0.195    0
## 117  0.000    0.000    0.000    0
## 118  0.000   -0.430    0.000    0
## 119  0.000   -0.490    0.000    0
## 120  0.000   -0.269    0.384    0
## 121  0.000   -0.253    0.702    0
## 122  0.000   -0.547    0.000    0
## 123  0.000   -0.024    0.058    0
## 124  0.000    0.000    0.189    0
## 125  0.000    0.000    0.363    0
## 126  0.000    0.000    0.028    0
## 127 -0.010   -0.298    0.067    0
## 128  0.000   -0.682    0.000    0
## 129  0.000   -0.716    0.000    0
## 130 -0.122   -0.581    0.000    0
## 131  0.000    0.000    0.122    0
## 132  0.000   -0.257    0.000    0
## 133  0.000    0.000    0.331    0
## 134  0.000   -0.583    0.199    0
## 135  0.000   -0.118    0.159    0
## 136  0.000   -0.030    0.030    0
## 137  0.000    0.000    0.000    0
## 138  0.000   -0.230    0.000    0
## 139  0.000    0.000    0.065    0
## 140  0.000   -0.682    1.031    0
## 141  0.000   -1.294    1.411    0
## 142  0.000   -0.467    0.504    0
## 143  0.000    0.000    0.000    0
## 144  0.000   -0.949    0.821    0
## 145  0.000   -0.662    1.541    0
## 146  0.000    0.000    0.961    0
## 147  0.000   -0.335    0.242    0
## 148  0.558   -0.494    1.537    0
## 149  0.313   -0.951    1.901    0
## 150  1.617    0.000    2.530    0
## 151  0.000    0.000    0.000    0
## 152 -0.258   -0.831    0.000    0
## 153  0.138   -0.231    0.976    0
## 154  0.000   -2.007    0.811    0
## 155  0.000   -0.499    0.548    0
## 156  0.000   -0.529    0.005    0
## 157  0.000   -0.432    0.206    0
## 158  0.000   -0.682    0.287    0
## 159  0.000   -0.667    0.103    0
## 160 -0.248   -1.328    0.549    0
## 161  0.000   -0.350    1.228    0
## 162  0.000    0.000    0.000    0
## 163  0.000   -0.852    0.000    0
## 164  0.157   -0.356    1.263    0
## 165  0.000   -1.252    0.889    0
## 166 -0.200   -1.706    0.573    0
## 167  0.000    0.000    0.000    0
## 168 -0.482   -1.589    0.355    0
## 169  0.000   -0.085    1.850    0
## 170  1.314   -0.541    2.176    0
## 171  0.000   -0.539    0.741    0
## 172  0.389   -0.114    1.362    0
## 173  0.000   -1.049    1.080    0
## 174  0.000   -0.936    0.199    0
## 175  0.000   -0.081    0.555    0
## 176  0.000    0.000    0.000    0
## 177  0.000   -0.012    0.000    0
## 178  0.000    0.000    0.000    0
## 
## 
## OMEGA EDGES:
##        Y1     Y2 n_non0 Omega_bar SE_Omega Omega_bar_non0 SE_Omega_non0
## 1  AZIlog CIPlog    189    -0.040    0.030         -0.041         0.029
## 2  AZIlog ERYlog    197     0.794    0.069          0.794         0.069
## 3  AZIlog GENlog    109    -0.037    0.050         -0.067         0.051
## 4  AZIlog TETlog    144     0.061    0.073          0.084         0.073
## 5  AZIlog FFNlog    197    -0.176    0.066         -0.176         0.066
## 6  AZIlog NALlog     78    -0.017    0.035         -0.044         0.044
## 7  AZIlog TELlog    197    -0.273    0.132         -0.273         0.132
## 8  AZIlog CLIlog    197     0.236    0.083          0.236         0.083
## 9  CIPlog ERYlog      5     0.000    0.004         -0.018         0.018
## 10 CIPlog GENlog    152     0.067    0.056          0.086         0.049
## 11 CIPlog TETlog    149     0.049    0.060          0.064         0.061
## 12 CIPlog FFNlog    193     0.130    0.061          0.133         0.058
## 13 CIPlog NALlog    197     0.772    0.088          0.772         0.088
## 14 CIPlog TELlog    102     0.028    0.051          0.055         0.060
## 15 CIPlog CLIlog     33     0.002    0.031          0.012         0.077
## 16 ERYlog GENlog     58    -0.018    0.036         -0.062         0.041
## 17 ERYlog TETlog    190     0.011    0.063          0.011         0.064
## 18 ERYlog FFNlog     74    -0.022    0.058         -0.058         0.083
## 19 ERYlog NALlog     40    -0.009    0.029         -0.046         0.050
## 20 ERYlog TELlog    197     0.489    0.090          0.489         0.090
## 21 ERYlog CLIlog    197     0.167    0.082          0.167         0.082
## 22 GENlog TETlog    129    -0.036    0.045         -0.054         0.046
## 23 GENlog FFNlog    197     0.427    0.077          0.427         0.077
## 24 GENlog NALlog    129     0.055    0.056          0.084         0.048
## 25 GENlog TELlog     93     0.036    0.055          0.076         0.059
## 26 GENlog CLIlog    190     0.140    0.074          0.145         0.070
## 27 TETlog FFNlog    168     0.089    0.074          0.105         0.069
## 28 TETlog NALlog    161     0.063    0.056          0.077         0.053
## 29 TETlog TELlog    197     0.472    0.067          0.472         0.067
## 30 TETlog CLIlog    197    -0.293    0.067         -0.293         0.067
## 31 FFNlog NALlog     62    -0.013    0.039         -0.041         0.061
## 32 FFNlog TELlog    149     0.098    0.098          0.129         0.093
## 33 FFNlog CLIlog    130     0.084    0.095          0.127         0.090
## 34 NALlog TELlog     40     0.005    0.044          0.026         0.096
## 35 NALlog CLIlog    168     0.073    0.070          0.086         0.068
## 36 TELlog CLIlog    197     0.334    0.088          0.334         0.088
##    Omega_50 CI_lower CI_upper n_na
## 1    -0.036   -0.104    0.000    0
## 2     0.788    0.658    0.904    0
## 3    -0.005   -0.165    0.000    0
## 4     0.045   -0.046    0.227    0
## 5    -0.178   -0.296   -0.035    0
## 6     0.000   -0.123    0.000    0
## 7    -0.267   -0.507   -0.060    0
## 8     0.243    0.068    0.389    0
## 9     0.000    0.000    0.000    0
## 10    0.070    0.000    0.176    0
## 11    0.031   -0.019    0.194    0
## 12    0.124    0.024    0.264    0
## 13    0.793    0.589    0.863    0
## 14    0.001    0.000    0.170    0
## 15    0.000   -0.022    0.070    0
## 16    0.000   -0.124    0.000    0
## 17    0.011   -0.133    0.127    0
## 18    0.000   -0.161    0.054    0
## 19    0.000   -0.102    0.000    0
## 20    0.479    0.336    0.644    0
## 21    0.164    0.012    0.336    0
## 22   -0.018   -0.140    0.007    0
## 23    0.425    0.283    0.596    0
## 24    0.043    0.000    0.168    0
## 25    0.000    0.000    0.193    0
## 26    0.138    0.000    0.279    0
## 27    0.087   -0.002    0.225    0
## 28    0.059    0.000    0.165    0
## 29    0.480    0.334    0.581    0
## 30   -0.300   -0.414   -0.143    0
## 31    0.000   -0.137    0.047    0
## 32    0.089   -0.053    0.274    0
## 33    0.050    0.000    0.304    0
## 34    0.000   -0.050    0.088    0
## 35    0.060    0.000    0.249    0
## 36    0.326    0.182    0.487    0
```

#### Genetic

```
X <- abf.gen_cent

model.cg = l1ML_Main(
  Y = Y, 
  X = X,
  initializer = 'Lasso', 
  screening = T,
  ss = T,
  alpha = 0.05,
  nboot = 20
)
```

```
## Step 0: screening is on, proceed with debiased Lasso in conjunction with BH correction, the cut-off for p-values is set at 0.05 .
## Step 1: Penalized LS initialization with Lasso .
## Step 2: Alternate update.
```

```
## Warning in l1ML_Main(Y = Y, X = X, initializer = "Lasso", screening = T, : l1ML_Main(): alternate update is broken manually at iteration =50
```

```
## Step 3: Refitting B Matrix.
## Step 4: Stability selection with a total number of 20 bootstrapped samples ...25%...50%...75%...Done!
## Step 5: Refitting with weighted glasso ...Done.
## Returns: B.est, Theta.est, BICvalue.
```

```
model.cg_formatted <- format.chain.graph(model.cg, x.names = colnames(X), y.names = colnames(Y))
print(display.chain.graph(model.cg_formatted))
```

```
## 
## Lambda: 
## Edges: 57 (49%)
## 
## Beta Matrix:
##                 AZIlog CIPlog ERYlog GENlog TETlog FFNlog NALlog TELlog CLIlog
## X23S_A2075G     9.212 -0.374  6.015  0.514  0.000  0.000  0.000  2.333  3.931
## X50S_L22_A103V  0.000  0.000  0.000  0.000 -1.182  0.000  0.000  0.000  0.000
## aad9            0.000  0.000 -0.844  0.287 -1.273  0.372  0.229  0.000 -1.107
## aadE_Cc         0.000  0.000  0.000  0.000  0.000  0.000  0.000  0.000  0.000
## acr3            0.000  0.167  0.000  0.000  0.000  0.000  0.212  0.283  0.324
## aph.3pr_IIIa    5.512  0.000  3.986  0.000  2.609  0.105  0.000  1.861  3.434
## blaOXA_193     -2.837  0.535 -1.403  0.377 -1.688  0.496  0.000 -0.139  0.000
## blaOXA_489     -3.339  0.000 -1.521  0.000  0.000  0.858 -0.431  0.000  0.000
## blaOXA_578      0.000  0.000  0.000  0.000  0.000  0.000  0.000  0.000  0.000
## gyrA_T86I       0.879  7.038  0.812  0.000  0.000  0.000  4.118 -0.403 -0.190
## lnu.C          -2.622  0.000 -1.645  0.554 -2.760  0.566  0.688  0.000  1.951
## rpsL_K43R       0.000  0.000  0.000  0.000  0.000  0.000  0.484  0.552  0.550
## tet.O           0.000  1.000  0.000  0.339  4.178  0.969  1.251  0.346 -0.341
## 
## 
## Rho: 
## Edges: 29 (81%)
## 
## Omega Matrix: 
##        AZIlog CIPlog ERYlog GENlog TETlog FFNlog NALlog TELlog CLIlog
## AZIlog  0.000 -0.143  0.814 -0.062 -0.061 -0.161  0.333 -0.105  0.174
## CIPlog -0.144  0.000  0.000  0.164  0.160  0.267  0.357  0.223  0.000
## ERYlog  0.814  0.000  0.000  0.000  0.000  0.008 -0.096  0.445  0.080
## GENlog -0.062  0.164  0.000  0.000  0.145  0.220  0.163  0.000  0.104
## TETlog -0.061  0.160  0.000  0.145  0.000  0.123  0.000  0.389 -0.106
## FFNlog -0.161  0.267  0.008  0.220  0.123  0.000  0.328  0.000  0.132
## NALlog  0.333  0.357 -0.096  0.163  0.000  0.328  0.000 -0.163 -0.345
## TELlog -0.105  0.223  0.444  0.000  0.389  0.000 -0.163  0.000  0.371
## CLIlog  0.174  0.000  0.080  0.104 -0.106  0.132 -0.345  0.371  0.000
## 
## 
## NULL
```

```
R2.tab <- ChainGraph_R2_v4(
  X, 
  Y, 
  Beta = model.cg_formatted$B.est, 
  Omega = model.cg_formatted$Omega.est
)

print(R2.tab)
```

```
##          Y
## AZIlog AZI
## CIPlog CIP
## ERYlog ERY
## GENlog GEN
## TETlog TET
## FFNlog FFN
## NALlog NAL
## TELlog TEL
## CLIlog CLI
##                                                                            X_set
## AZIlog       X23S_A2075G, aph.3pr_IIIa, blaOXA_193, blaOXA_489, gyrA_T86I, lnu.C
## CIPlog                           X23S_A2075G, acr3, blaOXA_193, gyrA_T86I, tet.O
## ERYlog X23S_A2075G, aad9, aph.3pr_IIIa, blaOXA_193, blaOXA_489, gyrA_T86I, lnu.C
## GENlog                               X23S_A2075G, aad9, blaOXA_193, lnu.C, tet.O
## TETlog              X50S_L22_A103V, aad9, aph.3pr_IIIa, blaOXA_193, lnu.C, tet.O
## FFNlog                  aad9, aph.3pr_IIIa, blaOXA_193, blaOXA_489, lnu.C, tet.O
## NALlog                aad9, acr3, blaOXA_489, gyrA_T86I, lnu.C, rpsL_K43R, tet.O
## TELlog  X23S_A2075G, acr3, aph.3pr_IIIa, blaOXA_193, gyrA_T86I, rpsL_K43R, tet.O
## CLIlog X23S_A2075G, aad9, acr3, aph.3pr_IIIa, gyrA_T86I, lnu.C, rpsL_K43R, tet.O
##                 R2_X                                  Y_set   R2_e_Yj     R2_Yj
## AZIlog  0.4177917496 CIP, ERY, GEN, TET, FFN, NAL, TEL, CLI 0.9548402 0.5559159
## CIPlog  0.5828199421           AZI, GEN, TET, FFN, NAL, TEL 0.6190083 0.2582379
## ERYlog  0.3434202992                AZI, FFN, NAL, TEL, CLI 0.9656957 0.6340562
## GENlog  0.0975629626           AZI, CIP, TET, FFN, NAL, CLI 0.4491224 0.4053047
## TETlog  0.8012924271           AZI, CIP, GEN, FFN, TEL, CLI 0.5061665 0.1005791
## FFNlog -0.0004356317      AZI, CIP, ERY, GEN, TET, NAL, CLI 0.6494595 0.6497425
## NALlog  0.1031411495      AZI, CIP, ERY, GEN, FFN, TEL, CLI 0.7024206 0.6299722
## TELlog  0.2766864556           AZI, CIP, ERY, TET, NAL, CLI 0.9072696 0.6562404
## CLIlog  0.3840458584      AZI, ERY, GEN, TET, FFN, NAL, TEL 0.8691037 0.5353280
##         R2_total
## AZIlog 0.9737076
## CIPlog 0.8410579
## ERYlog 0.9774765
## GENlog 0.5028676
## TETlog 0.9018715
## FFNlog 0.6493068
## NALlog 0.7331133
## TELlog 0.9329268
## CLIlog 0.9193739
```

##### Bootstrap

```
#doParallel::registerDoParallel(cl)

model.cg_bs <- cg.bootstrap(
  Y = Y, 
  X = X, 
  b = B,
  n_b = n_b_abf,
  lambda = model.cg$lambda, 
  rho = model.cg$rho,
  initializer = 'Lasso', 
  screening = T,
  alpha = 0.05,
  nboot = 20,
  cluster = cl,
  seed.val = seeds[4]
)
```

```
## Starting parallel bootstrap at 2025-01-10 22:01:56.165985
```

```
## Ending bootstrap at 2025-01-10 22:11:45.869291
```

```
#stopCluster(cl)

summary.cg_bootstrap_results(model.cg_bs)
```

```
## Warning in summary.cg_bootstrap_results(model.cg_bs): Target number of
## subsamples (b = 200) exceeds available valid subsamples (b = 191).
```

```
## SAMPLE SIZES
## Original:  399 
## Subsample size:  200 
## 
## BOOTSTRAPS
## Total fit subsamples:  200 
## Subsamples for summary stats: 191 
## Subsamples that returned NAs: 9 
## 
## PENALTIES
## Lambda:  0.08017758 
## Rho:  0.07420801 
## 
## CI alpha: 95 %
## 
## BETA EDGES:
##                  X      Y n_non0  B_bar B_bar_non0  SE_B SE_non0   B_50
## 1      X23S_A2075G AZIlog    191  9.214      9.214 0.581   0.581  9.159
## 2   X50S_L22_A103V AZIlog     25 -0.279     -2.134 0.819   1.092  0.000
## 3             aad9 AZIlog     92  0.516      1.071 1.010   1.236  0.000
## 4             acr3 AZIlog     76  0.577      1.450 0.790   0.547  0.000
## 5     aph.3pr_IIIa AZIlog    191  5.044      5.044 1.361   1.361  5.019
## 6       blaOXA_193 AZIlog    191 -3.117     -3.117 0.533   0.533 -3.075
## 7       blaOXA_489 AZIlog    178 -3.254     -3.492 1.314   1.009 -3.494
## 8       blaOXA_578 AZIlog     21 -0.190     -1.728 0.584   0.672  0.000
## 9        gyrA_T86I AZIlog    126  1.559      2.363 2.244   2.396  0.722
## 10           lnu.C AZIlog    175 -2.436     -2.658 1.195   0.982 -2.608
## 11       rpsL_K43R AZIlog     58 -0.195     -0.641 0.736   1.232  0.000
## 12           tet.O AZIlog     43  0.801      3.559 1.508   0.490  0.000
## 13     X23S_A2075G CIPlog    109 -0.238     -0.417 0.237   0.153 -0.220
## 14  X50S_L22_A103V CIPlog     10 -0.032     -0.607 0.194   0.638  0.000
## 15            aad9 CIPlog     55  0.029      0.099 0.204   0.373  0.000
## 16         aadE_Cc CIPlog     30 -0.054     -0.346 0.149   0.202  0.000
## 17            acr3 CIPlog     82  0.129      0.300 0.188   0.176  0.000
## 18    aph.3pr_IIIa CIPlog     69  0.137      0.378 0.208   0.167  0.000
## 19      blaOXA_193 CIPlog    181  0.506      0.534 0.182   0.142  0.521
## 20      blaOXA_489 CIPlog     50 -0.014     -0.053 0.250   0.489  0.000
## 21      blaOXA_578 CIPlog      1  0.003      0.500 0.036   0.000  0.000
## 22       gyrA_T86I CIPlog    191  6.998      6.998 0.315   0.315  6.996
## 23           lnu.C CIPlog     90  0.177      0.375 0.324   0.386  0.000
## 24       rpsL_K43R CIPlog     52  0.033      0.120 0.219   0.410  0.000
## 25           tet.O CIPlog    183  0.824      0.860 0.412   0.382  0.756
## 26     X23S_A2075G ERYlog    191  6.073      6.073 0.545   0.545  6.029
## 27  X50S_L22_A103V ERYlog     35 -0.351     -1.915 0.856   1.004  0.000
## 28            aad9 ERYlog    120 -0.385     -0.613 1.106   1.346  0.000
## 29         aadE_Cc ERYlog      1  0.001      0.147 0.011   0.000  0.000
## 30            acr3 ERYlog     70  0.423      1.153 0.621   0.455  0.000
## 31    aph.3pr_IIIa ERYlog    190  3.526      3.544 1.287   1.264  3.516
## 32      blaOXA_193 ERYlog    188 -1.587     -1.612 0.399   0.347 -1.562
## 33      blaOXA_489 ERYlog    115 -1.280     -2.125 1.184   0.722 -1.329
## 34      blaOXA_578 ERYlog      7 -0.051     -1.383 0.333   1.169  0.000
## 35       gyrA_T86I ERYlog    147  1.521      1.976 1.399   1.281  1.315
## 36           lnu.C ERYlog    134 -1.235     -1.761 1.130   0.944 -1.267
## 37       rpsL_K43R ERYlog     68  0.186      0.524 0.563   0.849  0.000
## 38           tet.O ERYlog     92  1.038      2.156 1.398   1.283  0.000
## 39     X23S_A2075G GENlog    173  0.490      0.541 0.222   0.163  0.528
## 40  X50S_L22_A103V GENlog     19 -0.018     -0.180 0.091   0.239  0.000
## 41            aad9 GENlog    122  0.321      0.503 0.318   0.258  0.302
## 42         aadE_Cc GENlog     19  0.029      0.292 0.097   0.139  0.000
## 43            acr3 GENlog     76  0.092      0.230 0.141   0.133  0.000
## 44    aph.3pr_IIIa GENlog     27  0.021      0.148 0.126   0.311  0.000
## 45      blaOXA_193 GENlog    181  0.325      0.343 0.137   0.116  0.332
## 46      blaOXA_489 GENlog     91  0.044      0.093 0.190   0.268  0.000
## 47      blaOXA_578 GENlog     11 -0.012     -0.211 0.070   0.220  0.000
## 48       gyrA_T86I GENlog     47 -0.094     -0.383 0.257   0.400  0.000
## 49           lnu.C GENlog    168  0.522      0.593 0.244   0.158  0.541
## 50       rpsL_K43R GENlog     44 -0.054     -0.235 0.171   0.294  0.000
## 51           tet.O GENlog     57  0.100      0.336 0.249   0.360  0.000
## 52     X23S_A2075G TETlog     31  0.074      0.458 0.285   0.578  0.000
## 53  X50S_L22_A103V TETlog     94 -0.773     -1.571 0.865   0.511  0.000
## 54            aad9 TETlog     93 -0.561     -1.153 0.796   0.787  0.000
## 55         aadE_Cc TETlog      1  0.002      0.458 0.033   0.000  0.000
## 56            acr3 TETlog     33  0.105      0.607 0.250   0.242  0.000
## 57    aph.3pr_IIIa TETlog    187  2.022      2.065 0.999   0.965  1.752
## 58      blaOXA_193 TETlog    191 -1.771     -1.771 0.226   0.226 -1.755
## 59      blaOXA_489 TETlog     65  0.233      0.684 0.738   1.141  0.000
## 60      blaOXA_578 TETlog      3 -0.012     -0.735 0.097   0.296  0.000
## 61       gyrA_T86I TETlog    116  0.822      1.353 1.513   1.748  0.000
## 62           lnu.C TETlog    173 -2.277     -2.514 0.952   0.634 -2.503
## 63       rpsL_K43R TETlog     70  0.551      1.503 0.842   0.708  0.000
## 64           tet.O TETlog    191  4.769      4.769 0.875   0.875  4.574
## 65     X23S_A2075G FFNlog     46  0.102      0.422 0.201   0.178  0.000
## 66  X50S_L22_A103V FFNlog     23  0.022      0.186 0.174   0.480  0.000
## 67            aad9 FFNlog    178  0.555      0.595 0.316   0.288  0.502
## 68         aadE_Cc FFNlog     16  0.001      0.017 0.111   0.395  0.000
## 69            acr3 FFNlog     80  0.241      0.576 0.307   0.178  0.000
## 70    aph.3pr_IIIa FFNlog     59 -0.020     -0.064 0.152   0.269  0.000
## 71      blaOXA_193 FFNlog    144  0.291      0.386 0.226   0.175  0.322
## 72      blaOXA_489 FFNlog    187  0.838      0.856 0.343   0.324  0.819
## 73      blaOXA_578 FFNlog      8 -0.002     -0.054 0.109   0.565  0.000
## 74       gyrA_T86I FFNlog     60  0.118      0.376 0.402   0.650  0.000
## 75           lnu.C FFNlog    140  0.476      0.649 0.388   0.304  0.481
## 76       rpsL_K43R FFNlog     38 -0.013     -0.064 0.244   0.549  0.000
## 77           tet.O FFNlog    127  0.594      0.893 0.602   0.527  0.486
## 78     X23S_A2075G NALlog     25  0.022      0.169 0.109   0.262  0.000
## 79  X50S_L22_A103V NALlog     35 -0.074     -0.402 0.188   0.249  0.000
## 80            aad9 NALlog    122  0.248      0.388 0.242   0.194  0.272
## 81         aadE_Cc NALlog     47  0.050      0.205 0.107   0.122  0.000
## 82            acr3 NALlog    186  0.296      0.304 0.168   0.163  0.291
## 83    aph.3pr_IIIa NALlog      5  0.003      0.121 0.056   0.364  0.000
## 84      blaOXA_489 NALlog    102 -0.210     -0.393 0.267   0.249 -0.051
## 85      blaOXA_578 NALlog      4 -0.001     -0.060 0.033   0.250  0.000
## 86       gyrA_T86I NALlog    191  3.966      3.966 0.416   0.416  3.951
## 87           lnu.C NALlog    172  0.655      0.727 0.355   0.295  0.644
## 88       rpsL_K43R NALlog    128  0.370      0.552 0.334   0.256  0.358
## 89           tet.O NALlog    170  0.824      0.926 0.582   0.534  0.802
## 90     X23S_A2075G TELlog    191  2.237      2.237 0.384   0.384  2.252
## 91  X50S_L22_A103V TELlog     90 -0.393     -0.833 0.641   0.712  0.000
## 92            aad9 TELlog    126  0.518      0.785 0.599   0.578  0.401
## 93         aadE_Cc TELlog      2  0.000     -0.032 0.017   0.229  0.000
## 94            acr3 TELlog    157  0.629      0.766 0.512   0.463  0.537
## 95    aph.3pr_IIIa TELlog    151  1.533      1.940 1.084   0.834  1.499
## 96      blaOXA_193 TELlog    142 -0.304     -0.409 0.277   0.246 -0.268
## 97      blaOXA_489 TELlog     79 -0.099     -0.240 0.797   1.231  0.000
## 98      blaOXA_578 TELlog      5  0.000      0.006 0.102   0.701  0.000
## 99       gyrA_T86I TELlog    100  0.034      0.066 0.500   0.692  0.000
## 100          lnu.C TELlog    116 -0.045     -0.074 0.895   1.150  0.000
## 101      rpsL_K43R TELlog    115  0.392      0.652 0.524   0.536  0.301
## 102          tet.O TELlog    114  0.898      1.504 1.149   1.140  0.494
## 103    X23S_A2075G CLIlog    191  3.862      3.862 0.338   0.338  3.877
## 104 X50S_L22_A103V CLIlog     12 -0.003     -0.045 0.292   1.211  0.000
## 105           aad9 CLIlog     93 -0.436     -0.895 0.636   0.648  0.000
## 106        aadE_Cc CLIlog      4 -0.005     -0.242 0.038   0.122  0.000
## 107           acr3 CLIlog    125  0.486      0.742 0.532   0.492  0.311
## 108   aph.3pr_IIIa CLIlog    191  2.915      2.915 0.753   0.753  3.016
## 109     blaOXA_193 CLIlog     61 -0.078     -0.244 0.192   0.275  0.000
## 110     blaOXA_489 CLIlog     83 -0.506     -1.163 0.642   0.426  0.000
## 111     blaOXA_578 CLIlog      2  0.008      0.796 0.082   0.159  0.000
## 112      gyrA_T86I CLIlog    102  0.128      0.240 0.406   0.532  0.000
## 113          lnu.C CLIlog    167  1.910      2.184 0.827   0.425  2.199
## 114      rpsL_K43R CLIlog    103  0.294      0.546 0.424   0.444  0.000
## 115          tet.O CLIlog     92  0.361      0.749 0.807   1.032  0.000
##     CI_lower CI_upper n_na
## 1      8.234   10.477    0
## 2     -2.927    0.000    0
## 3     -0.974    2.895    0
## 4      0.000    2.376    0
## 5      2.701    7.943    0
## 6     -4.368   -2.264    0
## 7     -5.315    0.000    0
## 8     -2.386    0.000    0
## 9     -0.739    8.068    0
## 10    -4.507    0.000    0
## 11    -1.738    1.521    0
## 12     0.000    4.105    0
## 13    -0.671    0.000    0
## 14    -0.442    0.000    0
## 15    -0.484    0.421    0
## 16    -0.537    0.000    0
## 17     0.000    0.593    0
## 18     0.000    0.613    0
## 19     0.000    0.763    0
## 20    -0.475    0.392    0
## 21     0.000    0.000    0
## 22     6.378    7.693    0
## 23    -0.373    0.755    0
## 24    -0.296    0.443    0
## 25     0.000    1.834    0
## 26     5.087    7.322    0
## 27    -3.022    0.000    0
## 28    -2.800    2.175    0
## 29     0.000    0.000    0
## 30     0.000    1.783    0
## 31     1.500    6.166    0
## 32    -2.453   -1.008    0
## 33    -3.356    0.000    0
## 34    -0.302    0.000    0
## 35     0.000    4.937    0
## 36    -3.645    0.000    0
## 37    -0.889    1.424    0
## 38     0.000    3.830    0
## 39     0.000    0.853    0
## 40    -0.257    0.000    0
## 41     0.000    0.861    0
## 42     0.000    0.337    0
## 43     0.000    0.431    0
## 44    -0.178    0.400    0
## 45     0.000    0.568    0
## 46    -0.350    0.428    0
## 47    -0.234    0.000    0
## 48    -0.733    0.000    0
## 49     0.000    0.927    0
## 50    -0.524    0.192    0
## 51    -0.114    0.904    0
## 52    -0.059    0.882    0
## 53    -2.401    0.000    0
## 54    -2.326    0.252    0
## 55     0.000    0.000    0
## 56     0.000    0.843    0
## 57     0.664    3.987    0
## 58    -2.240   -1.326    0
## 59    -1.293    1.786    0
## 60     0.000    0.000    0
## 61    -1.010    5.270    0
## 62    -3.758    0.000    0
## 63     0.000    2.702    0
## 64     3.388    6.517    0
## 65     0.000    0.655    0
## 66     0.000    0.463    0
## 67     0.000    1.281    0
## 68    -0.173    0.242    0
## 69     0.000    0.821    0
## 70    -0.510    0.225    0
## 71     0.000    0.646    0
## 72     0.125    1.497    0
## 73     0.000    0.007    0
## 74    -0.608    1.277    0
## 75     0.000    1.288    0
## 76    -0.729    0.564    0
## 77    -0.015    1.822    0
## 78    -0.137    0.314    0
## 79    -0.641    0.000    0
## 80     0.000    0.776    0
## 81     0.000    0.400    0
## 82     0.000    0.623    0
## 83     0.000    0.000    0
## 84    -0.764    0.009    0
## 85     0.000    0.000    0
## 86     3.243    4.875    0
## 87     0.000    1.322    0
## 88     0.000    1.144    0
## 89     0.000    1.943    0
## 90     1.524    2.907    0
## 91    -2.333    0.038    0
## 92    -0.326    1.775    0
## 93     0.000    0.000    0
## 94     0.000    1.775    0
## 95     0.000    3.526    0
## 96    -0.985    0.000    0
## 97    -1.578    1.490    0
## 98     0.000    0.000    0
## 99    -0.854    1.179    0
## 100   -2.132    1.611    0
## 101   -0.524    1.452    0
## 102    0.000    3.299    0
## 103    3.221    4.531    0
## 104   -0.809    0.915    0
## 105   -1.820    0.056    0
## 106    0.000    0.000    0
## 107    0.000    1.766    0
## 108    1.641    4.149    0
## 109   -0.725    0.129    0
## 110   -1.718    0.000    0
## 111    0.000    0.000    0
## 112   -0.479    0.905    0
## 113    0.000    2.847    0
## 114   -0.236    1.240    0
## 115   -0.598    2.002    0
## 
## 
## OMEGA EDGES:
##        Y1     Y2 n_non0 Omega_bar SE_Omega Omega_bar_non0 SE_Omega_non0
## 1  AZIlog CIPlog    173    -0.091    0.060         -0.100         0.055
## 2  AZIlog ERYlog    191     0.794    0.061          0.794         0.061
## 3  AZIlog GENlog    136    -0.054    0.056         -0.076         0.053
## 4  AZIlog TETlog    120    -0.017    0.052         -0.027         0.063
## 5  AZIlog FFNlog    191    -0.185    0.085         -0.185         0.085
## 6  AZIlog NALlog    171     0.131    0.154          0.146         0.155
## 7  AZIlog TELlog    191    -0.042    0.139         -0.042         0.139
## 8  AZIlog CLIlog    191     0.168    0.118          0.168         0.118
## 9  CIPlog ERYlog     13    -0.002    0.009         -0.028         0.025
## 10 CIPlog GENlog    191     0.186    0.063          0.186         0.063
## 11 CIPlog TETlog    190     0.177    0.066          0.178         0.065
## 12 CIPlog FFNlog    191     0.292    0.065          0.292         0.065
## 13 CIPlog NALlog    189     0.356    0.072          0.360         0.063
## 14 CIPlog TELlog    171     0.138    0.088          0.154         0.079
## 15 CIPlog CLIlog     44     0.022    0.049          0.094         0.063
## 16 ERYlog GENlog     74    -0.017    0.030         -0.044         0.033
## 17 ERYlog TETlog     48     0.009    0.024          0.035         0.038
## 18 ERYlog FFNlog     89     0.005    0.050          0.012         0.072
## 19 ERYlog NALlog    135    -0.067    0.082         -0.094         0.083
## 20 ERYlog TELlog    191     0.365    0.095          0.365         0.095
## 21 ERYlog CLIlog    191     0.131    0.092          0.131         0.092
## 22 GENlog TETlog    172     0.141    0.092          0.157         0.083
## 23 GENlog FFNlog    187     0.220    0.072          0.224         0.066
## 24 GENlog NALlog    119     0.078    0.080          0.125         0.066
## 25 GENlog TELlog     22    -0.004    0.027         -0.035         0.075
## 26 GENlog CLIlog    152     0.099    0.085          0.125         0.077
## 27 TETlog FFNlog    156     0.120    0.095          0.147         0.083
## 28 TETlog NALlog    121     0.081    0.100          0.128         0.100
## 29 TETlog TELlog    191     0.371    0.096          0.371         0.096
## 30 TETlog CLIlog    175    -0.187    0.109         -0.204         0.098
## 31 FFNlog NALlog    167     0.188    0.181          0.216         0.177
## 32 FFNlog TELlog     97     0.045    0.109          0.089         0.140
## 33 FFNlog CLIlog    143     0.115    0.109          0.153         0.100
## 34 NALlog TELlog    105    -0.103    0.138         -0.186         0.137
## 35 NALlog CLIlog    109    -0.109    0.129         -0.191         0.115
## 36 TELlog CLIlog    191     0.379    0.098          0.379         0.098
##    Omega_50 CI_lower CI_upper n_na
## 1    -0.089   -0.217    0.000    0
## 2     0.797    0.678    0.900    0
## 3    -0.037   -0.191    0.000    0
## 4     0.000   -0.140    0.074    0
## 5    -0.181   -0.352    0.003    0
## 6     0.098   -0.084    0.424    0
## 7    -0.031   -0.310    0.198    0
## 8     0.178   -0.081    0.371    0
## 9     0.000   -0.040    0.000    0
## 10    0.177    0.064    0.313    0
## 11    0.176    0.043    0.298    0
## 12    0.298    0.166    0.409    0
## 13    0.361    0.188    0.472    0
## 14    0.139    0.000    0.317    0
## 15    0.000    0.000    0.180    0
## 16    0.000   -0.104    0.000    0
## 17    0.000   -0.003    0.075    0
## 18    0.000   -0.140    0.108    0
## 19   -0.042   -0.243    0.026    0
## 20    0.357    0.200    0.550    0
## 21    0.123   -0.038    0.312    0
## 22    0.142    0.000    0.325    0
## 23    0.228    0.056    0.340    0
## 24    0.069    0.000    0.237    0
## 25    0.000   -0.061    0.005    0
## 26    0.088    0.000    0.280    0
## 27    0.119    0.000    0.297    0
## 28    0.042   -0.041    0.291    0
## 29    0.381    0.166    0.532    0
## 30   -0.211   -0.372    0.000    0
## 31    0.186   -0.065    0.530    0
## 32    0.000   -0.162    0.286    0
## 33    0.111    0.000    0.334    0
## 34    0.000   -0.346    0.113    0
## 35   -0.057   -0.369    0.013    0
## 36    0.388    0.159    0.548    0
```

#### Environmental + Genetic

```
X <- abf.env_gen_cent

model.cg = l1ML_Main(
  Y = Y, 
  X = X, 
  initializer = 'Lasso', 
  screening = T,
  ss = T,
  alpha = 0.05,
  nboot = 20
)
```

```
## Step 0: screening is on, proceed with debiased Lasso in conjunction with BH correction, the cut-off for p-values is set at 0.05 .
## Step 1: Penalized LS initialization with Lasso .
## Step 2: Alternate update.
## Step 3: Refitting B Matrix.
## Step 4: Stability selection with a total number of 20 bootstrapped samples ...25%...50%...75%...Done!
## Step 5: Refitting with weighted glasso ...Done.
## Returns: B.est, Theta.est, BICvalue.
```

```
model.cg_formatted <- format.chain.graph(model.cg, x.names = colnames(X), y.names = colnames(Y))
print(display.chain.graph(model.cg_formatted))
```

```
## 
## Lambda: 
## Edges: 96 (32%)
## 
## Beta Matrix:
##                            AZIlog CIPlog ERYlog GENlog TETlog FFNlog NALlog
## X23S_A2075G                7.873 -0.300  5.675  0.530  0.000  0.000  0.389
## X50S_L22_A103V             0.000  0.139  0.000  0.000 -1.552  0.000  0.312
## aad9                       6.711  0.000  3.356  0.000 -1.207  0.000  0.000
## aadE_Cc                    0.000  0.000  0.390  0.000  0.000  0.029  0.000
## acr3                       0.135 -0.035  0.000  0.000  0.000  0.072  0.484
## aph.3pr_IIIa               0.000  0.000  0.000  0.000  2.221  0.000  0.000
## blaOXA_193                 0.000  0.439  0.000  0.000 -2.011  0.000 -0.419
## blaOXA_489                -3.621 -0.072  0.000  0.000  0.000  0.734 -1.170
## blaOXA_578                 0.000  0.239  0.000  0.000  0.000  0.791  0.000
## gyrA_T86I                 -3.645  6.843  0.000  0.000  0.742  0.000  3.273
## lnu.C                      0.000  0.000  0.834  0.000 -3.021  0.000  0.342
## rpsL_K43R                 -2.954  0.000  0.000  0.000  0.000  0.000  0.000
## tet.O                      2.879  0.678  3.439 -0.126  4.936  0.000  0.000
## Birds                      1.020  0.288  0.899  0.000  0.000  0.000  0.000
## Chicks.Ducks              -1.700  0.000  0.000 -0.249  0.000  0.394  0.000
## Ruminants                  0.000  0.000  0.000  0.000  0.000  0.000 -0.321
## Visitors.Day               0.000  0.000  0.000 -0.263  0.000 -0.327  0.000
## Disinfectant.Clorox        0.000  0.000  0.000  0.000  0.000  0.277 -0.251
## Disinfectant.QuatAmmonium -1.614  0.000 -1.342  0.267 -0.487  0.237  0.000
## Obs.Toilet                 1.851  0.000  0.903  0.000  0.000  0.000  0.000
## Obs.GI.signs               0.000  0.000  0.000  0.000 -0.180  0.000  0.000
## CuExp                      1.828  0.000  0.900  0.000  0.000  0.000  0.376
## ZnExp                      4.637  0.000  2.890  0.000  0.000  0.000  0.169
## FeedSource.Farm            4.394  0.000  2.699  0.000  0.000  0.000  0.000
## WaterQC                    0.000  0.000  0.000  0.000  0.000  0.000  0.000
## Source.Water               0.000  0.273  0.000  0.194  0.278  0.000  0.000
## Source.Soil                0.000  0.000  0.000  0.000  0.000  0.000  0.000
## Source.Fecal.Sow           0.000  0.000  0.000  0.000  0.000  0.000  0.000
## Source.Swab.Surface        0.000  0.000  0.000  0.000  0.297  0.000  0.000
## SampleStage.N1             0.000  0.110  0.000  0.000  0.000  0.000  0.000
## SampleStage.N2             0.000  0.000  0.000  0.000  0.000  0.000  0.000
## SampleStage.F1             0.000 -0.199  0.000  0.000  0.000  0.000  0.000
## SampleStage.F2             0.000 -0.231  0.000  0.000  0.000  0.000  0.000
##                           TELlog CLIlog
## X23S_A2075G                2.249  3.925
## X50S_L22_A103V             0.000  0.000
## aad9                       1.592  0.791
## aadE_Cc                    0.573  0.000
## acr3                       0.000  0.000
## aph.3pr_IIIa               0.000  0.612
## blaOXA_193                 0.000  0.000
## blaOXA_489                 0.000  0.000
## blaOXA_578                 0.000  0.000
## gyrA_T86I                 -0.433  0.000
## lnu.C                      0.306  1.898
## rpsL_K43R                  0.000  0.000
## tet.O                      2.141  2.035
## Birds                      0.132 -0.243
## Chicks.Ducks               0.000  0.000
## Ruminants                  0.000  0.000
## Visitors.Day               0.000  0.000
## Disinfectant.Clorox        0.000  0.000
## Disinfectant.QuatAmmonium  0.000 -0.211
## Obs.Toilet                 0.000 -0.093
## Obs.GI.signs               0.000  0.000
## CuExp                      0.455  0.000
## ZnExp                      1.487  1.221
## FeedSource.Farm            1.533  1.380
## WaterQC                    0.000  0.000
## Source.Water               0.000  0.000
## Source.Soil                0.000  0.328
## Source.Fecal.Sow           0.000  0.000
## Source.Swab.Surface        0.000  0.000
## SampleStage.N1             0.000  0.000
## SampleStage.N2             0.000  0.000
## SampleStage.F1             0.202  0.000
## SampleStage.F2             0.000  0.000
## 
## 
## Rho: 
## Edges: 30 (83%)
## 
## Omega Matrix: 
##        AZIlog CIPlog ERYlog GENlog TETlog FFNlog NALlog TELlog CLIlog
## AZIlog  0.000 -0.036  0.688 -0.039  0.027 -0.252  0.169  0.114  0.118
## CIPlog -0.036  0.000  0.000  0.149  0.170  0.257  0.380  0.091  0.000
## ERYlog  0.688  0.000  0.000 -0.090  0.113  0.000 -0.251  0.255  0.280
## GENlog -0.039  0.149 -0.090  0.000  0.192  0.143  0.149  0.000  0.277
## TETlog  0.027  0.170  0.113  0.192  0.000  0.177  0.021  0.260 -0.224
## FFNlog -0.252  0.257  0.000  0.144  0.177  0.000  0.198  0.340  0.000
## NALlog  0.170  0.380 -0.251  0.149  0.021  0.198  0.000  0.000 -0.131
## TELlog  0.114  0.091  0.255  0.000  0.260  0.340  0.000  0.000  0.325
## CLIlog  0.118  0.000  0.280  0.277 -0.224  0.000 -0.131  0.325  0.000
## 
## 
## NULL
```

```
R2.tab <- ChainGraph_R2_v4(
  X, 
  Y, 
  Beta = model.cg_formatted$B.est, 
  Omega = model.cg_formatted$Omega.est
)

print(R2.tab)
```

```
##          Y
## AZIlog AZI
## CIPlog CIP
## ERYlog ERY
## GENlog GEN
## TETlog TET
## FFNlog FFN
## NALlog NAL
## TELlog TEL
## CLIlog CLI
##                                                                                                                                                               X_set
## AZIlog  X23S_A2075G, aad9, acr3, blaOXA_489, gyrA_T86I, rpsL_K43R, tet.O, Birds, Chicks.Ducks, Disinfectant.QuatAmmonium, Obs.Toilet, CuExp, ZnExp, FeedSource.Farm
## CIPlog X23S_A2075G, X50S_L22_A103V, acr3, blaOXA_193, blaOXA_489, blaOXA_578, gyrA_T86I, tet.O, Birds, Source.Water, SampleStage.N1, SampleStage.F1, SampleStage.F2
## ERYlog                                        X23S_A2075G, aad9, aadE_Cc, lnu.C, tet.O, Birds, Disinfectant.QuatAmmonium, Obs.Toilet, CuExp, ZnExp, FeedSource.Farm
## GENlog                                                                      X23S_A2075G, tet.O, Chicks.Ducks, Visitors.Day, Disinfectant.QuatAmmonium, Source.Water
## TETlog          X50S_L22_A103V, aad9, aph.3pr_IIIa, blaOXA_193, gyrA_T86I, lnu.C, tet.O, Disinfectant.QuatAmmonium, Obs.GI.signs, Source.Water, Source.Swab.Surface
## FFNlog                                            aadE_Cc, acr3, blaOXA_489, blaOXA_578, Chicks.Ducks, Visitors.Day, Disinfectant.Clorox, Disinfectant.QuatAmmonium
## NALlog                                    X23S_A2075G, X50S_L22_A103V, acr3, blaOXA_193, blaOXA_489, gyrA_T86I, lnu.C, Ruminants, Disinfectant.Clorox, CuExp, ZnExp
## TELlog                                                    X23S_A2075G, aad9, aadE_Cc, gyrA_T86I, lnu.C, tet.O, Birds, CuExp, ZnExp, FeedSource.Farm, SampleStage.F1
## CLIlog                             X23S_A2075G, aad9, aph.3pr_IIIa, lnu.C, tet.O, Birds, Disinfectant.QuatAmmonium, Obs.Toilet, ZnExp, FeedSource.Farm, Source.Soil
##             R2_X                                  Y_set   R2_e_Yj      R2_Yj
## AZIlog 0.7702842 CIP, ERY, GEN, TET, FFN, NAL, TEL, CLI 0.8791291 0.20194984
## CIPlog 0.6070089           AZI, GEN, TET, FFN, NAL, TEL 0.6009110 0.23615271
## ERYlog 0.7147804           AZI, GEN, TET, NAL, TEL, CLI 0.9076210 0.25887125
## GENlog 0.2343046      AZI, CIP, ERY, TET, FFN, NAL, CLI 0.4397696 0.33672957
## TETlog 0.8166289 AZI, CIP, ERY, GEN, FFN, NAL, TEL, CLI 0.5380109 0.09865565
## FFNlog 0.3019295           AZI, CIP, GEN, TET, NAL, TEL 0.6247582 0.43612531
## NALlog 0.1182494      AZI, CIP, ERY, GEN, TET, FFN, CLI 0.6040430 0.53261529
## TELlog 0.6909283           AZI, CIP, ERY, TET, FFN, CLI 0.7982350 0.24671185
## CLIlog 0.6673260           AZI, ERY, GEN, TET, NAL, TEL 0.7860985 0.26151459
##         R2_total
## AZIlog 0.9722341
## CIPlog 0.8431616
## ERYlog 0.9736517
## GENlog 0.5710342
## TETlog 0.9152845
## FFNlog 0.7380548
## NALlog 0.6508647
## TELlog 0.9376401
## CLIlog 0.9288405
```

##### Bootstrap

```
#cl <- parallel::makeCluster(n_cl)
#doParallel::registerDoParallel(cl)

model.cg_bs <- cg.bootstrap(
  Y = Y, 
  X = X, 
  b = B,
  n_b = n_b_abf,
  lambda = model.cg$lambda, 
  rho = model.cg$rho,
  initializer = 'Lasso', 
  screening = T,
  alpha = 0.05,
  nboot = 20,
  cluster = cl,
  seed.val = seeds[5]
)
```

```
## Starting parallel bootstrap at 2025-01-10 22:12:02.328045
```

```
## Ending bootstrap at 2025-01-10 22:21:19.493616
```

```
stopCluster(cl)

summary.cg_bootstrap_results(model.cg_bs)
```

```
## Warning in summary.cg_bootstrap_results(model.cg_bs): Target number of
## subsamples (b = 200) exceeds available valid subsamples (b = 183).
```

```
## SAMPLE SIZES
## Original:  399 
## Subsample size:  200 
## 
## BOOTSTRAPS
## Total fit subsamples:  200 
## Subsamples for summary stats: 183 
## Subsamples that returned NAs: 17 
## 
## PENALTIES
## Lambda:  0.09361184 
## Rho:  0.07420801 
## 
## CI alpha: 95 %
## 
## BETA EDGES:
##                             X      Y n_non0  B_bar B_bar_non0  SE_B SE_non0
## 1                 X23S_A2075G AZIlog    183  8.209      8.209 1.266   1.266
## 2              X50S_L22_A103V AZIlog     67  1.111      3.035 1.604   1.079
## 3                        aad9 AZIlog    182  5.430      5.460 2.087   2.053
## 4                     aadE_Cc AZIlog     88  0.779      1.621 1.040   0.940
## 5                        acr3 AZIlog    116 -0.389     -0.614 0.561   0.599
## 6                aph.3pr_IIIa AZIlog     83  1.656      3.652 2.144   1.683
## 7                  blaOXA_193 AZIlog     16 -0.062     -0.707 0.282   0.692
## 8                  blaOXA_489 AZIlog    154 -3.457     -4.108 2.106   1.608
## 9                  blaOXA_578 AZIlog      1  0.008      1.385 0.102   0.000
## 10                  gyrA_T86I AZIlog    164 -2.616     -2.919 2.464   2.426
## 11                      lnu.C AZIlog    156 -0.136     -0.159 1.683   1.823
## 12                  rpsL_K43R AZIlog    143 -2.295     -2.937 1.686   1.321
## 13                      tet.O AZIlog    130  2.206      3.106 1.718   1.161
## 14                      Birds AZIlog    157  0.970      1.131 0.876   0.844
## 15               Chicks.Ducks AZIlog    162 -2.393     -2.703 1.652   1.497
## 16  Disinfectant.QuatAmmonium AZIlog    179 -1.605     -1.641 1.104   1.090
## 17                 Obs.Toilet AZIlog    165  1.844      2.045 0.875   0.660
## 18               Obs.GI.signs AZIlog     73 -0.716     -1.794 1.032   0.855
## 19                      CuExp AZIlog    181  2.670      2.700 1.307   1.284
## 20                      ZnExp AZIlog    143  3.673      4.700 2.049   0.721
## 21            FeedSource.Farm AZIlog    181  4.556      4.606 1.255   1.165
## 22                    WaterQC AZIlog      3 -0.047     -2.851 0.365   0.328
## 23               Source.Water AZIlog     74  0.249      0.615 0.443   0.511
## 24                Source.Soil AZIlog     62  0.189      0.558 0.369   0.445
## 25           Source.Fecal.Sow AZIlog     56 -0.181     -0.593 0.774   1.317
## 26        Source.Swab.Surface AZIlog      1  0.004      0.653 0.048   0.000
## 27             SampleStage.F1 AZIlog     11 -0.037     -0.618 0.191   0.521
## 28             SampleStage.F2 AZIlog      6 -0.010     -0.308 0.100   0.507
## 29                X23S_A2075G CIPlog     96 -0.179     -0.341 0.234   0.222
## 30             X50S_L22_A103V CIPlog     44 -0.050     -0.208 0.254   0.489
## 31                       aad9 CIPlog     33 -0.014     -0.075 0.294   0.697
## 32                    aadE_Cc CIPlog     56 -0.090     -0.294 0.194   0.253
## 33                       acr3 CIPlog    145  0.261      0.329 0.247   0.233
## 34               aph.3pr_IIIa CIPlog     76  0.186      0.448 0.484   0.671
## 35                 blaOXA_193 CIPlog     65  0.151      0.426 0.224   0.156
## 36                 blaOXA_489 CIPlog     55 -0.045     -0.148 0.248   0.438
## 37                 blaOXA_578 CIPlog     24  0.026      0.201 0.153   0.384
## 38                  gyrA_T86I CIPlog    183  7.115      7.115 0.379   0.379
## 39                      lnu.C CIPlog     55  0.059      0.196 0.328   0.578
## 40                  rpsL_K43R CIPlog     36  0.054      0.274 0.144   0.212
## 41                      tet.O CIPlog    166  0.544      0.599 0.272   0.219
## 42                      Birds CIPlog     94  0.210      0.410 0.234   0.158
## 43               Chicks.Ducks CIPlog    103 -0.345     -0.612 0.402   0.351
## 44                  Ruminants CIPlog     41  0.008      0.034 0.111   0.236
## 45               Visitors.Day CIPlog     18 -0.042     -0.427 0.132   0.117
## 46        Disinfectant.Clorox CIPlog     66  0.088      0.244 0.210   0.292
## 47  Disinfectant.QuatAmmonium CIPlog     88 -0.146     -0.304 0.262   0.309
## 48                 Obs.Toilet CIPlog     31 -0.050     -0.296 0.126   0.143
## 49               Obs.GI.signs CIPlog     68  0.125      0.337 0.197   0.183
## 50                      CuExp CIPlog     25 -0.023     -0.166 0.145   0.366
## 51                      ZnExp CIPlog     24 -0.023     -0.176 0.142   0.364
## 52            FeedSource.Farm CIPlog      8 -0.001     -0.022 0.073   0.371
## 53                    WaterQC CIPlog     17 -0.021     -0.221 0.069   0.083
## 54               Source.Water CIPlog    148  0.238      0.294 0.148   0.102
## 55                Source.Soil CIPlog     34  0.039      0.211 0.124   0.218
## 56           Source.Fecal.Sow CIPlog     85  0.230      0.496 0.413   0.486
## 57        Source.Swab.Surface CIPlog     40  0.028      0.130 0.117   0.224
## 58             SampleStage.N1 CIPlog     68  0.032      0.087 0.096   0.142
## 59             SampleStage.N2 CIPlog     33 -0.025     -0.141 0.095   0.184
## 60             SampleStage.F1 CIPlog     56 -0.073     -0.238 0.131   0.131
## 61             SampleStage.F2 CIPlog     79 -0.108     -0.251 0.149   0.126
## 62                X23S_A2075G ERYlog    183  5.587      5.587 0.945   0.945
## 63             X50S_L22_A103V ERYlog     63  0.528      1.535 0.790   0.512
## 64                       aad9 ERYlog    176  2.883      2.998 1.501   1.413
## 65                    aadE_Cc ERYlog    105  0.564      0.983 0.753   0.759
## 66                       acr3 ERYlog     84 -0.146     -0.317 0.324   0.419
## 67               aph.3pr_IIIa ERYlog     66  0.927      2.570 1.501   1.420
## 68                 blaOXA_193 ERYlog     46  0.057      0.228 0.298   0.566
## 69                 blaOXA_489 ERYlog     67 -0.976     -2.666 1.425   1.012
## 70                 blaOXA_578 ERYlog      2  0.006      0.583 0.153   1.893
## 71                  gyrA_T86I ERYlog    115 -0.263     -0.418 1.310   1.635
## 72                      lnu.C ERYlog    145  0.340      0.429 0.867   0.955
## 73                  rpsL_K43R ERYlog     66 -0.528     -1.464 0.850   0.794
## 74                      tet.O ERYlog    165  2.546      2.824 1.315   1.063
## 75                      Birds ERYlog    176  0.975      1.014 0.631   0.612
## 76               Chicks.Ducks ERYlog     61 -0.582     -1.746 0.976   0.908
## 77                  Ruminants ERYlog      5 -0.025     -0.933 0.157   0.252
## 78               Visitors.Day ERYlog      2  0.005      0.439 0.078   0.850
## 79        Disinfectant.Clorox ERYlog      2 -0.002     -0.205 0.103   1.352
## 80  Disinfectant.QuatAmmonium ERYlog    183 -1.316     -1.316 0.551   0.551
## 81                 Obs.Toilet ERYlog    131  0.823      1.150 0.620   0.400
## 82               Obs.GI.signs ERYlog      9 -0.061     -1.239 0.314   0.774
## 83                      CuExp ERYlog    176  1.084      1.127 0.791   0.776
## 84                      ZnExp ERYlog    174  2.565      2.697 1.295   1.185
## 85            FeedSource.Farm ERYlog    167  2.633      2.885 1.062   0.710
## 86                    WaterQC ERYlog      1 -0.009     -1.699 0.126   0.000
## 87               Source.Water ERYlog     83  0.202      0.446 0.308   0.318
## 88                Source.Soil ERYlog     38  0.012      0.057 0.205   0.452
## 89           Source.Fecal.Sow ERYlog     28  0.017      0.111 0.365   0.943
## 90        Source.Swab.Surface ERYlog      1 -0.001     -0.100 0.007   0.000
## 91             SampleStage.F1 ERYlog     40  0.014      0.063 0.151   0.321
## 92             SampleStage.F2 ERYlog     47  0.022      0.086 0.146   0.279
## 93                X23S_A2075G GENlog    163  0.500      0.561 0.310   0.271
## 94             X50S_L22_A103V GENlog     31  0.022      0.131 0.183   0.434
## 95                       aad9 GENlog     69  0.160      0.425 0.314   0.387
## 96                    aadE_Cc GENlog     16 -0.020     -0.224 0.084   0.191
## 97                       acr3 GENlog    116  0.132      0.209 0.184   0.194
## 98               aph.3pr_IIIa GENlog     55 -0.057     -0.191 0.409   0.733
## 99                 blaOXA_193 GENlog     44  0.041      0.172 0.113   0.176
## 100                blaOXA_489 GENlog     67  0.106      0.290 0.251   0.345
## 101                blaOXA_578 GENlog     19  0.009      0.089 0.096   0.294
## 102                 gyrA_T86I GENlog     37  0.036      0.177 0.282   0.613
## 103                     lnu.C GENlog     83  0.225      0.497 0.291   0.227
## 104                 rpsL_K43R GENlog     22 -0.010     -0.084 0.139   0.401
## 105                     tet.O GENlog     71 -0.096     -0.247 0.184   0.223
## 106                     Birds GENlog     31 -0.041     -0.241 0.135   0.246
## 107              Chicks.Ducks GENlog    117 -0.382     -0.597 0.360   0.271
## 108                 Ruminants GENlog     79  0.130      0.302 0.184   0.163
## 109              Visitors.Day GENlog     97 -0.185     -0.349 0.225   0.194
## 110       Disinfectant.Clorox GENlog     27  0.024      0.160 0.173   0.433
## 111 Disinfectant.QuatAmmonium GENlog     96  0.173      0.330 0.199   0.153
## 112                Obs.Toilet GENlog      8  0.008      0.192 0.051   0.168
## 113              Obs.GI.signs GENlog     13 -0.007     -0.101 0.070   0.252
## 114                     CuExp GENlog     35 -0.055     -0.288 0.180   0.322
## 115                     ZnExp GENlog     36 -0.041     -0.207 0.241   0.516
## 116           FeedSource.Farm GENlog      5 -0.004     -0.155 0.063   0.386
## 117                   WaterQC GENlog      6  0.007      0.218 0.040   0.044
## 118              Source.Water GENlog    155  0.217      0.256 0.136   0.109
## 119               Source.Soil GENlog     54  0.067      0.227 0.147   0.193
## 120          Source.Fecal.Sow GENlog     22 -0.029     -0.242 0.181   0.479
## 121       Source.Swab.Surface GENlog     39 -0.044     -0.208 0.125   0.199
## 122            SampleStage.N1 GENlog     24 -0.024     -0.180 0.071   0.101
## 123            SampleStage.N2 GENlog     44  0.034      0.140 0.084   0.122
## 124            SampleStage.F1 GENlog     12  0.004      0.054 0.050   0.195
## 125            SampleStage.F2 GENlog      8  0.001      0.015 0.038   0.191
## 126               X23S_A2075G TETlog     61 -0.172     -0.516 0.526   0.812
## 127            X50S_L22_A103V TETlog    135 -1.259     -1.707 1.025   0.810
## 128                      aad9 TETlog    117 -1.036     -1.621 1.159   1.073
## 129                   aadE_Cc TETlog     36 -0.034     -0.175 0.385   0.864
## 130                      acr3 TETlog     50  0.094      0.342 0.288   0.471
## 131              aph.3pr_IIIa TETlog    177  2.193      2.267 1.106   1.046
## 132                blaOXA_193 TETlog    182 -2.019     -2.030 0.418   0.391
## 133                blaOXA_489 TETlog     82  0.060      0.135 0.961   1.437
## 134                blaOXA_578 TETlog     45 -0.250     -1.016 0.481   0.402
## 135                 gyrA_T86I TETlog    132  1.060      1.469 1.485   1.567
## 136                     lnu.C TETlog    176 -2.828     -2.940 1.019   0.864
## 137                 rpsL_K43R TETlog    105  0.522      0.909 0.877   0.995
## 138                     tet.O TETlog    183  4.862      4.862 0.905   0.905
## 139                     Birds TETlog     63 -0.212     -0.617 0.327   0.246
## 140              Chicks.Ducks TETlog     60  0.156      0.475 0.416   0.615
## 141                 Ruminants TETlog     57 -0.018     -0.058 0.260   0.466
## 142              Visitors.Day TETlog     38 -0.073     -0.351 0.167   0.193
## 143       Disinfectant.Clorox TETlog     34  0.071      0.383 0.259   0.497
## 144 Disinfectant.QuatAmmonium TETlog    138 -0.473     -0.627 0.373   0.296
## 145                Obs.Toilet TETlog     17 -0.003     -0.029 0.085   0.287
## 146              Obs.GI.signs TETlog     82 -0.218     -0.486 0.319   0.312
## 147                     CuExp TETlog    104  0.262      0.461 0.468   0.542
## 148                     ZnExp TETlog     35  0.035      0.185 0.297   0.665
## 149           FeedSource.Farm TETlog     19  0.021      0.203 0.140   0.399
## 150                   WaterQC TETlog     11 -0.014     -0.228 0.076   0.224
## 151              Source.Water TETlog    120  0.276      0.420 0.409   0.440
## 152               Source.Soil TETlog     55  0.033      0.109 0.279   0.504
## 153          Source.Fecal.Sow TETlog     57 -0.055     -0.177 0.344   0.602
## 154       Source.Swab.Surface TETlog    115  0.365      0.580 0.383   0.329
## 155            SampleStage.N1 TETlog     11  0.010      0.172 0.067   0.223
## 156            SampleStage.N2 TETlog      7 -0.006     -0.158 0.077   0.387
## 157            SampleStage.F1 TETlog     26 -0.057     -0.403 0.155   0.171
## 158            SampleStage.F2 TETlog     11 -0.006     -0.101 0.060   0.236
## 159               X23S_A2075G FFNlog     47  0.041      0.160 0.185   0.340
## 160            X50S_L22_A103V FFNlog     33  0.000      0.000 0.267   0.638
## 161                      aad9 FFNlog     89  0.203      0.418 0.398   0.487
## 162                   aadE_Cc FFNlog     98 -0.133     -0.248 0.216   0.242
## 163                      acr3 FFNlog    152  0.287      0.345 0.295   0.291
## 164              aph.3pr_IIIa FFNlog     77 -0.045     -0.106 0.615   0.948
## 165                blaOXA_193 FFNlog     29 -0.012     -0.079 0.143   0.357
## 166                blaOXA_489 FFNlog    171  0.797      0.852 0.501   0.470
## 167                blaOXA_578 FFNlog    138  0.371      0.493 0.308   0.256
## 168                 gyrA_T86I FFNlog     60  0.095      0.291 0.417   0.692
## 169                     lnu.C FFNlog     70  0.167      0.436 0.317   0.382
## 170                 rpsL_K43R FFNlog     52 -0.044     -0.154 0.191   0.335
## 171                     tet.O FFNlog     41  0.043      0.191 0.224   0.447
## 172                     Birds FFNlog     37 -0.039     -0.192 0.154   0.300
## 173              Chicks.Ducks FFNlog    112  0.354      0.578 0.393   0.350
## 174                 Ruminants FFNlog     51  0.003      0.011 0.190   0.363
## 175              Visitors.Day FFNlog     74 -0.174     -0.430 0.247   0.202
## 176       Disinfectant.Clorox FFNlog     83  0.121      0.267 0.230   0.280
## 177 Disinfectant.QuatAmmonium FFNlog     68  0.112      0.302 0.196   0.214
## 178                Obs.Toilet FFNlog     10 -0.017     -0.304 0.077   0.154
## 179              Obs.GI.signs FFNlog     43  0.072      0.305 0.156   0.183
## 180                     CuExp FFNlog     55  0.101      0.336 0.247   0.353
## 181                     ZnExp FFNlog     41 -0.065     -0.292 0.268   0.510
## 182           FeedSource.Farm FFNlog      5 -0.008     -0.291 0.095   0.558
## 183                   WaterQC FFNlog      1 -0.002     -0.292 0.022   0.000
## 184              Source.Water FFNlog     41  0.021      0.093 0.102   0.202
## 185               Source.Soil FFNlog     42  0.052      0.228 0.118   0.146
## 186          Source.Fecal.Sow FFNlog     59  0.145      0.451 0.295   0.365
## 187       Source.Swab.Surface FFNlog     59  0.050      0.155 0.133   0.198
## 188            SampleStage.N1 FFNlog     36  0.059      0.302 0.156   0.226
## 189            SampleStage.N2 FFNlog     27  0.018      0.120 0.109   0.265
## 190            SampleStage.F1 FFNlog     23 -0.024     -0.189 0.110   0.258
## 191            SampleStage.F2 FFNlog     35 -0.036     -0.190 0.109   0.184
## 192               X23S_A2075G NALlog     50  0.072      0.263 0.318   0.570
## 193            X50S_L22_A103V NALlog     60  0.030      0.092 0.390   0.681
## 194                      aad9 NALlog     58  0.174      0.550 0.446   0.652
## 195                   aadE_Cc NALlog     66  0.045      0.124 0.236   0.383
## 196                      acr3 NALlog    166  0.351      0.387 0.351   0.350
## 197              aph.3pr_IIIa NALlog     59  0.149      0.462 0.554   0.903
## 198                blaOXA_193 NALlog    112 -0.195     -0.319 0.244   0.240
## 199                blaOXA_489 NALlog    145 -0.654     -0.825 0.558   0.501
## 200                blaOXA_578 NALlog     40  0.092      0.420 0.266   0.434
## 201                 gyrA_T86I NALlog    183  3.362      3.362 0.476   0.476
## 202                     lnu.C NALlog     92  0.360      0.717 0.522   0.535
## 203                 rpsL_K43R NALlog     93  0.199      0.392 0.300   0.318
## 204                     tet.O NALlog     78  0.080      0.188 0.271   0.392
## 205                     Birds NALlog     22  0.012      0.103 0.131   0.372
## 206              Chicks.Ducks NALlog     59  0.033      0.101 0.308   0.539
## 207                 Ruminants NALlog     68 -0.132     -0.354 0.279   0.362
## 208              Visitors.Day NALlog      1  0.003      0.506 0.037   0.000
## 209       Disinfectant.Clorox NALlog     58 -0.070     -0.219 0.185   0.275
## 210 Disinfectant.QuatAmmonium NALlog     27  0.010      0.065 0.117   0.303
## 211                Obs.Toilet NALlog     12  0.018      0.269 0.093   0.262
## 212              Obs.GI.signs NALlog     11 -0.011     -0.178 0.059   0.172
## 213                     CuExp NALlog     57  0.162      0.520 0.382   0.534
## 214                     ZnExp NALlog     98  0.161      0.300 0.562   0.742
## 215           FeedSource.Farm NALlog     12  0.029      0.445 0.134   0.308
## 216                   WaterQC NALlog      3  0.001      0.085 0.036   0.327
## 217              Source.Water NALlog     78  0.007      0.016 0.146   0.224
## 218               Source.Soil NALlog     72  0.063      0.160 0.173   0.248
## 219          Source.Fecal.Sow NALlog     36  0.007      0.033 0.225   0.512
## 220       Source.Swab.Surface NALlog     50  0.043      0.158 0.124   0.197
## 221            SampleStage.N1 NALlog     12  0.002      0.030 0.112   0.455
## 222            SampleStage.N2 NALlog     18  0.024      0.241 0.112   0.282
## 223            SampleStage.F1 NALlog     18 -0.024     -0.248 0.103   0.235
## 224            SampleStage.F2 NALlog     19 -0.002     -0.021 0.072   0.227
## 225               X23S_A2075G TELlog    183  2.225      2.225 0.512   0.512
## 226            X50S_L22_A103V TELlog     24 -0.080     -0.612 0.277   0.518
## 227                      aad9 TELlog    180  1.460      1.484 0.632   0.609
## 228                   aadE_Cc TELlog     85  0.207      0.445 0.383   0.459
## 229                      acr3 TELlog      6  0.020      0.614 0.135   0.474
## 230              aph.3pr_IIIa TELlog     58  0.277      0.875 0.799   1.227
## 231                blaOXA_193 TELlog     71  0.160      0.412 0.296   0.350
## 232                blaOXA_489 TELlog     60  0.067      0.204 0.655   1.138
## 233                blaOXA_578 TELlog     23 -0.063     -0.503 0.267   0.599
## 234                 gyrA_T86I TELlog    106 -0.275     -0.475 0.546   0.649
## 235                     lnu.C TELlog     95  0.226      0.436 0.510   0.641
## 236                 rpsL_K43R TELlog     73  0.254      0.637 0.439   0.490
## 237                     tet.O TELlog    180  2.102      2.137 0.813   0.772
## 238                     Birds TELlog    133  0.285      0.392 0.307   0.296
## 239              Chicks.Ducks TELlog     54 -0.087     -0.296 0.317   0.531
## 240                 Ruminants TELlog     30 -0.003     -0.019 0.177   0.443
## 241              Visitors.Day TELlog      1  0.002      0.333 0.025   0.000
## 242       Disinfectant.Clorox TELlog      3  0.003      0.212 0.051   0.412
## 243 Disinfectant.QuatAmmonium TELlog    100 -0.143     -0.262 0.426   0.550
## 244                Obs.Toilet TELlog     57  0.107      0.343 0.205   0.232
## 245              Obs.GI.signs TELlog      9 -0.029     -0.590 0.151   0.386
## 246                     CuExp TELlog    160  0.435      0.498 0.480   0.482
## 247                     ZnExp TELlog    163  1.018      1.142 0.818   0.779
## 248           FeedSource.Farm TELlog    157  1.371      1.598 0.716   0.483
## 249                   WaterQC TELlog      1  0.001      0.202 0.015   0.000
## 250              Source.Water TELlog     28 -0.035     -0.227 0.108   0.183
## 251               Source.Soil TELlog     53  0.052      0.178 0.158   0.254
## 252          Source.Fecal.Sow TELlog     58 -0.159     -0.501 0.360   0.489
## 253       Source.Swab.Surface TELlog     45  0.087      0.354 0.181   0.196
## 254            SampleStage.F1 TELlog     76  0.013      0.030 0.148   0.229
## 255            SampleStage.F2 TELlog     57  0.005      0.016 0.144   0.259
## 256               X23S_A2075G CLIlog    183  3.578      3.578 0.608   0.608
## 257            X50S_L22_A103V CLIlog     14  0.074      0.967 0.318   0.696
## 258                      aad9 CLIlog    130  0.255      0.360 0.806   0.937
## 259                   aadE_Cc CLIlog     78  0.192      0.452 0.445   0.591
## 260                      acr3 CLIlog      2  0.005      0.488 0.070   0.649
## 261              aph.3pr_IIIa CLIlog    150  1.506      1.837 1.110   0.945
## 262                blaOXA_193 CLIlog     79  0.271      0.628 0.362   0.279
## 263                blaOXA_489 CLIlog     42 -0.370     -1.613 0.777   0.793
## 264                blaOXA_578 CLIlog     54  0.092      0.313 0.326   0.542
## 265                 gyrA_T86I CLIlog     63 -0.053     -0.155 0.535   0.907
## 266                     lnu.C CLIlog    183  1.835      1.835 0.558   0.558
## 267                 rpsL_K43R CLIlog     15 -0.024     -0.288 0.199   0.659
## 268                     tet.O CLIlog    146  1.348      1.690 0.902   0.662
## 269                     Birds CLIlog     67 -0.016     -0.044 0.288   0.476
## 270              Chicks.Ducks CLIlog     31  0.002      0.011 0.261   0.643
## 271                 Ruminants CLIlog     15  0.031      0.382 0.166   0.461
## 272       Disinfectant.Clorox CLIlog     10  0.042      0.763 0.176   0.126
## 273 Disinfectant.QuatAmmonium CLIlog    118 -0.257     -0.398 0.410   0.452
## 274                Obs.Toilet CLIlog    102 -0.035     -0.063 0.220   0.292
## 275              Obs.GI.signs CLIlog      8  0.012      0.277 0.101   0.424
## 276                     CuExp CLIlog    117  0.000      0.001 0.413   0.517
## 277                     ZnExp CLIlog    156  0.839      0.984 0.893   0.891
## 278           FeedSource.Farm CLIlog    177  1.286      1.330 0.597   0.557
## 279              Source.Water CLIlog     83  0.171      0.377 0.225   0.183
## 280               Source.Soil CLIlog     90  0.222      0.451 0.273   0.219
## 281          Source.Fecal.Sow CLIlog     43  0.135      0.576 0.341   0.494
## 282       Source.Swab.Surface CLIlog      1  0.004      0.787 0.058   0.000
## 283            SampleStage.N1 CLIlog      1 -0.001     -0.106 0.008   0.000
## 284            SampleStage.F1 CLIlog     13  0.004      0.050 0.084   0.322
## 285            SampleStage.F2 CLIlog      7 -0.006     -0.165 0.052   0.223
##       B_50 CI_lower CI_upper n_na
## 1    8.011    6.152   10.684    0
## 2    0.000    0.000    4.678    0
## 3    5.841    0.959    8.301    0
## 4    0.000    0.000    3.165    0
## 5   -0.196   -1.567    0.605    0
## 6    0.000    0.000    6.349    0
## 7    0.000   -1.020    0.000    0
## 8   -3.659   -7.118    0.000    0
## 9    0.000    0.000    0.000    0
## 10  -2.842   -6.836    3.276    0
## 11   0.000   -3.943    3.015    0
## 12  -2.233   -5.455    0.000    0
## 13   2.445    0.000    5.098    0
## 14   0.850   -0.214    3.086    0
## 15  -2.102   -5.618    0.000    0
## 16  -1.311   -4.425   -0.181    0
## 17   1.802    0.000    3.487    0
## 18   0.000   -3.050    0.000    0
## 19   2.523    0.166    5.570    0
## 20   4.483    0.000    5.955    0
## 21   4.540    1.749    6.745    0
## 22   0.000    0.000    0.000    0
## 23   0.000    0.000    1.373    0
## 24   0.000   -0.058    1.059    0
## 25   0.000   -1.967    1.787    0
## 26   0.000    0.000    0.000    0
## 27   0.000   -0.697    0.000    0
## 28   0.000    0.000    0.000    0
## 29  -0.115   -0.753    0.000    0
## 30   0.000   -0.869    0.379    0
## 31   0.000   -0.689    0.674    0
## 32   0.000   -0.546    0.036    0
## 33   0.244    0.000    0.878    0
## 34   0.000   -0.009    1.898    0
## 35   0.000    0.000    0.690    0
## 36   0.000   -0.663    0.437    0
## 37   0.000   -0.198    0.504    0
## 38   7.097    6.351    7.896    0
## 39   0.000   -0.578    0.867    0
## 40   0.000    0.000    0.451    0
## 41   0.584    0.000    0.987    0
## 42   0.164    0.000    0.702    0
## 43  -0.270   -1.313    0.000    0
## 44   0.000   -0.274    0.264    0
## 45   0.000   -0.472    0.000    0
## 46   0.000   -0.235    0.568    0
## 47   0.000   -0.725    0.272    0
## 48   0.000   -0.472    0.000    0
## 49   0.000    0.000    0.587    0
## 50   0.000   -0.514    0.159    0
## 51   0.000   -0.326    0.000    0
## 52   0.000    0.000    0.064    0
## 53   0.000   -0.251    0.000    0
## 54   0.249    0.000    0.481    0
## 55   0.000   -0.089    0.384    0
## 56   0.000   -0.549    1.218    0
## 57   0.000   -0.173    0.306    0
## 58   0.000   -0.195    0.262    0
## 59   0.000   -0.299    0.112    0
## 60   0.000   -0.460    0.000    0
## 61   0.000   -0.462    0.000    0
## 62   5.551    3.864    7.521    0
## 63   0.000    0.000    2.139    0
## 64   3.127    0.000    5.101    0
## 65   0.270   -0.061    2.456    0
## 66   0.000   -0.910    0.386    0
## 67   0.000    0.000    4.955    0
## 68   0.000   -0.358    0.872    0
## 69   0.000   -4.316    0.000    0
## 70   0.000    0.000    0.000    0
## 71   0.000   -2.970    3.378    0
## 72   0.392   -1.663    1.828    0
## 73   0.000   -2.619    0.000    0
## 74   2.835    0.000    4.180    0
## 75   0.941    0.000    2.443    0
## 76   0.000   -2.752    0.000    0
## 77   0.000   -0.247    0.000    0
## 78   0.000    0.000    0.000    0
## 79   0.000    0.000    0.000    0
## 80  -1.212   -2.526   -0.501    0
## 81   0.939    0.000    1.832    0
## 82   0.000   -1.051    0.000    0
## 83   1.087   -0.510    2.723    0
## 84   3.033    0.000    4.140    0
## 85   2.804    0.000    4.281    0
## 86   0.000    0.000    0.000    0
## 87   0.000    0.000    1.008    0
## 88   0.000   -0.294    0.392    0
## 89   0.000   -0.602    0.891    0
## 90   0.000    0.000    0.000    0
## 91   0.000   -0.311    0.383    0
## 92   0.000   -0.269    0.408    0
## 93   0.552    0.000    0.954    0
## 94   0.000   -0.186    0.480    0
## 95   0.000   -0.371    0.823    0
## 96   0.000   -0.254    0.000    0
## 97   0.117   -0.120    0.550    0
## 98   0.000   -1.054    0.421    0
## 99   0.000    0.000    0.387    0
## 100  0.000   -0.139    0.877    0
## 101  0.000   -0.093    0.165    0
## 102  0.000   -0.511    0.827    0
## 103  0.000    0.000    0.858    0
## 104  0.000   -0.422    0.260    0
## 105  0.000   -0.543    0.000    0
## 106  0.000   -0.457    0.000    0
## 107 -0.393   -1.054    0.000    0
## 108  0.000    0.000    0.531    0
## 109 -0.113   -0.785    0.000    0
## 110  0.000   -0.239    0.547    0
## 111  0.152    0.000    0.532    0
## 112  0.000    0.000    0.166    0
## 113  0.000   -0.179    0.031    0
## 114  0.000   -0.503    0.130    0
## 115  0.000   -0.245    0.000    0
## 116  0.000    0.000    0.000    0
## 117  0.000    0.000    0.170    0
## 118  0.227    0.000    0.503    0
## 119  0.000   -0.162    0.445    0
## 120  0.000   -0.622    0.313    0
## 121  0.000   -0.350    0.000    0
## 122  0.000   -0.226    0.000    0
## 123  0.000    0.000    0.265    0
## 124  0.000   -0.082    0.143    0
## 125  0.000    0.000    0.035    0
## 126  0.000   -1.508    0.630    0
## 127 -1.295   -3.293    0.000    0
## 128 -1.031   -3.278    0.628    0
## 129  0.000   -1.208    0.783    0
## 130  0.000   -0.523    0.841    0
## 131  2.164    0.000    4.331    0
## 132 -2.001   -2.819   -1.336    0
## 133  0.000   -1.805    2.013    0
## 134  0.000   -1.498    0.000    0
## 135  0.658   -0.974    4.634    0
## 136 -2.899   -4.572    0.000    0
## 137  0.000   -0.625    2.582    0
## 138  4.867    3.068    6.543    0
## 139  0.000   -0.930    0.000    0
## 140  0.000   -0.500    1.256    0
## 141  0.000   -0.616    0.644    0
## 142  0.000   -0.568    0.000    0
## 143  0.000   -0.142    0.870    0
## 144 -0.469   -1.136    0.000    0
## 145  0.000   -0.217    0.169    0
## 146  0.000   -0.983    0.000    0
## 147  0.000   -0.559    1.283    0
## 148  0.000   -0.316    0.761    0
## 149  0.000    0.000    0.407    0
## 150  0.000   -0.256    0.000    0
## 151  0.270   -0.602    1.107    0
## 152  0.000   -0.622    0.659    0
## 153  0.000   -0.907    0.667    0
## 154  0.338    0.000    1.122    0
## 155  0.000    0.000    0.261    0
## 156  0.000    0.000    0.000    0
## 157  0.000   -0.549    0.000    0
## 158  0.000   -0.139    0.000    0
## 159  0.000   -0.285    0.489    0
## 160  0.000   -0.739    0.572    0
## 161  0.000   -0.572    1.000    0
## 162  0.000   -0.732    0.085    0
## 163  0.255    0.000    1.039    0
## 164  0.000   -0.991    1.790    0
## 165  0.000   -0.467    0.244    0
## 166  0.798    0.000    1.964    0
## 167  0.357    0.000    1.111    0
## 168  0.000   -0.523    1.258    0
## 169  0.000   -0.260    0.916    0
## 170  0.000   -0.470    0.389    0
## 171  0.000   -0.332    0.558    0
## 172  0.000   -0.459    0.324    0
## 173  0.265   -0.011    1.206    0
## 174  0.000   -0.491    0.450    0
## 175  0.000   -0.737    0.000    0
## 176  0.000   -0.165    0.705    0
## 177  0.000   -0.018    0.581    0
## 178  0.000   -0.284    0.000    0
## 179  0.000    0.000    0.500    0
## 180  0.000    0.000    0.836    0
## 181  0.000   -0.718    0.119    0
## 182  0.000    0.000    0.000    0
## 183  0.000    0.000    0.000    0
## 184  0.000   -0.227    0.293    0
## 185  0.000    0.000    0.364    0
## 186  0.000   -0.284    0.890    0
## 187  0.000   -0.193    0.341    0
## 188  0.000    0.000    0.508    0
## 189  0.000   -0.139    0.263    0
## 190  0.000   -0.378    0.000    0
## 191  0.000   -0.376    0.000    0
## 192  0.000   -0.383    0.931    0
## 193  0.000   -0.870    0.936    0
## 194  0.000    0.000    1.323    0
## 195  0.000   -0.472    0.648    0
## 196  0.270   -0.109    1.236    0
## 197  0.000   -0.125    1.911    0
## 198 -0.187   -0.678    0.153    0
## 199 -0.596   -1.857    0.000    0
## 200  0.000    0.000    0.865    0
## 201  3.335    2.565    4.261    0
## 202  0.000    0.000    1.716    0
## 203  0.000    0.000    0.922    0
## 204  0.000   -0.277    0.697    0
## 205  0.000   -0.186    0.303    0
## 206  0.000   -0.676    0.789    0
## 207  0.000   -1.006    0.177    0
## 208  0.000    0.000    0.000    0
## 209  0.000   -0.545    0.159    0
## 210  0.000   -0.283    0.303    0
## 211  0.000    0.000    0.230    0
## 212  0.000   -0.200    0.000    0
## 213  0.000   -0.156    1.184    0
## 214  0.000   -0.799    1.705    0
## 215  0.000    0.000    0.535    0
## 216  0.000    0.000    0.000    0
## 217  0.000   -0.212    0.345    0
## 218  0.000   -0.275    0.507    0
## 219  0.000   -0.369    0.577    0
## 220  0.000   -0.134    0.398    0
## 221  0.000   -0.163    0.000    0
## 222  0.000    0.000    0.278    0
## 223  0.000   -0.328    0.000    0
## 224  0.000   -0.123    0.131    0
## 225  2.312    1.094    3.021    0
## 226  0.000   -1.046    0.000    0
## 227  1.550    0.075    2.424    0
## 228  0.000   -0.003    1.400    0
## 229  0.000    0.000    0.170    0
## 230  0.000   -0.958    2.355    0
## 231  0.000   -0.079    0.822    0
## 232  0.000   -1.550    1.472    0
## 233  0.000   -0.766    0.000    0
## 234  0.000   -1.379    0.839    0
## 235  0.000   -0.792    1.365    0
## 236  0.000   -0.245    1.331    0
## 237  2.374    0.490    3.274    0
## 238  0.239   -0.169    0.979    0
## 239  0.000   -0.961    0.397    0
## 240  0.000   -0.477    0.449    0
## 241  0.000    0.000    0.000    0
## 242  0.000    0.000    0.000    0
## 243  0.000   -1.215    0.657    0
## 244  0.000    0.000    0.662    0
## 245  0.000   -0.697    0.000    0
## 246  0.495   -0.527    1.365    0
## 247  1.384   -0.806    1.928    0
## 248  1.576    0.000    2.341    0
## 249  0.000    0.000    0.000    0
## 250  0.000   -0.351    0.000    0
## 251  0.000   -0.180    0.535    0
## 252  0.000   -1.194    0.000    0
## 253  0.000    0.000    0.675    0
## 254  0.000   -0.352    0.344    0
## 255  0.000   -0.379    0.326    0
## 256  3.555    2.324    4.660    0
## 257  0.000    0.000    1.298    0
## 258  0.000   -1.216    2.045    0
## 259  0.000   -0.484    1.353    0
## 260  0.000    0.000    0.000    0
## 261  1.475    0.000    3.773    0
## 262  0.000    0.000    1.116    0
## 263  0.000   -2.515    0.000    0
## 264  0.000   -0.502    1.010    0
## 265  0.000   -1.483    1.086    0
## 266  1.853    0.607    2.850    0
## 267  0.000   -0.528    0.000    0
## 268  1.690    0.000    2.538    0
## 269  0.000   -0.556    0.911    0
## 270  0.000   -0.528    0.537    0
## 271  0.000    0.000    0.592    0
## 272  0.000    0.000    0.758    0
## 273 -0.129   -1.300    0.292    0
## 274  0.000   -0.573    0.388    0
## 275  0.000    0.000    0.188    0
## 276  0.000   -0.885    0.896    0
## 277  1.179   -1.189    1.969    0
## 278  1.423   -0.019    2.101    0
## 279  0.000    0.000    0.711    0
## 280  0.000    0.000    0.819    0
## 281  0.000   -0.060    1.072    0
## 282  0.000    0.000    0.000    0
## 283  0.000    0.000    0.000    0
## 284  0.000   -0.036    0.185    0
## 285  0.000   -0.052    0.000    0
## 
## 
## OMEGA EDGES:
##        Y1     Y2 n_non0 Omega_bar SE_Omega Omega_bar_non0 SE_Omega_non0
## 1  AZIlog CIPlog     91    -0.028    0.051         -0.057         0.061
## 2  AZIlog ERYlog    183     0.722    0.080          0.722         0.080
## 3  AZIlog GENlog     94    -0.043    0.065         -0.084         0.069
## 4  AZIlog TETlog    134     0.054    0.070          0.074         0.073
## 5  AZIlog FFNlog    179    -0.158    0.087         -0.162         0.084
## 6  AZIlog NALlog    124     0.002    0.094          0.004         0.114
## 7  AZIlog TELlog    183    -0.031    0.139         -0.031         0.139
## 8  AZIlog CLIlog    183     0.163    0.127          0.163         0.127
## 9  CIPlog ERYlog     44    -0.013    0.032         -0.055         0.045
## 10 CIPlog GENlog    169     0.138    0.084          0.149         0.078
## 11 CIPlog TETlog    182     0.184    0.076          0.185         0.075
## 12 CIPlog FFNlog    181     0.304    0.098          0.308         0.093
## 13 CIPlog NALlog    177     0.367    0.133          0.380         0.116
## 14 CIPlog TELlog    111     0.061    0.073          0.100         0.070
## 15 CIPlog CLIlog     26    -0.003    0.044         -0.019         0.118
## 16 ERYlog GENlog     83    -0.035    0.052         -0.077         0.051
## 17 ERYlog TETlog     88     0.029    0.048          0.060         0.055
## 18 ERYlog FFNlog     45    -0.010    0.047         -0.042         0.089
## 19 ERYlog NALlog    123    -0.045    0.085         -0.067         0.096
## 20 ERYlog TELlog    183     0.351    0.094          0.351         0.094
## 21 ERYlog CLIlog    183     0.186    0.110          0.186         0.110
## 22 GENlog TETlog    173     0.158    0.093          0.167         0.088
## 23 GENlog FFNlog    175     0.205    0.112          0.214         0.105
## 24 GENlog NALlog    158     0.120    0.137          0.139         0.138
## 25 GENlog TELlog     41     0.011    0.056          0.048         0.112
## 26 GENlog CLIlog    154     0.134    0.104          0.160         0.094
## 27 TETlog FFNlog    162     0.144    0.095          0.162         0.085
## 28 TETlog NALlog    126     0.094    0.100          0.136         0.093
## 29 TETlog TELlog    183     0.304    0.079          0.304         0.079
## 30 TETlog CLIlog    167    -0.187    0.113         -0.205         0.101
## 31 FFNlog NALlog    155     0.130    0.175          0.153         0.180
## 32 FFNlog TELlog    136     0.149    0.140          0.201         0.127
## 33 FFNlog CLIlog     84     0.053    0.093          0.116         0.108
## 34 NALlog TELlog     72    -0.009    0.107         -0.023         0.170
## 35 NALlog CLIlog    125    -0.102    0.174         -0.150         0.192
## 36 TELlog CLIlog    183     0.350    0.113          0.350         0.113
##    Omega_50 CI_lower CI_upper n_na
## 1     0.000   -0.155    0.016    0
## 2     0.725    0.564    0.859    0
## 3    -0.004   -0.208    0.000    0
## 4     0.034   -0.049    0.206    0
## 5    -0.159   -0.312    0.000    0
## 6     0.000   -0.181    0.238    0
## 7    -0.013   -0.309    0.202    0
## 8     0.182   -0.096    0.384    0
## 9     0.000   -0.118    0.000    0
## 10    0.134    0.000    0.284    0
## 11    0.178    0.047    0.360    0
## 12    0.309    0.072    0.510    0
## 13    0.396    0.000    0.542    0
## 14    0.039    0.000    0.249    0
## 15    0.000   -0.108    0.077    0
## 16    0.000   -0.164    0.000    0
## 17    0.000    0.000    0.162    0
## 18    0.000   -0.157    0.043    0
## 19   -0.008   -0.228    0.092    0
## 20    0.351    0.189    0.510    0
## 21    0.176   -0.014    0.390    0
## 22    0.155    0.000    0.341    0
## 23    0.231   -0.028    0.363    0
## 24    0.146   -0.267    0.353    0
## 25    0.000   -0.065    0.221    0
## 26    0.128    0.000    0.340    0
## 27    0.140    0.000    0.328    0
## 28    0.067    0.000    0.286    0
## 29    0.303    0.161    0.443    0
## 30   -0.196   -0.412    0.000    0
## 31    0.128   -0.367    0.454    0
## 32    0.138    0.000    0.414    0
## 33    0.000   -0.035    0.303    0
## 34    0.000   -0.275    0.245    0
## 35   -0.094   -0.420    0.295    0
## 36    0.351    0.131    0.557    0
```

#### End of RMD
