# Supplementary material for: Risk factors for fluoroquinolone- and macrolide-resistance among swine Campylobacter coli using multi-layered chain graphs
Source: PLoS Comput Biol. 2025 Aug 13;21(8):e1012797. doi: 10.1371/journal.pcbi.1012797 (PMC12373276; doi:10.1371/journal.pcbi.1012797)
Supplement: Attachment — Submitted filename: Response_to_reviewers_comments_Wang_PLOS_Comp_Biol.pdf [file pcbi.1012797.s009.pdf]

Response to reviewers comments for Risk factors for fluoroquinolone- and macrolide-resistance among swine Campylobacter coli using multi-layered chain graphs- PLOS Computational Biology

May 2025

1 Reviewer 1

The manuscript is very well written, and I appreciate the authors' transparency and willingness to share their data, code, and detailed information on the variables included in the analysis. The authors make a compelling case for the use of chain graphs in addressing these research questions, highlighting their potential utility in capturing complex dependencies. However, additional detail in the methods section would enhance clarity for readers unfamiliar with these techniques, particularly regarding how MIC was operationalized and tested for. It would also help interpret the authors' results. I have some concerns about the notion of causality given how temporality was leveraged isn't covered in the manuscript. It's also unclear if the samples are from two farms or several farms within a system and, if the latter, how clustering was handled at the farm-level. Finally, the results section would benefit from further elaboration on the nodes retained in the graphs, which would help the reader when the authors then provide insight into their roles and implications in the discussion.
Response: We thank the reviewer for the careful assessment of the paper. We have addressed the points raised above in responding to the specific reviewer questions below

1. Author Summary: Campylobacter coli needs to be italicized
   Response: Corrected

2. Results: What is the general breakdown of MICs and resistance for each farm and production system?
   Response: We have added a supplemental file (S6) that summarizes the antimicrobial susceptibility testing (AST) results by cohort.

3. How many of the isolates had high MIC values or phenotypic resistance?
   Response: The current study uses log_2MIC as the dependent variable measuring phenotypic resistance and is treated as a continuous variable. The study does not apply breakpoints or ECOFFs to interpret MICs as susceptible/intermediate/resistant. To avoid confusion, we intentionally did not report proportions of interpreted susceptibility categories in the main text. This is now clarified in Lines 97-99. We have now included new supplemental material that summarizes AST results by cohort and reports the percentage of resistant isolates.

4. Figures: The figure title or a footnote needs to include the interpretation for each node (what each three letter abbreviation for the antibiotics stands for and what the shorthand for the host exposures means) For example, are the drugs treatment or preventive use? In feed, water, injection, etc?
   Response: We have included the abbreviations for the MIC and host exposures in each figure legend.

5. The authors present very few results from the actual graphs in the results text. They broadly describe the graphs but do not present information about any of the nodes. For example, in the discussion

they talk about zinc exposure related to macrolide resistance in ABF production systems, but this isn't mentioned in the results. It is only seen by looking at the actual graphic. I would recommend briefly mentioning some of the actual results from the study like the host factor nodes that were identified within each graph and comparing/contrasting between conventional and ABF production systems, rather than only a broad overview saying no host factors were retained in the multi-scale chain graph or host factors explained little variability.
Response: We have now expanded the result section to highlight specific risk factors identified in the chain graphs. We have also included a new supplemental material, S7, that presents the full list of estimated β and Ω coefficients for all the chain graphs.

6. Methods:Generally, more information is needed on the survey and what information is included in the chain graphs: In the results, phenotypic resistance of other antimicrobial is included in the chain graphs, but that information is missing from the methods - What other antibiotics were included, why if the focus of the paper is on macrolide and fluoroquinolone-resistance, and how were these other antibiotic MICs treated/interpreted?
Response: We have expanded the method section on overview of the data to add more details on the survey as well as the phenotypic testing. The TREK Diagnostic Campylobacter panel microdilution plate cited in the methods (subsection: overview of the data) contains the list for the nine tested antibiotics. The concentrations on that panel have been added. No other phenotypic resistances were tested. As indicated in the introduction, the paper focused on macrolide and fluoroquinolone resistance, because these drug classes are used as first-line treatment for *Campylobacter* infections in humans.

7. Additionally, from the method it isn't clear to me how the MICs were treated. Were they interpreted for susceptible/resistant or were the log-transformed MIC values themselves actually used and a test for trend, or similar, done to see which risk factors are connected to increasing trend in MIC values? Were the host exposure and microbial genotype variables tested against each MIC values as their own outcome, so to speak? **Response:** MICs were treated as continuous variables. Specifically, the log2-transformed MICs were treated as continuous on a scale where a one unit change is interpretable as a 2-fold change in dilution. We did not evaluate 'trends' in MICs over time during the longitudinal sampling beyond the evaluating the sampling stage as an independent variable (see response to next comment) MICs were not interpreted as susceptible/intermediate/resistant using breakpoints or cutoffs. The results and methods sections now clarify this.

8. Finally, directed edges in these graphs represent a causal association, which would require temporality in the host exposures and resistant MICs. The authors do not talk about how this was included in the chain graphs in the methods. The farms were sampled 5 times, but at which sampling time are the MICs from? Are the MICs from all five sampling times combined? If that's the case, was the survey conducted 5 times or just once? Did it ask about past host exposures (and if so, how long before sampling) or did it simply ask if a farm had certain practices? If the survey was conducted multiple times, how did the authors account for changes in farm management practices?
Response: The isolates were sampled from pigs at all 5 time points (farrowing, early nursery, late nursery, early finishing, late finishing). The collection stage was evaluated as a predictor, but never had a $\beta > 1.0$, and in most cases, the variable for the production stage did not pass the first screening stage for the models. Because the same pigs were not necessarily sampled longitudinally, we did not evaluate AST results from previous time points as predictors for later MIC values. The survey was conducted at each stage of the product (farrowing, nursery, and finishing), and the isolates from the respective production stage were assigned the respective exposure profile. The current study makes no specific claims about causation. The directed edges in the chain graph represent non-zero coefficient values in the linear models learned from the data. They are not equivalent to directed edges in DAGs, which represent *hypothesized* causal relationships. The chain graph models aim to identify potential associations for later causal discovery, as discussed in the second-to-last paragraph of the discussion. We have also expanded this section to discuss how these results should not be interpreted as causal evidence since this algorithm does not account for effect measure modification or confounding, leading to potentially unidentified bias in the estimates.

9. Line 271: Is it 18 cohorts of 35 pigs each (in each cohort) or 18 cohorts totaling 35 across the cohorts?

Also, is this at just two facilities (one conventional and one ABF) or across how many facilities in each system was sampling done? If it was done across many facilities/farms, how did the authors control for clustering at the farm-level?
Response: There was a miscommunication during the first draft, and there were 14 cohorts, not 18. Some of the original sampled cohorts did not have sufficient data on the survey to make be included in this study. 9 cohorts were conventional, and 5 were ABF. Within a cohort, 35 pigs were sampled. We have now corrected this point in the method section.

10. Line 282: Were the same surfaces swabbed in each system?
Response: The section describing the sampling has been expanded. In general, the same surfaces were not sampled; the systems are very different, thus all the environmental samples for ABF farms were sampled outside, and inside for conventional farms.

11. Line 288: What cutoff values and methods were used for MIC preparation and interpretations?
Response: Cutoffs/breakpoints were not used (see Response to comment #7)

12. Line 289: How were the 1,466 that underwent sequencing chosen?
Response: Isolates were not sequenced because their cohort had incomplete survey data, or isolates did not grow. Some isolates were also removed because they had insufficient quality scores. We have clarified this point in the methods and discussion.

13. Line 347: If you had to stratify the graphs, what did that mean in terms of sample for each graph?
Response: Conventional and ABF farm systems are very different in terms of environment and management. Hence, estimating shared parameters across both systems would have been inappropriate. Additionally, as noted in the preceding sentence, several independent environmental variables were highly correlated with the production system. Had the data from the two production systems been pooled, we would not have been able to estimate the relative impacts of those independent variables on the phenotypic resistances. However, by stratifying on production system, the colinearity problem was solved, and we were able to include those environmental independent variables in the analysis.

2 Reviewer 2

The authors describe a novel approach for determining risk factors for antimicrobial resistance in Campylobacter coli in swine using multi-layered chain graphs. This approach compared to traditional regression models has the advantage of being able to take into account a larger number of predictor and outcome variables. Multi-layered chain graphs were built to determine risk factors in both conventional and antibiotic free swine production systems using genotypic data, environmental/host factor exposure data, and a combination of both genotypic and host factor exposures as predictors. Thank you to the authors for a well-written and interesting manuscript. One major concern is the causal relationship between exposures, genotypes, and phenotypes. Otherwise, there are only minor comments.

1. Major comments: The graphs were set up to evaluate the impact of both resistance genes or mutations and host exposures on antimicrobial phenotypes (MICs). However, this ignores the relationship between host exposures and resistance genes/mutations. If, as seen in the fluoroquinolone graphs, mutations drive much of the variation in phenotypes, then the mutation could be considered to be a mediator between host exposure and phenotype (outcome). Within a directed acyclic graph framework, conditioning on a mediator reduces the causal effect between exposure and phenotype, which could explain why no host exposures are significant in the fluoroquinolone graphs when both exposures and genotype are included. The genomic layer should be separated from the exposure layer, and edges from the exposure to genomic layer should be added.
Response: Selective pressures such as drug exposures interact with phenotypes, not genotype, hence we think it would be inappropriate to draw a DAG with genotypes or mutations acting as mediators between environmental exposures and phenotypes. Most fluoroquinolone resistance in *C. coli* are dependent on point mutations to the *gyrA* gene in the QRDR, such as the T86I mutation here. Since this

mutation is in *Camplylobacter*'s core genome, it is unlikely to be linked to any other accessory genes that may influence an isolate's fitness in the face of environmental exposures that do not act directly on phenotypic fluoroquinone resistance, and therefore would not have any edges between those nodes representing environmental exposures and CIP. It was, however, surprising that enrofloxacin use did not appear to be associated with increased fluoroquinolone MICs in the conventional systems, though the frequency of the gyrA mutation in conventional systems (137/683, 20%) than in ABF systems (5/360, 1%). However, other host exposures such as lab confirmed respiratory pathogen and other antimicrobial exposures were associated with CIP.

2. Minor comments Author summary: Campylobacter coli is not italicized. Line 10: Campylobacter is not italicized
   Response: Corrected

3. Introduction: Lines 66-77: What is the hypothesis for fluoroquinolone resistance? It is mentioned in the discussion, but it would be helpful for readers to state it here along with the macrolide resistance hypothesis.
   Response: A sentence regarding the hypothesis for fluoroquinolone resistance has been added.

   Methods:

4. Line 271: How many of the 18 cohorts were from ABF farms vs conventional?
   Response: There was a miscommunication during the first draft, and there were 14 cohorts, not 18. Some of the original sampled cohorts did not have sufficient data on the survey to make be included in this study. 9 cohorts were conventional, and 5 were ABF. This has been added to the Methods section (L336-337)

5. Line 289: How were the isolates selected for sequencing? Randomly? Do they represent all isolates? If the selection is not representative of all the isolates studied, then this should be discussed as a limitation of the study and the bias estimated, if possible.
   Response: Isolates were not sequenced because their cohort had incomplete survey data, or isolates did not grow. Some isolates were also removed because they had insufficient quality scores. We have clarified this point in the methods and discussion.

6. Line 317: How was one variable in the correlated pair selected for exclusion over the other?
   Response: Variables with multiple high correlations were removed first, and variables thought to be important were conserved where possible. This is now explained in the manuscript.

7. Line 339: In MIC quality control, often one 2-fold dilution is acceptable variation. Therefore, shouldn't a $\beta > 2$ be the cut-off for a microbiologically detectable change?
   Response: It is correct that broth microdilution is considered to be in specification if the result is within one 2-fold dilution of the true value. However, this is an issue of imprecision in the method and so long as the error is random and non-differential (which we did assume) the imprecision will only impact the variance of estimated means and not the means themselves. Ultimately, it is the means themselves that we are comparing, and given a sufficiently large sample, which we believe we have, the means estimated should be converging to the population means, and we believe that a one 2-fold difference in mean MIC is still a meaningful difference. Looking for a larger differences would reduce our power to detect them.

8. It is unclear how the fluoroquinolone and macrolide specific graphs were created as only the general approach is described. For example, for the fluoroquinolone graph, were only the fluoroquinolone phenotypes included in the outcome layer (line 352)? Or were only the fluoroquinolone phenotypes eligible for connection to other layers?
   Response: The chain graphs contain all the nine phenotypic resistances. We only plot the resistance of interest because a plot of all the chain graph nodes and edges is very difficult to visualize. The subgraphs in the figure represent the resistance nodes of interest (CIP & NAL in the fluoroquinolone subgraphs and ERY & AZI in the macrolide subgraphs). An explanation of the subgraph construction has been added to each legend.

   Results:

9. Line 92: include which software was used to screen sequences for AMR genes. It was detailed in the methods, but since the results come first in this manuscript, it would help the reader.
Response: We have added now the AMRFinderPlus to the sentence.

10. Line 96: Would it be possible to change "predicted layers" to "predictor layers" to avoid confusion with predictors vs outcome variables?
Response: Corrected

11. Line 102: Were β and Ω supposed to be represented in Figure 1?
Response: Both β and Ω are represented in Figure 1: β contains the directed edges and Ω contains the undirected edges. A sentence explaining this has been added to the figure legend.

12. Line 104: Please include a brief explanation of what λ and ρ are in the results section since the methods description comes later.
Response: A brief explanation on L1 penalties has been added to the result section.

13. Line 105: How was the penalty of 0.25 selected?
Response: This is covered in the Methods section, lines 448-449, and a sentence has been added to the results section.

14. The edges between MICs (Ω edges) could represent expected cross-resistance phenotypes of antimicrobial drugs within the same classes (for example, ciprofloxacin and nalidixic acid or azithromycin and erythromycin). Calculating density of these edges within-classes vs between-classes could provide additional insight into differences in resistance relationships across systems, and exposures.
Response: This approach has been used in our previous studies using a similar method employing only the Ω portion of the graph (Love, et al. Phenotypical resistance correlation networks for 10 non-typhoidal Salmonella subpopulations in an active antimicrobial surveillance programme. Epi Inf) and found that the denser subgraphs aligned well to components identified via principal component analysis and known groups of genes on mobile cassettes, e.g., ACSSuT MDR Salmonella Typhimurium DT193. While this analysis may be insightful, it is beyond the scope of what we aimed to include in the current paper.

15. Figure 2/3/4/5: Can the phenotype node layout be the same in each panel for easier comparisons? Could the coefficients be written on each edge?
Response: While we agree it would be ideal to have the nodes in the same position across all the figures, that strategy is incompatible with the number and variety of environmental nodes in some of the displayed subgraphs. We have done our best to make each figure as comprehensible on its own while fitting into the larger paper. Similarly, some subgraphs have too many edges to clearly add the coefficients. We have added a new supplement material that includes all the estimated coefficients.

16. Lines 136-137: It is unclear what is meant by including the beta edges to clindamycin and telithromycin. β edges are shown in black and connect different graphs, but the wording implies connecting different MICs, which would be the Ω edges. The confusion here relates to the lack of specificity in how the drug-specific graphs were developed in the methods.
Response: This sentence refers to the β edges from from A2075G to TEL and CLI, since these drugs and ERY and AZI are all in the MLS family that targets the *23S* ribosomal subunit. This has been clarified in the manuscript.

17. Table 2: In the caption, please list which supplemental material appendix specifically contains the independent and dependent variables included in the models.
Response: The table caption now refers to supplemental material S2.

18. Is it possible to put the antimicrobial name abbreviations at the bottom of figures to make it easier for the reader rather than searching in the body of the text?
Response: The antimicrobial name abbreviations have been added to the figure legends.

19. Why is the variance explained by prediction layers Rx2 for ciprofloxacin ABF genotypes and host exposures together lower than Rx2 for genotypes or host exposures alone?

Response: This appears to be a transcription error. The appropriate values are now reflected in the table.

20. Please add descriptive statistics on the MIC distributions for each drug and system, similar to Table S2. It is also helpful to include the range of MIC values tested on the Sensititre plate, as this creates censoring of the data.
Response: A new supplemental file (**S6**) has been added with the distribution of MICs for each cohort. The MIC ranges have been added in the methods section.

Discussion:

21. Line 185: Give the table that the R235% statistic was cited from.
Response: The reference to table 2 has been added.

22. Lines 196-200: Could a similar mechanism be behind the relationship between beta-lactamase, aminoglycoside, and tetracycline resistance genes and macrolide MICs?
Response: It is plausible that the relationship between some of the genes encoded resistance for other drug class and macrolide resistance is indirectly driven by the pattern of antimicrobial use. We have expanded the discussion to address this point.

23. Lines 239-241: What is the impact of a smaller or larger penalty on the presented results?
Response: Smaller penalties remove fewer edges and produce denser graphs and models, and higher penalties remove more edges and produce less dense. This is now mentioned in the discussion (L-275-277).

24. Line 246: Which genotype-phenotype relationships are novel? The resistance genes and mutations studied are known to result in phenotypic resistance or reduced susceptibility.
Response: While the identified genes and mutations are known to confer resistance, some of the edges (or lack of) were not expected. For example, the the A103V amino acid substitution of the L22 protein in the 50S ribosomal subunit had been previously described as conferring resistance to macrolides, but in our study, it was not associated to macrolides resistance. *aph(3')-IIIA* and *aad9* genes were positively associated to macrolide resistance, but these genes encode enzymes that confer resistance to aminoglycoside antibiotics, and have not been shown to directly confer macrolide resistance. These findings warrant further research; it is unclear why there is a strong association between genes conferring aminoglycoside resistance and phenotypic macrolide resistance. We have expanded this point in the discussion to include these examples (L-282-297).

25. Conclusion: Please include a conclusion specific to the results, beyond just the novelty of the method.
Response: We have expanded the conclusion to address this comment
