# Supplementary material for: Risk factors for fluoroquinolone- and macrolide-resistance among swine Campylobacter coli using multi-layered chain graphs
Source: PLoS Comput Biol. 2025 Aug 13;21(8):e1012797. doi: 10.1371/journal.pcbi.1012797 (PMC12373276; doi:10.1371/journal.pcbi.1012797)
Supplement: Attachment — Submitted filename: Response_to_reviewers_comments_Wang_PLOS_Comp_Biol_R2.pdf [file pcbi.1012797.s010.pdf]

Response to reviewers comments for Risk factors for fluoroquinolone- and macrolide-resistance among swine Campylobacter coli using multi-layered chain graphs- PLOS Computational Biology

July 2025

Reviewer 1

Thank you for the thoughtful changes and additions to the manuscript based on reviewer comments. I believe the manuscript is much improved and have only a few additional minor comments that need to be addressed:

1. If qualitative interpretations for MICs were not used, then "phenotypic resistance" is not a correct term to use throughout the manuscript. It appears the authors only assessed resistance through genotypic resistance. This needs to be updated throughout the manuscript because it is confusing to see "phenotypic resistance" when qualitative interpretations were not used.

   Response: We have replaced the term "phenotypic resistance" with "antimicrobial susceptibility" throughout the manuscript.

2. Results, line 85: given the topic of antibiotic use in livestock is somewhat contentious and highly scrutinized, I would recommend the authors be very deliberate and careful with their language here. In the US, using medically important antibiotics for growth promotion is illegal (although I recognize the data for this study was collected before that law was enacted). It might be worth updating this to clarify that subtlety–potentially even just saying something about how as this was prior to the U.S. law change making MIADs... or something similar.

   Response: We include the following statement to clarify this point: " These data were collected before the 2017 implementation of the new Veterinary Feed Directive, which eliminated the use of medically important antimicrobial drugs for animal growth promotion."

3. Results line 133: Is this a typo? Should it say, "The host exposures WITH the higher coefficients"? Methods line 359: Is there a word missing between "the" and "on"?

   Response: We have corrected the indicated typos.

Reviewer 2

1. Lines 109-114: please review for typos. "less than penalty", "evaluated using on" Lines 132-135: please review for typos Line 286: please review for typos. "it is worth consider" Line 359: please review for typos. "The on these plates"

   Response: We have corrected the indicated typos.
