# Supplementary material for: Risk factors for fluoroquinolone- and macrolide-resistance among swine Campylobacter coli using multi-layered chain graphs
Source: PLoS Comput Biol. 2025 Aug 13;21(8):e1012797. doi: 10.1371/journal.pcbi.1012797 (PMC12373276; doi:10.1371/journal.pcbi.1012797)
Supplement: Attachment — Submitted filename: Plos_Review.docx [file pcbi.1012797.s008.docx]

Reviewer notes:

The manuscript is very well written, and I appreciate the authors’ transparency and willingness to share their data, code, and detailed information on the variables included in the analysis. The authors make a compelling case for the use of chain graphs in addressing these research questions, highlighting their potential utility in capturing complex dependencies. However, additional detail in the methods section would enhance clarity for readers unfamiliar with these techniques, particularly regarding how MIC was operationalized and tested for. It would also help interpret the authors’ results. I have some concerns about the notion of causality given how temporality was leveraged isn’t covered in the manuscript. It’s also unclear if the samples are from two farms or several farms within a system and, if the latter, how clustering was handled at the farm-level. Finally, the results section would benefit from further elaboration on the nodes retained in the graphs, which would help the reader when the authors then provide insight into their roles and implications in the discussion.

Author Summary: *Campylobacter coli* needs to be italicized

Results:

What is the general breakdown of MICs and resistance for each farm and production system? How many of the isolates had high MIC values or phenotypic resistance?

Figures: The figure title or a footnote needs to include the interpretation for each node (what each three letter abbreviation for the antibiotics stands for and what the shorthand for the host exposures means) For example, are the drugs treatment or preventive use? In feed, water, injection, etc?

The authors present very few results from the actual graphs in the results text. They broadly describe the graphs but do not present information about any of the nodes. For example, in the discussion they talk about zinc exposure related to macrolide resistance in ABF production systems, but this isn’t mentioned in the results. It is only seen by looking at the actual graphic. I would recommend briefly mentioning some of the actual results from the study like the host factor nodes that were identified within each graph and comparing/contrasting between conventional and ABF production systems, rather than only a broad overview saying no host factors were retained in the multi-scale chain graph or host factors explained little variability.

Methods:

Generally, more information is needed on the survey and what information is included in the chain graphs: In the results, phenotypic resistance of other antimicrobial is included in the chain graphs, but that information is missing from the methods - What other antibiotics were included, why if the focus of the paper is on macrolide and fluoroquinolone-resistance, and how were these other antibiotic MICs treated/interpreted?

Additionally, from the method it isn’t clear to me how the MICs were treated. Were they interpreted for susceptible/resistant or were the log-transformed MIC values themselves actually used and a test for trend, or similar, done to see which risk factors are connected to increasing trend in MIC values? Were the host exposure and microbial genotype variables tested against each MIC values as their own outcome, so to speak?

Finally, directed edges in these graphs represent a causal association, which would require temporality in the host exposures and resistant MICs. The authors do not talk about how this was included in the chain graphs in the methods. The farms were sampled 5 times, but at which sampling time are the MICs from? Are the MICs from all five sampling times combined? If that’s the case, was the survey conducted 5 times or just once? Did it ask about past host exposures (and if so, how long before sampling) or did it simply ask if a farm had certain practices? If the survey was conducted multiple times, how did the authors account for changes in farm management practices?

Line 271: Is it 18 cohorts of 35 pigs each (in each cohort) or 18 cohorts totaling 35 across the cohorts? Also, is this at just two facilities (one conventional and one ABF) or across how many facilities in each system was sampling done? If it was done across many facilities/farms, how did the authors control for clustering at the farm-level?

Line 282: Were the same surfaces swabbed in each system?

Line 288: What cutoff values and methods were used for MIC preparation and interpretations?

Line 289: How were the 1,466 that underwent sequencing chosen?

Line 347: If you had to stratify the graphs, what did that mean in terms of sample for each graph?
